# Sensitive CAR T cells redefine targetable CD70 expression in solid tumors

**DOI:** 10.1126/science.adv7378

**Published:** 2026-02-26

**Authors:** Sophie A. Hanina, Tyler Park, Michael Lopez, Vinagolu K. Rajasekhar, Jorge Mansilla-Soto, Sascha Haubner, Huiyong Zhao, Friederike Kogel, Sarah Nataraj, Priyam Banerjee, Richard Koche, Pierre-Jacques Hamard, Zeynep C. Tarcan, Dennis S. Chi, Dmitriy Zamarin, John H. Healey, Elisa de Stanchina, Robert J. Motzer, Ritesh R. Kotecha, A. Ari Hakimi, Christina S. Leslie, Michel Sadelain

**Affiliations:** 1 Columbia Initiative in Cell Engineering and Therapy (CICET), Department of Medicine, Columbia University Irving Medical Center, Columbia University, New York, NY USA.; 2 Computational and Systems Biology Program, Memorial Sloan Kettering Cancer Center, New York, NY, USA.; 3 Orthopedic Service, Department of Surgery, Memorial Sloan Kettering Cancer Center, New York, NY, USA.; 4 Center for Cell Engineering, Memorial Sloan Kettering Cancer Center, New York, NY, USA.; 5 Antitumor Assessment Core Facility, Molecular Pharmacology Program, Memorial Sloan Kettering Cancer Center, New York, NY, USA.; 6 Bio-Imaging Resource Center, The Rockefeller University, New York, NY USA.; 7 Center for Epigenetics Research, Memorial Sloan Kettering Cancer Center, New York, NY, USA.; 8 Department of Pathology, Memorial Sloan Kettering Cancer Center, New York, NY, USA.; 9 Gynecology Service, Department of Surgery, Memorial Sloan Kettering Cancer Center, New York, NY, USA.; 10 Department of Obstetrics and Gynecology, Weill Cornell Medical College, New York, NY, USA.; 11 Tisch Cancer Institute, Icahn School of Medicine at Mount Sinai, New York, NY, USA.; 12 Genitourinary Oncology Service, Department of Medicine, Memorial Sloan Kettering Cancer Center, New York, NY, USA.; 13 Urology Service, Department of Surgery, Memorial Sloan Kettering Cancer Center, New York, NY, USA.; 14 Human Oncology and Pathogenesis Program, Memorial Sloan Kettering Cancer Center, New York, NY, USA.

## Abstract

**INTRODUCTION:**

Chimeric antigen receptor (CAR) T cells are living drugs that are programmed to eliminate cancer cells. These cell therapies are effective in hematological malignancies but currently fail in solid tumors. One challenging obstacle to the success of CAR therapy owes to the inconsistent expression of target antigen across the population of tumor cells, which results in incomplete tumor elimination and overall disease progression.

**RATIONALE:**

The properties of an ideal target include (i) expression on all tumor cells, (ii) restricted expression on dispensable normal cells, and (iii) common to many tumor types. CD70 is potentially one such target, fulfilling criteria (ii) and (iii), but its expression pattern (i) is typically heterogeneous in solid tumors.

We hypothesized that tumor cells, which appear negative for CD70 in tumors containing some CD70-positive tumor cells, may in fact express CD70 at a level that is below the sensitivity of conventional detection methods and conventional CAR T cells. If CD70 is present on all tumor cells, albeit at very low levels in some cells, a more sensitive receptor could potentially eliminate the entire tumor.

**RESULTS:**

We developed representative CD70-heterogeneous tumor models of kidney, ovarian, and pancreatic cancer using patient-derived xenografts to investigate mechanisms of CD70-CAR T cell resistance in solid tumors. Conventional CD70-CAR T cell designs selectively eliminated CD70-positive tumor cells but failed to eradicate CD70-negative tumor, mirroring clinical observations. By contrast, when we used a more sensitive CAR T cell design, termed HLA-independent T cell (HIT) receptor, CD70-targeted HIT T cells effectively eradicated heterogeneous tumors across all three tumor types.

We indeed determined that seemingly CD70-negative tumor cells retain residual CD70 expression due to epigenetic silencing by EZH2-mediated H3K27me3, which accounts for CAR resistance but HIT susceptibility. To determine whether *CD70* is epigenetically regulated in primary kidney cancer patient tumors, we profiled *CD70* chromatin accessibility and transcriptional expression at a single-cell level. Patient tumor cells with apparent *CD70* negativity exhibited an epigenetic signature similar to that of our patient–derived xenograft models, thus suggesting low-level expression and HIT sensitivity. To assess low-level expression that is below the limits of conventional detection methods in normal tissues, we used *CD70* chromatin accessibility as a surrogate for predicting HIT responsiveness. CD70 was largely absent from most adult normal tissues—excluding activated immune cells, which acquire CD70 expression upon activation. In accord with these findings, the toxicity of CD70-HIT T cells was not greater than that of CD70-CAR or CD19-CAR T cells in cytotoxicity assays and in vivo in a humanized model, further validating chromatin accessibility predictions and the safety of CD70-HIT T cells.

**CONCLUSION:**

Our work reveals that CD70 expression within CD70-heterogeneous solid tumors is pan-cellular, though some cells may appear to be negative by standard detection assays. These findings position CD70 as an excellent immunotherapeutic target, but only if a highly sensitive targeting receptor is used. This study further illustrates the value of carefully investigating the regulation of CAR target expression. As we show here for CD70, epigenetic regulation may drive differential expression across different anatomic sites, determine responsiveness to distinct CAR designs, and inform the design of improved targeting strategies. □

## Abstract (summary)

Solid tumor antigen heterogeneity is a major challenge for cancer immunotherapies, including chimeric antigen receptor (Car) T cells. unlike CD19 for b cell malignancies, no target with pan-cellular expression in solid tumors and absence in normal vital cells has been identified. CD70 is a promising candidate, physiologically confined to immune cell subsets and aberrantly expressed in many cancers. We show that heterogeneous CD70 expression in tumors is epigenetically regulated, ranging from high to very low in individual cells, appearing negative by conventional detection methods. using a highly sensitive CD70 receptor, HLa-independent T cell (HIT) receptor coexpressing CD80 and 4–1bbL for costimulation, we efficiently eliminated CD70-heterogeneous tumors that evade prototypic Car T cells. These findings provide a potential strategy to treat a broad range of solid tumors.

## Editor’s summary

Chimeric antigen receptor (CAR) T cells represent a form of cancer immunotherapy that can be genetically engineered from human T lymphocytes. CAR T cells have been transformative for the treatment of B cell malignancies, yet targeting of solid tumors remains a challenge. One hurdle is the lack of available targets with pan-tumor expression, resulting in incomplete tumor responses. Hanina *et al*. report that epigenetic regulation of CD70, which is expressed heterogeneously in many cancers, leads to stealth CD70 expression in tumor cells that may appear CD70-negative. Although CD70 expression levels can be too low to be eliminated by conventional CAR T cell therapy, a more sensitive antigen receptor led to tumor elimination in patient-derived models of kidney, ovarian, and pancreatic cancer. — Priscilla N. Kelly

## Graphical Abstract

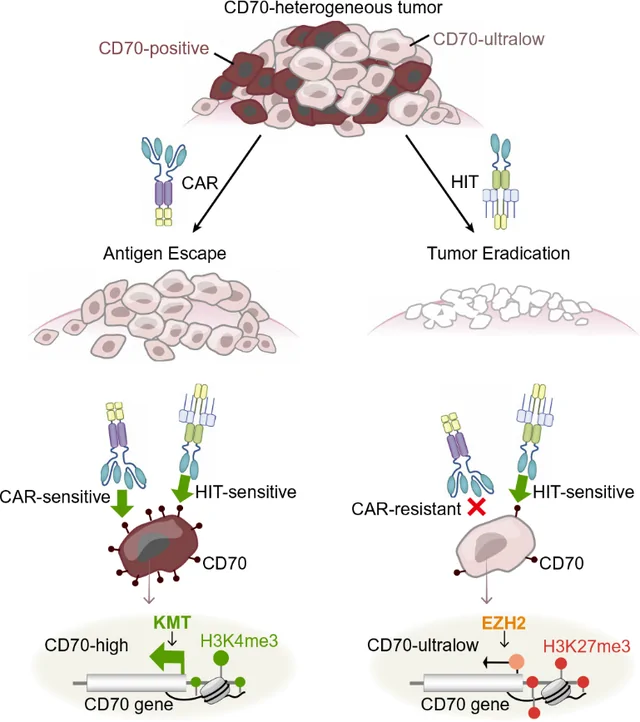

**Overcoming solid tumor antigen heterogeneity with sensitive CAR T cells.** In CD70-heterogeneous solid tumors, CD70-CAR T cells eliminate CD70-positive tumor cells but commonly fail to achieve complete tumor clearance. The escaping tumor cells, which appear to be CD70-negative, actually express ultralow levels of CD70 that are undetected by conventional detection assays and commonly used CARs. However, more sensitive receptors, such as HIT receptors, can recognize and eliminate these cells, achieving complete tumor eradication. CD70 expression is thus pseudo-heterogeneous, ranging from high to ultralow owing to epigenetic silencing of *CD70* by enhancer of zeste homolog 2 (EZH2)–mediated trimethylation of lysine 27 on histone H3 (H3K27me3). A broad range of CD70-heterogeneous solid tumors may therefore be efficiently targeted, provided that the T cells are engineered with sensitive receptors such as HIT receptors. KMT, lysine methyltransferase.

Chimeric antigen receptors (CARs) are synthetic receptors that redirect the specificity and augment the functions of immune effector cells (1). CAR T cells targeting CD19 have revolutionized the treatment of B cell malignancies, achieving in some instances durable complete remissions of otherwise refractory disease (2). However, CAR T cells have not to date obtained comparable responses in solid tumors. Unlike CAR targets in B cell leukemias and lymphomas (2), no equivalent pan-cancer cell surface target with limited expression in normal tissues has yet been identified in solid tumors (3–6). Efficacious targeting of solid tumors is thus compromised by heterogeneous target expression, which limits CAR T cell efficacy, and target expression in normal tissues, which precludes the use of potent T cells to avert unacceptable toxicities. There is at present a dearth of common, effective CAR targets in solid tumors.

CD70, a member of the tumor necrosis factor (TNF) superfamily and the ligand for CD27 (7, 8), is an attractive CAR target because of its aberrant up-regulation in a variety of solid tumors (9–15), whereas its physiological expression is restricted to subsets of activated immune cells (16–18). Its prevalence in 70 to 80% of clear cell renal cell carcinoma (ccRCC) and ovarian cancer, and 25% of pancreatic cancer (14, 15, 19), makes CD70 a highly valuable target across a range of cancers. However, CD70 expression in tumors is typically heterogeneous, with some tumor cells exhibiting high expression and others, often accounting for the majority of tumor cells, appearing low or negative (19–22). Not surprisingly, the clinical responses obtained in patients with advanced ccRCC who were treated with CD70-targeted CAR T cells have been modest (23–25).

To study the limitations of CAR therapy in these solid tumors, we developed accurate preclinical models that recapitulate CD70 tumoral antigen heterogeneity observed in kidney cancer patients. However, we found that apparent CD70-negative tumor cells do in fact express low levels of CD70, though not at a level high enough to be eliminated by conventional CAR T cells. We could leverage this pan-tumor cell expression of CD70 by adapting CAR design for more sensitive detection. We investigated an HLA-independent T cell (HIT) receptor (26), which substitutes the antigen recognition domain of a T cell receptor (TCR) with that of a CAR, thereby co-opting the downstream signaling afforded by the CD3 complex (27). Unlike CD70-CAR T cells, CD70-HIT T cells completely eradicated CD70-heterogeneous tumor models of renal cell carcinoma, ovarian cancer, and pancreatic cancer.

We further found that the *CD70* locus in apparent-negative tumor cells is epigenetically silenced by enhancer of zeste homolog 2 (EZH2)–mediated histone H3 lysine 27 trimethylation (H3K27me3), still allowing for very low expression. We thus harnessed chromatin accessibility to infer an epigenetic signature that predicts low-level CD70 expression in a cohort of patient samples. Overall, our results demonstrate that CD70 target expression in CD70-heterogeneous tumors is pan-cellular, making a broad range of tumors therapeutically targetable provided a highly sensitive engineered CAR is used.

## CD70-CAR T cells fail to eradicate CD70-heterogeneous kidney tumors

**Fig. 1.**
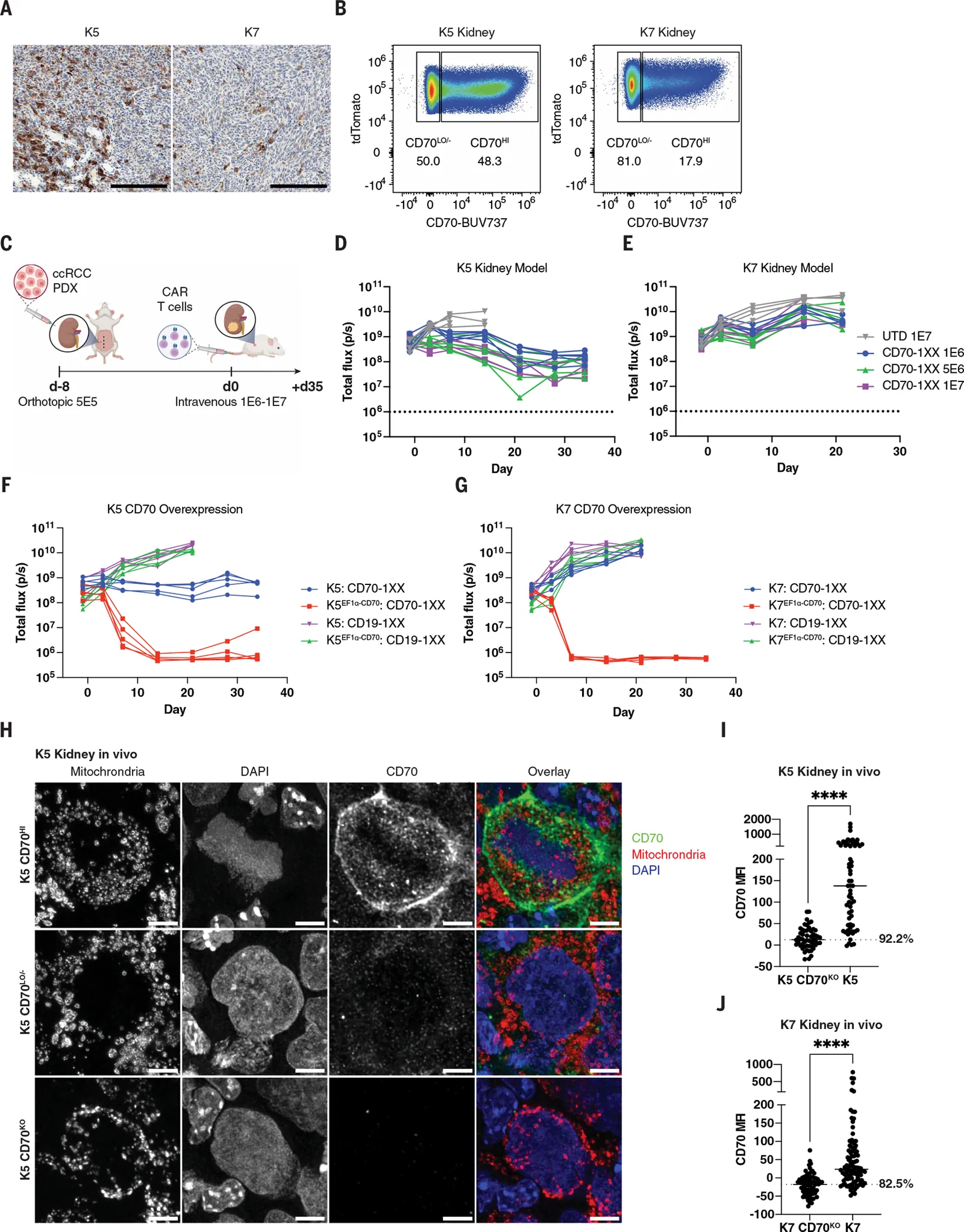
CD70-CAR T cells fail to eradicate CD70-heterogeneous kidney tumors. (**A**) CD70 immunohistochemistry of CD70-heterogeneous K5 and K7 PDX ccRCC tumors at the kidney site. Scale bar, 200 μM. (**B**) Representative flow cytometric analysis of CD70 expression on K5 and K7 tumors at kidney site 15 days after tumor implantation. (**C**) Diagram depicting in vivo CAR T treatment of ccRCC kidney model. K5 or K7 ccRCC tumor is administered orthotopically (5 × 10^5^ cells per mouse) into the renal subcapsule of NSG mice with tumor growth predominantly in the kidneys. CAR T cell timing, dose, and route of administration is shown. (**D** and **E**) K5 kidney model (D) and K7 kidney model (E) treated with 1 × 10^6^ to 1 × 10^7^ CD70–1XX CAR, or 1 × 10^7^ untransduced (UTD) T cells. Tumor burden (total flux) was monitored using bioluminescence imaging. *n* = 5 mice per group. Donor 4 in (D); representative of two independent in vivo experiments (with two different donors). Donor 5 in (E); representative of four independent in vivo experiments (with four different donors). (**F** and **G**) Constitutive overexpression of CD70 on tumor at kidney site, in K5 (“K5^EF1α-CD70^”) (F) and K7 (“K7 ^EF1α-CD70^”) (G) treated with 5 × 10^6^ CD70–1XX or CD19–1XX CAR T cells (donor 10). Tumor burden (total flux) is shown. *n* = 5 mice per group. Representative of two independent in vivo experiments (with two different donors). (**H**) Representative confocal micrographs of CD70^HI^ and CD70^LO/−^ K5 tumor cells at kidney site, and CD70-knockout (CD70^KO^) K5 tumor cells at kidney site, 15 days after tumor implantation. Overlay shows human mitochondria (red), CD70 (green), and DAPI (4′,6-diamidino-2-phenylindole, blue). Scale bar, 5 μM. (**I** and **J**) Mean fluorescence intensity (MFI) of CD70 on individual tumor cells at kidney site for K5 (I) and K7 (J). Horizontal bars represent median. Dotted line represents the median CD70 MFI on CD70^KO^ PDX tumor cells. Percentages displayed are the fraction of wild-type tumor cells with a CD70 MFI above the median of CD70^KO^ tumor cells. ****P < 0.0001, two-tailed *t* test.

To test CD70-CAR T cells in solid tumor models that reflect patient characteristics, we generated two ccRCC patient–derived xenografts (PDX), termed K5 and K7, from multiply treatment-resistant patients (Table 1). K5 is a classic *VHL*-altered ccRCC, and K7 is a more aggressive *NF2*-altered ccRCC tumor with sarcomatoid features (fig. S1A). Both K5 and K7 expressed CD70 in culture (fig. S1B) and were lysed by T cells expressing CD70-specific CARs (figs. S1, C and D, and S3, A and B). NALM6, a CD70-negative cell line, was not lysed (fig. S1, E and F). We established a kidney model by implanting tumor orthotopically into the renal subcapsule (fig. S1G; size of tumor at time of T cell infusion is shown in fig. S1H), which displayed CD70-heterogeneous expression by immunohistochemistry (Fig. 1A) and flow cytometry (Fig. 1B). To mitigate alloreactivity and fratricide, both *TRAC* and *CD70* loci were ablated in all infused T cells (fig. S2), as we found that ablating CD70 improved CD70-CAR T cell efficacy (fig. S2C) without impairing CAR T cell function (fig. S2D).

Treatment of CD70-heterogeneous kidney tumors with different CD70-CAR T cell designs—CD70–28z, CD70-BBz, and CD70–1XX—failed to eradicate tumor even at very high CAR T cell doses (10 million cells per mouse) (Fig. 1, C to E, and fig. S3, C and D). CD70-CAR T cells only delayed tumor progression in K5 (Fig. 1D and fig. S3C) and outright failed in the more aggressive K7 kidney model (Fig. 1E and fig. S3D), consistent with the higher percentage of cells expressing CD70 in the K5 kidney model (≈50%) compared with K7 (≈18%) (Fig. 1, A and B). When ccRCC tumor was administered intravenously to establish a lung model (fig. S2B), all three CD70-CAR T cells induced a comparable initial response, but the 1XX provided the most durable response (figs. S3, E and F, and S4, A and B). We therefore focused on the CD70–1XX in subsequent studies.

When the lung and kidney model were combined so that mice bore tumor at both sites (Lung + Kidney), CD70-CAR T cells again elicited rapid tumor clearance in the lung but not at the kidney site (fig. S4C). CD70-CAR T cells that were labeled with Gaussia luciferase were detected in both lung and tumor-bearing kidney (fig. S4C), establishing that CD70-CAR T cells did not fail to reach the kidney tumor. Contrasting with the kidney site, tumor cells in the lung showed robust CD70 expression (fig. S4D), suggesting that CD70 level and not an inability of CD70-CAR T cells to traffic to the kidney site accounted for CAR-T cell failure. Further corroborating these findings, analysis of CD70 antigen profiles in refractory kidney tumor after CD70-CAR T cell treatment showed that CD70-positive tumor was cleared, but a low or negative fraction remained (fig. S4E). Finally, constitutive CD70 expression at the kidney site (using a lentiviral vector encoding the EF1α promoter), which maintained homogeneous CD70 levels in the kidney tumor (fig. S4, G and I), allowed for CD70-CAR T treatment to achieve complete tumor eradication (Fig. 1, F and G), confirming that CD70 levels dictated tumor responsiveness to CAR therapy.

## CD70 is expressed in tumor cells appearing negative

To characterize CD70 expression in heterogeneous K5 and K7 PDX kidney tumors, we used confocal and stimulated emission depletion (STED) microscopy to image in the far-red channel to eliminate cell autofluorescence to improve detection of low-level signal above background noise (Fig. 1H). CD70 mean fluorescence intensity (MFI) was quantified in individual cells from wild-type and CRISPR-Cas9 *CD70*-edited PDX kidney tumor (Fig. 1, I and J, and fig S5). We detected a spectrum of CD70 expression, with 92.2% of K5 tumor cells and 82.5% of K7 tumor cells above the median MFI of *CD70*-edited tumor (Fig. 1, I and J). We thus found that tumor cells that appear CD70-negative exhibit higher MFI than CD70-knockout (CD70^KO^) tumor and therefore still have CD70 present at very low levels.

### Sensitive HLA-independent T cell receptors detect low-level CD70

**Fig. 2.**
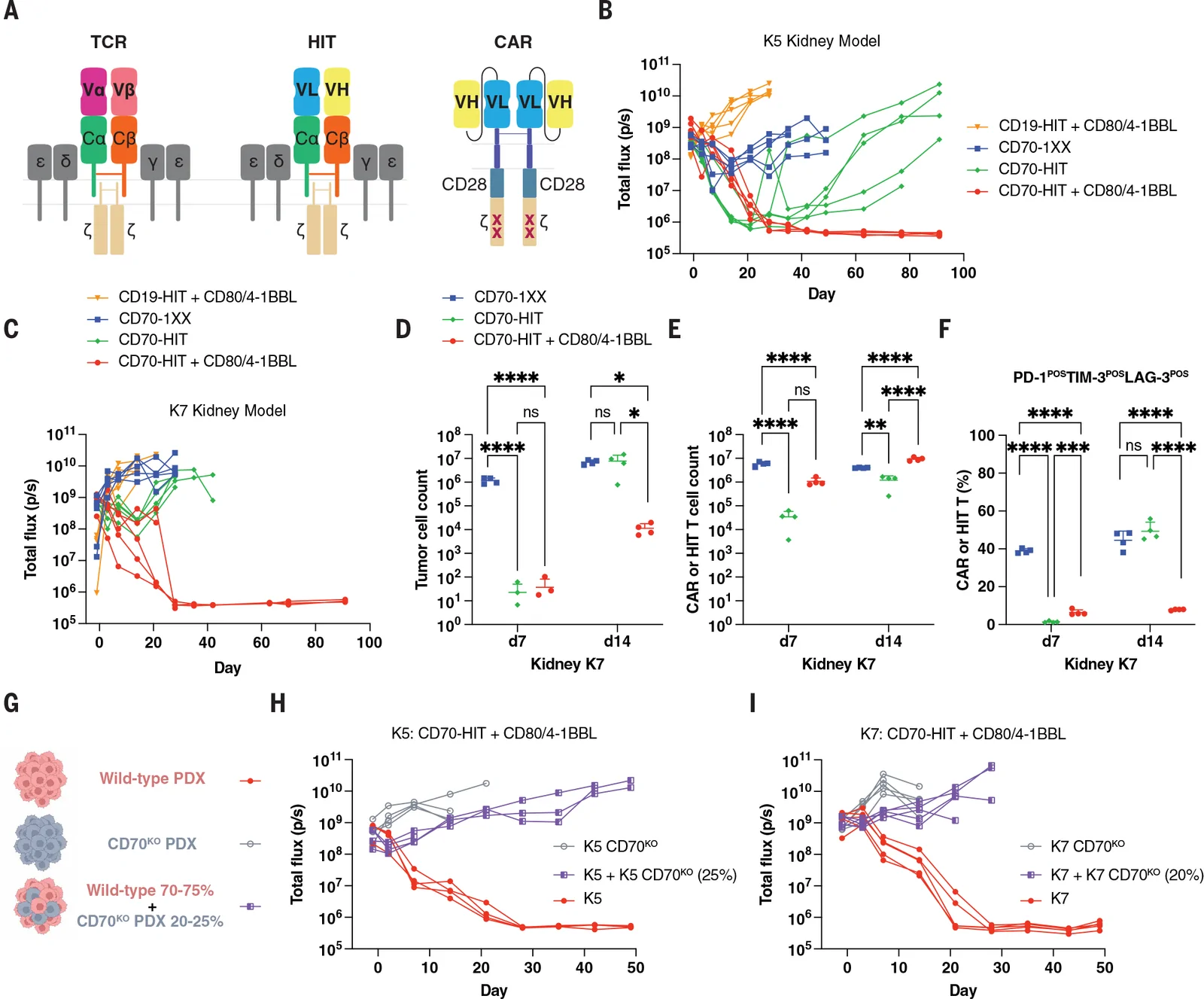
Sensitive HLA-independent T cell receptors detect low-level CD70 expressed on apparent CD70-negative tumor cells. (**A**) Diagram depicting a CD70-HIT receptor. The same variable heavy (VH) and variable light (VL) domains of the CD70–1XX CAR replace the variable α (Vα) and variable β (Vβ) of a TCR to form the HIT heterodimer. HIT T cells are engineered by disrupting TCRα expression, and integrating the VH fused to the constant β (Cβ) domain of the TCR and the VL into the *TRAC* locus where expression is driven by the endogenous *TCR*α promoter. (**B** and **C**) K5 kidney model (B) and K7 kidney model (C) treated with 2.5 × 10^6^ CD70–1XX CAR, CD70-HIT, CD70-HIT endowed with CD80 and 4–1BBL (“CD80/4–1BBL”) costimulation or CD19-HIT+CD80/4–1BBL T cells. Tumor burden (total flux) is shown. *n* = 5 mice per group. Donor 11 in (B); representative of five independent in vivo experiments (with five different donors). Donor 12 in (C); representative of four independent in vivo experiments (with four different donors). (**D** to **F**) The number of K7 tumor cells (D), the number of CAR or HIT T cells (E), and the percentage of CAR or HIT T cells expressing inhibitory receptors (PD-1, LAG-3 and TIM-3) (F) isolated from kidney tumors of treated mice from an independent experiment (donor 13) at day 7 and day 14 after CAR or HIT T cell administration. *n* = 4 mice per group; mean ± SD; ns, not significant; *P < 0.05; **P < 0.01; ***P < 0.001; ****P < 0.0001; one-way analysis of variance (ANOVA). Characterization of preinfusion donor 13 T cells is shown in fig. S6. (**G**) Diagram depicting different in vivo kidney tumor conditions used in (H) and (I): wild-type PDX tumor; CD70^KO^ PDX tumor; and mixing studies in which 70 to 75% wild-type PDX tumor is mixed with 20 to 25% CD70^KO^ tumor. (**H** and **I**) Tumor burden (total flux) in K5 kidney model (H) and K7 kidney model (I) is shown comparing the in vivo tumor clearance of CD70-HIT+CD80/4–1BBL T cells in mice bearing the different conditions illustrated in (G). HIT T cell dose = 2.5 × 10^6^. *n* = 5 mice per group. Donor 16 in (H). Donor 17 in (I); representative of three independent in vivo experiments (with three different donors).

We hypothesized that a more sensitive CAR design, such as an HLA-independent T cell (HIT) receptor (26), could detect and eliminate tumor cells with low levels of CD70 (Fig. 2A). Notably, treatment with CD70-HIT T cells in vivo resulted in a rapid and deep tumor elimination of K5 kidney tumor, in contrast to CD70-CAR T cells (Fig. 2B). For K7 kidney tumor, CD70-HIT T cells conferred an improvement in tumor response compared to CD70-CAR T cells (Fig. 2, C and D). Unlike CAR T cells, HIT T cells are not endowed with engineered costimulation support, which we and others have previously shown extends the persistence of engineered T cells (26, 28). When we coexpressed CD80 and 4–1BB ligand (4–1BBL) (29, 30) in CD70-HIT cells, HIT T cells achieved complete and durable tumor elimination in both PDX models (Fig. 2, B and C). Enumeration of T cells in vivo showed that CD70-CAR T cells accumulated in similar numbers at the kidney tumor site compared with CD70-HIT+CD80/4–1BBL T cells (Fig. 2E) but expressed more exhaustion markers (Fig. 2F) and failed to eliminate tumor (Fig. 2D). Coexpression of both CD80 and 4–1BBL costimulatory ligands, or 4–1BBL alone in CD70-CAR T cells (fig. S6), did not augment tumor clearance (fig. S7, A, B, D, and E).

To confirm that CD70-HIT+CD80/4–1BBL T cells mediated a CD70-specific response, we generated CRISPR-Cas9 CD70-ablated K5 (fig S8, A and B) and K7 (fig S9, A and B) PDX tumor cells. There was no killing of CD70-ablated K5 or K7 tumor by CD70-HIT T cells in prolonged in vitro cytotoxicity assays (figs. S8E and S9E) and in vivo (Fig. 2, G to I). Furthermore, mixing CD70 wild-type PDX tumor with 25% CD70-ablated tumor did not result in tumor eradication by CD70-HIT+CD80/4–1BBL T cells (Fig. 2, H and I), establishing that their in vivo efficacy was not due to bystander killing of CD70-negative tumor cells but required CD70 expression in tumor cells.

### *CD70* chromatin accessibility as a signature of low-level expression

**Fig. 3.**
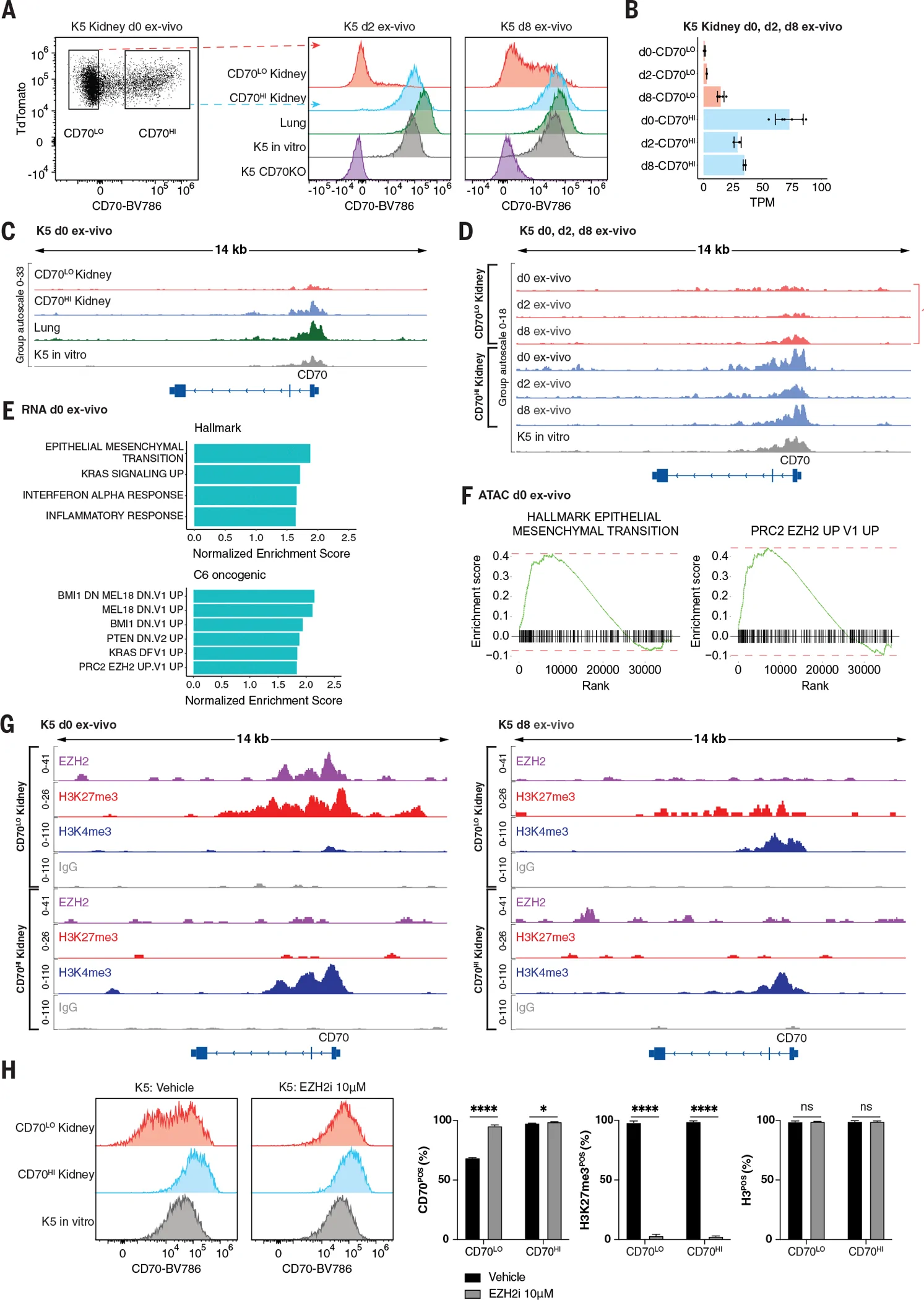
CD70 is epigenetically silenced by EZH2-mediated H3K27me3 to low levels. (**A**) CD70^LO^ and CD70^HI^ tumor cells from mice bearing K5 PDX tumor at both kidney and lung sites were isolated by fluorescence-activated cell sorting 15 days after tumor administration. Representative gating strategy to sort CD70^LO^ and CD70^HI^ tumor cells from kidney site, denoted as day 0 of isolation, “d0 ex-vivo.” Isolated CD70^LO^ and CD70^HI^ tumor cells from kidney site, and CD70^HI^ tumor cells from lung site, were then cultured for 8 days. Flow cytometric analysis of CD70 expression at day 2 in culture (“d2 ex-vivo”) and day 8 in culture (“d8 ex-vivo”). (**B**) CD70 transcript expression in CD70^LO^ and CD70^HI^ tumor cells from kidney site at d0, d2, and d8 ex vivo. *n* = 3 mice, mean ± SD. (**C**) Genome browser view of chromatin accessibility at the *CD70* gene in d0 ex vivo CD70^LO^ and CD70^HI^ tumor cells from kidney site, and CD70^HI^ tumor cells from lung site as determined by assay for transposase-accessible chromatin with sequencing (ATAC-seq). CD70HI versus CD70LO kidney tumor cells adjusted *P* value (*P*adj) = 7.45 × 10^−8^. Differential chromatin accessibility of *CD70* corresponds with differential CD70 expression. (**D**) Genome browser view of chromatin accessibility at the *CD70* gene in CD70^LO^ and CD70^HI^ tumor cells from kidney site at day 8 ex vivo as determined by ATAC-seq. 1.4× fold increase in differentially annotated peaks at *CD70* promoter of CD70^LO^ tumor cells from kidney site between d0 versus d8 ex vivo *P*adj = 0.08). (**E**) Normalized enrichment scores of significantly up-regulated gene sets in CD70^HI^ versus CD70^LO^ K5 and K7 tumor cells from kidney site at d0 ex vivo as determined by gene set enrichment analysis (GSEA) using MSigDB hallmark (top 4, *P*adj < 0.05) and C6 oncogenic gene sets (top 6, *P*adj < 0.05). (**F**) Enrichment plot for Hallmark EMT pathway, and C6 oncogenic PRC2 EZH2 UP V1 UP pathway (genes up-regulated upon EZH2 knockdown) in CD70^HI^ versus CD70^LO^ K5 and K7 tumor cells from kidney site at d0 ex vivo from GSEA analysis of ATAC data (*P*adj < 0.05). (**G**) Genome browser views of the *CD70* genomic region at d0 exvivo showing enrichment for EZH2 and repressive H3K27me3 histone modifications at the *CD70* locus in CD70^LO^ tumor cells from kidney, and activating H3K4me3 histone modifications at the *CD70* locus in CD70^HI^ tumor cells from the kidney, assessed by Cleavage Under Targets & Release Using Nuclease (CUT&RUN). As CD70 expression increased at d8 ex vivo, there was loss of H3K27me3 and a gain of H3K4me3 at the *CD70* locus in CD70^LO^ tumor cells from kidney site. (**H**) D0 exvivo CD70^LO^ and CD70^HI^ tumor cells from kidney site were treated with either dimethyl sulfoxide (vehicle) or tazemetostat (EZH2 inhibitor, EZH2i 10 μM) for 8 days. CD70, H3K27me3, and total histone H3 were analyzed by flow cytometry. *n* = 3 mice per condition, mean ± SD, ns, not significant; *P < 0.05, ****P < 0.0001, two-tailed *t* test.

To further investigate how CD70 expression is silenced to very low levels, we isolated CD70^LO^ and CD70^HI^ tumor cells from the kidney and lungs (all CD70^HI^) of mice bearing tumor at both sites and analyzed the CD70 transcriptional, protein, and chromatin accessibility profile upon ex-vivo isolation and subsequent culture (Fig. 3, A to D, and fig. S10). CD70 transcript levels in CD70^LO^ kidney tumor cells were at the limit of detection on day 0, minimal by day 2, and further increased by day 8 (Fig. 3B), in accordance with restoration of protein expression (Fig. 3A). Assaying for transposase-accessible chromatin using sequencing analysis uncovered significant differences between accessible chromatin regions for CD70 (Fig. 3C and fig. S10D). On day 0, CD70^HI^ tumor cells from the kidney and lungs exhibited open chromatin at the *CD70* promoter, whereas CD70^LO^ kidney tumor cells showed lower peak levels (Fig. 3C). By day 8, *CD70* chromatin accessibility in the CD70^LO^ kidney tumor cells increased 1.4-fold (Fig. 3D), concordantly with the increase in CD70 RNA and protein expression (Fig. 3, A and B).

An enrichment of gene sets associated with epithelial mesenchymal transition (EMT) and polycomb repressive complex 2 (PRC2) targets were identified in paired genome-wide transcriptional and epigenetic profiles of day 0 CD70^HI^ versus CD70^LO^ kidney tumor cells (Fig. 3, E and F). We examined the *CD70* genomic region for trimethylation of histone H3 on lysine 27 (H3K27me3), a repressive chromatin marker associated with PRC2 activity, and enhancer of zeste homolog 2 (EZH2), the catalytic subunit of PRC2. At day 0, both H3K27me3 and EZH2 were enriched at the *CD70* promoter in CD70^LO^ kidney tumor cells (Fig. 3G). By contrast, trimethylation of lysine 4 on histone H3 (H3K4me3), a chromatin mark associated with transcriptional activation, was enriched at the *CD70* promoter in CD70^HI^ kidney tumor cells (Fig. 3G). At day 8, CD70 re-expression in CD70^LO^ kidney tumor cells was associated with a loss of EZH2 binding and H3K27me3 at the *CD70* promoter and a gain of H3K4me3 (Fig. 3G). Further supporting these observations, treatment of day 0 CD70^LO^ kidney tumor cells with an EZH2 inhibitor led to more rapid CD70 re-expression than with vehicle (Fig. 3H). DNA methylation inhibition with azacytidine did not lead to CD70 re-expression (fig. S11F), and CpG islands in the *CD70* promoter were not methylated as determined by bisulfite sequencing (fig. S11D). These data establish that EZH2-mediated H3K27me3 epigenetic silencing of *CD70* is associated with very low but HIT-targetable CD70 expression levels in ccRCC tumor.

### Epigenetic signature of CD70 expression in ccRCC patient tumors

**Fig. 4.**
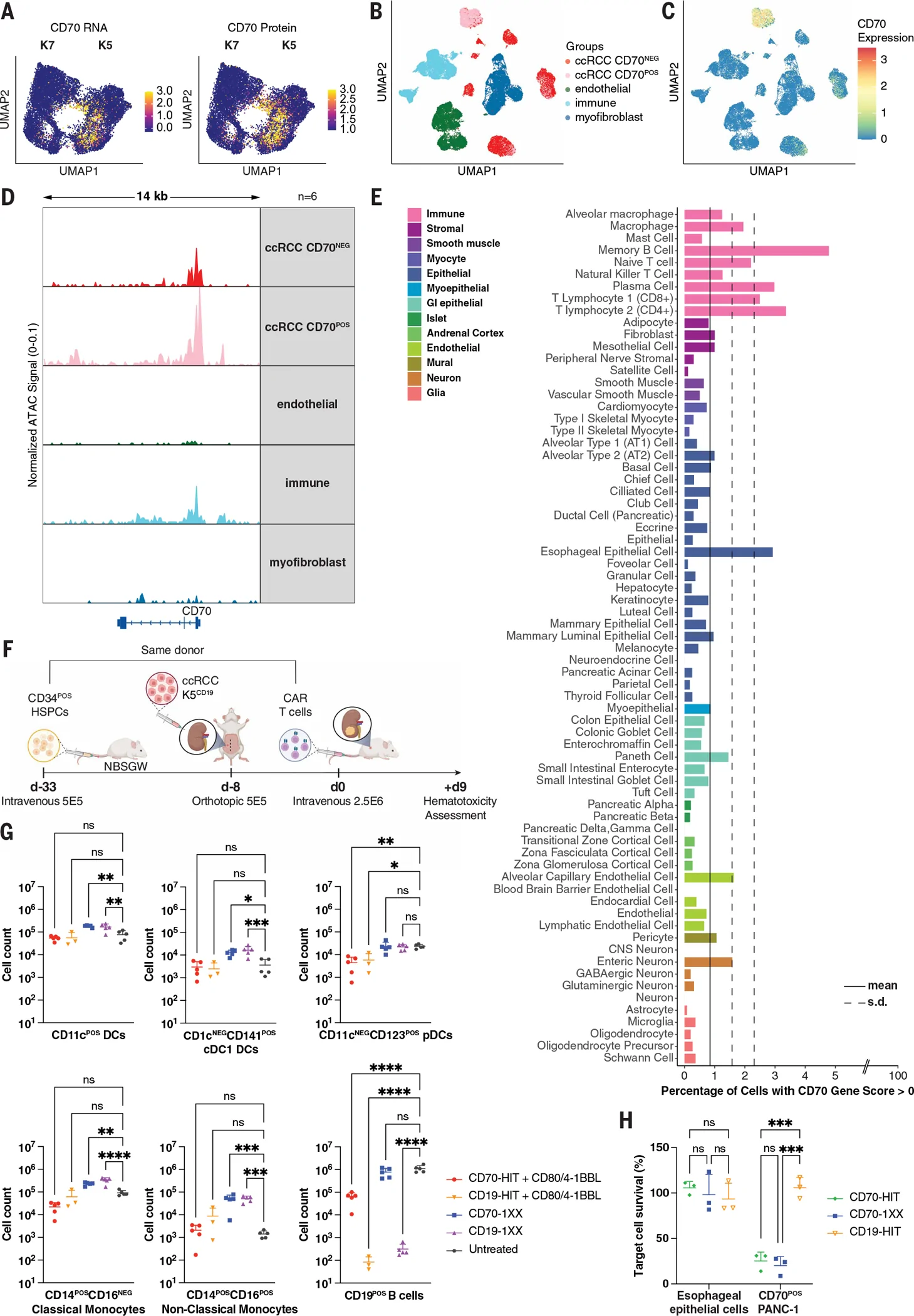
Epigenetic signature of *CD70* to predict low-level expression in ccRCC patient tumors and normal tissues. (**A**) Uniform manifold approximation and projection (UMAP) of single cells from K5 and K7 kidney tumor showing heterogeneous CD70 transcriptional and cell surface protein expression as detected by cellular indexing of transcriptomes and epitopes (CITE) sequencing. (**B** to **D**) ccRCC patient kidney tumors (*n* = 6) were collected for 10× Multiome [single-nucleus RNA sequencing (snRNA-seq) and single-nucleus chromatin accessibility (snATAC-seq)] to simultaneously profile the epigenome and transcriptome of a single cell. (B) UMAP plot showing the distribution of immune cells, cancer cells, endothelial cells, and myofibroblast across six patient primary samples. (C) UMAP plot showing CD70 gene expression across cell types. (D) Chromatin accessibility of the *CD70* gene in cells sorted in silico into CD70^POS^ and CD70^NEG^ tumor on the basis of transcript expression (*n* = 6 ccRCC tumors). (**E**) Percentage of cells with a *CD70* gene score > 0 based on chromatin accessibility to infer CD70 gene expression in 76 cell types found in normal tissues using a publicly available single-cell atlas of chromatin accessibility in the human genome (31). Solid black line represents the mean, and the dashed lines one and two SDs above the mean. (**F**) Schematic of humanized ccRCC mouse model to assess CD70-HIT T cell hematotoxicity in the context of antitumor response. NBSGW mice were humanized via injection of G-CSF–mobilized healthy donor–derived CD34^POS^ cells on d33, followed by orthotopic kidney injection of K5 ccRCC tumor constitutively expressing CD19, K5^CD19^ on d8, and either untreated or treated with 2.5 × 10^6^ CAR or HIT T cells on d0. CAR and HIT T cells were manufactured from T cells isolated from the same donor as CD34^POS^ cells. Antitumor response is shown in fig. S13. (**G**) The impact on normal human hematopoiesis after treatment with CD70–1XX CAR, CD19–1XX reference CAR, CD70-HIT+CD80/4–1BBL, and CD19-HIT+CD80/4–1BBL T cells was assessed by flow cytometric analysis of bone marrow on day 9 after T cell infusion (donor 18). Human bone marrow populations were quantified by flow cytometry, and representative gating is shown in fig. S13. *n* = 3 to 5 mice per group; mean ± SD; ns, not significant; *P < 0.05; **P < 0.01; ***P < 0.001; ****P < 0.0001; one-way ANOVA. (**H**) 18-hour in vitro cytotoxic activity of CD70-HIT, CD70–1XX CAR, CD19-HIT, or UTD T cells (donor 22) against adult human primary esophageal epithelial cells at a high effector-to-target ratio to elicit toxicity (E:T = 4:1). Target cell survival was quantified by flow cytometry. CD70-positive PANC-1 cell line used as an internal positive control. Survival is relative to cells co-cultured with UTD T cells. CD70 expression after 18 hours co-culture is shown in fig. S14E. *n* = 3; mean ± SD; ns, not significant; ***P < 0.001; one-way ANOVA. Representative of three different healthy donors.

To determine whether CD70 was epigenetically regulated in primary patient samples, we collected ccRCC kidney tumors (*n* = 6) at the time of nephrectomy (Table 2) and profiled individual cells simultaneously for chromatin accessibility and gene expression using the 10x Multiome platform. As seen in our kidney PDX models (Fig. 4A), CD70 expression was heterogeneous in patient tumors (Fig. 4, B and C). To ascertain whether apparent CD70-negative patient tumor retained differentially accessible chromatin at the *CD70* promoter, we sorted cells in silico into CD70^NEG^ and CD70^POS^ populations on the basis of transcript expression and analyzed chromatin accessibility. Differences in chromatin accessibility between CD70^NEG^ versus CD70^POS^ tumor recapitulated what was observed in our PDX models (Figs. 3C and 4D). By contrast, *CD70* was not accessible in normal tissues such as endothelial cells and myofibroblasts (Fig. 4D). We also observed enrichment of EMT (fig. S12E) and gene sets associated with *EZH2* (fig. S12F), similar to what we observed in our kidney PDX models. These data suggest that low-level CD70 expression, predicted by chromatin accessibility, occurs in apparently CD70-negative tumor cells of heterogeneous ccRCC patient tumors.

### Chromatin accessibility to predict on-target/off-tumor toxicity

Given that conventional methods of detection are at the limit of their sensitivity to detect low-level but HIT-targetable expression, we profiled *CD70* chromatin accessibility to predict HIT-sensitive expression in a single-cell ATAC atlas of 30 different normal human tissues comprising 222 different cell types (31) (Fig. 4E). Average gene scores for all cell types across tissues were generated using the ArchR R package (32). We considered the *CD70* locus to be accessible if the cell’s *CD70* gene score was greater than 0. *CD70* accessibility was limited to a few specific cell types, and within those cell types it was present in only a small percentage of cells (<5%). As expected, immune cell subsets presented the highest fraction of cells with accessible *CD70*, accounting for ≈5% of memory B cells, ≈3% of plasma cells, ≈2.5% of CD4^POS^ T lymphocytes, and ≈3% of CD8^POS^ T lymphocytes, consistent with flow cytometric CD70 profiling in peripheral blood mononuclear cells (PBMCs) (fig. S14).

To evaluate the off-tumor activity of CD70-HIT+CD80/4–1BBL T cells on normal hematopoiesis and immune cell subsets in the context of an antitumor response, we established a humanized ccRCC kidney model (Fig. 4F). NBSGW mice were humanized with granulocyte colony-stimulating factor (G-CSF)–mobilized adult healthy donor-derived CD34^POS^ hematopoietic stem and progenitor cells (HSPCs), followed by orthotopic kidney injection of K5 RCC tumor cells that had been modified to express CD19 (to activate the control CD19-targeted T cells). CAR and HIT T cells were manufactured from T cells isolated from the same donor as the CD34^POS^ cells. Humanized mice bearing K5^CD19^ tumor at the kidney site received either CD19–1XX, CD70–1XX, CD70-HIT+CD80/4–1BBL, or CD19-HIT+CD80/4–1BBL T cells at a dose of 2.5 × 10^6^ per mouse. A CD19-targeted CAR (CD19–1XX) served as a reference CAR, which has been used in the clinic (33) to benchmark expected on-target human CD19^POS^ B cell depletion and serve as a control for off-target toxicity against normal hematologic cells that do not express CD19. By day 7 after T cell infusion, there was rapid tumor clearance by CD70-HIT+CD80/4–1BBL, CD19–1XX, and CD19-HIT+CD80/4–1BBL T cells in humanized mice compared with CD70–1XX–treated mice (fig. S13A). This allowed us to assess the impact on normal hematologic cells at peak CAR and HIT T cell response. Bone marrow analysis on day 9 after T cell infusion detected CD11c^POS^ dendritic cells, the conventional CD1c^NEG^CD141^POS^ cDC1 type 1 dendritic cell subset, CD11c^NEG^CD123^POS^ plasmacytoid dendritic cells, classical CD14^POS^CD16^NEG^, and nonclassical CD14^POS^CD16^POS^ monocytes in similar amounts in CD70-HIT+ CD80/4–1BBL–treated mice and in CD19-HIT+CD80/4–1BBL–treated mice (Fig. 4G), establishing that dendritic cells and monocytes were not depleted. CD70-HIT+CD80/4–1BBL–treated mice showed a reduced B cell compartment, albeit not a B cell aplasia as seen in mice treated with CD19–1XX or CD19-HIT+CD80/4–1BBL CAR T cells (Fig. 4G). Thus, at a time of comparable CD70-CAR and CD70-HIT T cell accumulation (fig. S13B), CD70-HIT+CD80/4–1BBL T cells demonstrated greater antitumor efficacy, without inflicting greater myeloid depletion than CD70-CARs or greater B cell depletion than CD19-CARs.

Given that CD70 is absent on resting immune cells but can be induced upon activation, we tested the cytotoxicity of CD70-CAR or CD70-HIT T cells against nonactivated and activated T cells, B cells, and dendritic cells. Consistent with our findings in the humanized mouse model, and further validating our predicted gene scores, we observed that nonactivated immune cells are CD70-negative and are not lysed by CD70-HIT or CD70-CAR T cells (figs. S14 to S16). The activated immune cells that induced CD70 were similarly eliminated by CD70-HIT and CD70-CAR T cells (figs. S15 and S16).

Esophageal epithelial cells were the only nonimmune cell type to show a *CD70* gene score >0 in more than ≈1% of cells, approaching 3%. CD70 expression was detected in 1 to 3% of normal esophageal cells by flow cytometry (fig. S14E). This small subpopulation was lysed by CD70-HIT and CD70-CAR T cells in cytotoxicity assays (fig. S14E); however, the remainder of primary human esophageal epithelial cells were not killed (Fig. 4H), reducing concerns for on-target CD70-HIT activity against esophageal epithelium. These findings support using *CD70* chromatin accessibility to screen normal tissues for potential on-target and off-tumor toxicity of sensitive CD70-targeted immunotherapy.

### CD70-HIT T cells eliminate pancreatic and ovarian tumors

**Fig. 5.**
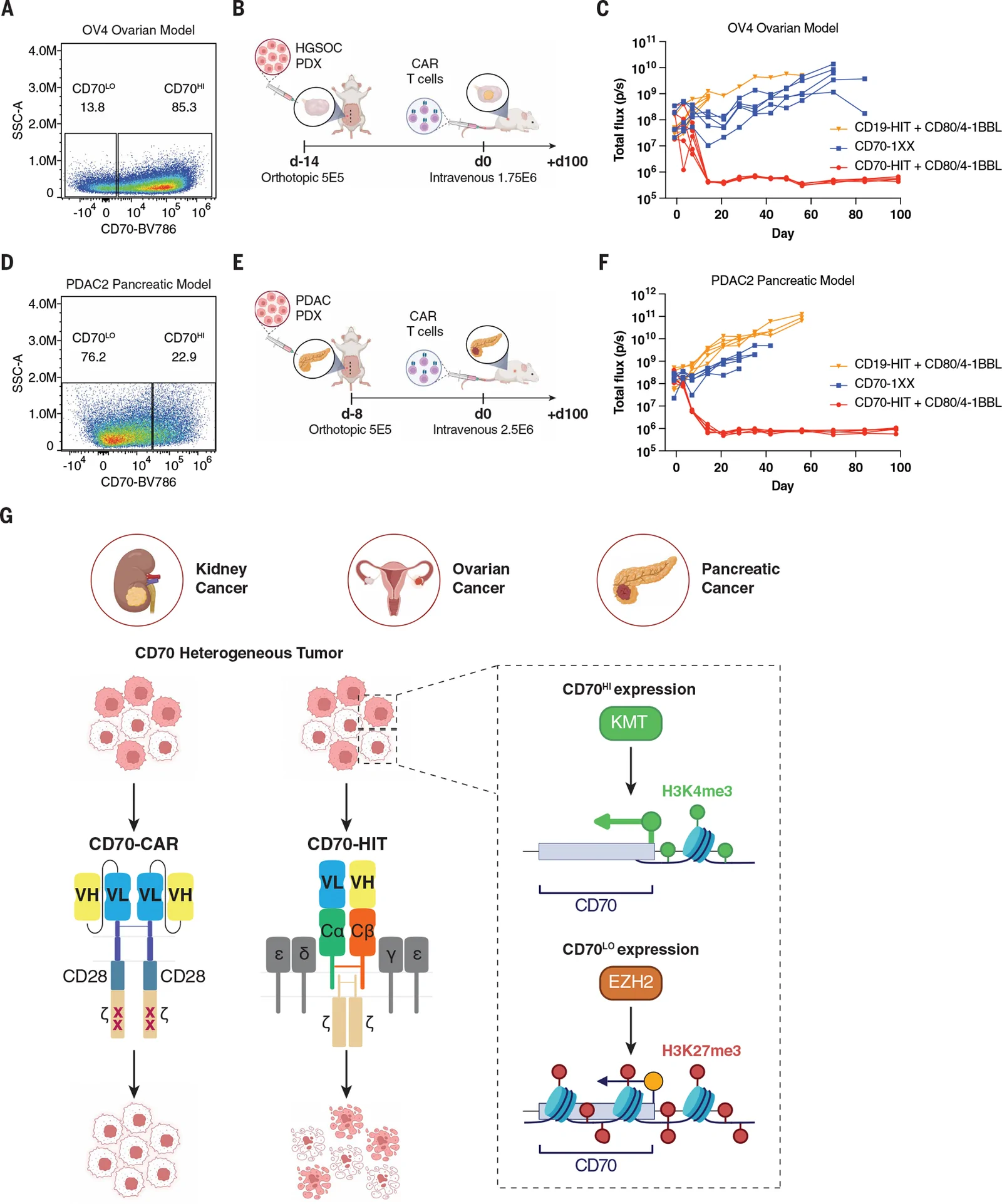
CD70-HIT T cells eliminate CD70-heterogeneous ovarian and pancreatic tumors. (**A**) Representative flow cytometric analysis of CD70 expression on OV4 high-grade serous ovarian carcinoma (HGSOC) PDX tumor at ovarian site. (**B**) Diagram depicting in vivo CAR T treatment of HGSOC ovarian model. OV4 ovarian PDX tumor is administered orthotopically (5 × 10^5^ cells per mouse) into the ovary of NSG mice with tumor growth predominantly in the ovary. CAR T cell timing, dose, and route of administration depicted. (**C**) Tumor burden (total flux) in mice bearing OV4 PDX tumor at ovarian site and treated with 1.75 × 10^6^ CD70–1XX CAR, CD70-HIT+CD80/4–1BBL or CD19-HIT+CD80/4–1BBL T cells (donor 19). *n* = 5 mice per group. Representative of two independent in vivo experiments (with two different donors). (**D**) Representative flow cytometric analysis of CD70 expression on PDAC2 pancreatic ductal adenocarcinoma (PDAC) PDX tumor at pancreatic site. (**E**) Diagram depicting in vivo CAR T treatment of PDAC PDX pancreatic model. PDAC2 PDX tumor is injected orthotopically (5 × 10^5^ cells per mouse) into the pancreas of NSG mice with tumor growth localized in the pancreas. CAR T cell timing, dose, and route of administration depicted. (**F**) Tumor burden (total flux) in mice bearing PDAC2 PDX tumor in the pancreas and treated with 2.5 × 10^6^ CD70–1XX CAR, CD70-HIT+CD80/4–1BBL or CD19-HIT+CD80/4–1BBL T cells (donor 26). *n* = 5 mice per group. Representative of three independent in vivo experiments (with three different donors). (**G**) CD70-HIT T cells effectively eliminate CD70-heterogeneous ccRCC, ovarian, and pancreatic tumors where prototypic CD70-CAR T cells fail. This is based on the finding that CD70 expression in heterogeneous tumors is not binary but epigenetically silenced by EZH2-H3K27me3 to very low levels, which elude conventional CD70-CAR T cells but can be detected by more sensitive CD70-HIT T cell receptors. KMT, lysine methyltransferase.

Other solid tumors, including ovarian and pancreatic cancer, are reported to express CD70 heterogeneously (19). To determine whether CD70 low-level expression was also retained in CD70-heterogeneous tumors of other cancer types, we tested CD70-HIT T cell efficacy in ovarian and pancreatic cancer models. In OV4, a high-grade serous ovarian carcinoma (HGSOC) PDX, and SK-OV3, an ovarian cancer cell line, CD70 expression was heterogeneous at the ovarian site (Fig. 5A and fig. S17F). CD70-HIT+CD80/4–1BBL T cells eradicated tumor completely in both ovarian models compared with CD70-CAR T cells (Fig. 5, B and C, and fig. S17H). We next evaluated whether CD70 was epigenetically regulated in the HGSOC model. Profiling of *CD70* chromatin accessibility in CD70^LO^ and CD70^HI^ tumor cells at the ovarian site revealed differential chromatin accessibility (fig. S18F). There was an enrichment of gene sets associated with EMT and PRC2 targets in the epigenetic profiles of CD70^HI^ versus CD70^LO^ ovarian tumor cells (fig. S18E). Similar to what we observed in the ccRCC kidney models, we found an enrichment of EZH2 and repressive H3K27me3 at the *CD70* promoter in CD70^LO^ ovarian cancer tumor cells and an enrichment of H3K4me3 at the *CD70* promoter in CD70^HI^ ovarian cancer tumor cells (fig. S18G). Consistent with our findings in the ccRCC models, we again found the CpG islands in the *CD70* promoter to be unmethylated (fig. S18H).

In PDAC2, a *KRAS* mutated pancreatic ductal adenocarcinoma PDX, and PANC-1, a pancreatic cancer cell line, CD70 expression was also heterogeneous at the pancreatic site (Fig. 5D and fig. S19F). CD70-HIT+CD80/4–1BBL T cells eradicated tumor completely in both pancreatic models compared with CD70-CAR T cells (Fig. 5, E and F, and fig. S19, G to I). Higher CD70 expression in the PANC-1 model (fig. S19F) corresponded with improved CD70-CAR T cell performance but still resulted in incomplete tumor elimination by a conventional CAR (fig. S19, H and I). Of note, PDAC2 pancreatic PDX was heterogeneous for CD70 expression in vitro (fig. S20A) with ~20% of tumor cells positive, which is in a similar range reported for primary pancreatic cancer samples (13). Exposure to an EZH2 inhibitor (fig. S20, G and H) but not azacytidine (fig. S20I) up-regulated CD70 expression, unmasking epigenetically down-regulated expression and further suggesting that EZH2-mediated H3K27me3 repression of *CD70* expression occurs in some CD70-heterogeneous tumors. These findings support that CD70-HIT+CD80/4–1BBL T cells may be effective against a broad range of solid tumors that display partial CD70 positivity by conventional staining techniques.

## Discussion

We demonstrate that we can overcome the challenge of heterogeneous CD70 expression across several solid tumor models of ccRCC, ovarian cancer, and pancreatic cancer by using a highly sensitive CAR targeting CD70. This strategy is based on the finding that CD70 expression in heterogeneous PDX tumors is not binary but exhibits a spectrum from high to very low—appearing negative by conventional detection methods. Thus, tumors with heterogeneous CD70 expression may express CD70 in all tumor cells, though at levels too low for conventional CAR T cells to be effective. Such tumors can be eliminated by highly sensitive antigen recognition as conferred by HLA-independent T cell receptors, establishing CD70 as a potential pan-cancer target in a broad range of tumors.

Our results show that the epigenetic silencing of *CD70* within heterogeneous tumors leads to strong but incomplete gene silencing driving pseudo-heterogeneous expression. This epigenetic silencing is mediated by EZH2-H3K27me3 in ccRCC, HGSOC, and PDAC tumors. Thus, the epigenetic modulation of an immunotherapeutic target within heterogeneous solid tumors can undermine tumor responsiveness to prototypic CAR T cells but is overcome by more sensitive antigen recognition. Polycomb group proteins and H3K27me3 are associated with transcriptional quiescence, but previous reports have shown that H3K27me3-repressed genes can still be transcribed at low levels (34–36). Studies investigating CD70 expression in hematological malignancies have reported its induction using hypomethylating agents (37, 38). This contrasts with our findings in solid tumors, which show that CD70 can be up-regulated by EZH2 inhibition but not azacytidine, suggesting that distinct mechanisms modulate CD70 expression across tumor types.

Given that conventional methods for measuring CD70 RNA and protein expression are at their limit of sensitivity to detect very low CD70 expression, our findings suggest that *CD70* chromatin accessibility may be used as a proxy for low-level expression and predicting responsiveness to a CD70-HIT T cell approach. Profiling *CD70* chromatin accessibility in a larger cohort of ccRCC patient primary tumors revealed the same patterns of differential chromatin accessibility as seen in our ccRCC PDX models, suggesting that CD70 is epigenetically regulated in apparent CD70-negative patient tumors, further supporting the therapeutic potential of CD70-HIT T cells. Using a very stringent gene score cut-off to define an accessible *CD70* locus in adult normal tissues, we found most cell types to be inaccessible aside from immune cells. We observed high concordance in normal tissues between percentage of cells with accessible *CD70*, percentage of CD70-positive cells on flow cytometry, and percentage of CD70-positive cells eradicated in killing assays, reinforcing the reliability of these measurements. Furthermore, our cytotoxicity assays against normal tissues and findings in the humanized model did not reveal differential toxicities between CD70-HIT T cell and CD70-CAR T cell therapies. Results from CD70-CAR T clinical trials have so far shown a favorable safety profile, with cytopenias commonly associated with CAR T cell therapy and lymphodepletion but without toxicity to gastrointestinal or other normal tissues (23–25, 39). Whether an open chromatin state will prove to be a reliable predictor of response to therapy remains to be established.

Several studies targeting CD70 in solid tumor models have yielded encouraging results (25, 40, 41), but these have not translated to expected patient outcomes. Unlike commonly used tumor cell lines, our preclinical ccRCC models display real-world tumor heterogeneity of CD70 expression and mirror the modest response rates observed with CD70-CAR T cells in clinical trials. Indeed, two phase I clinical trials evaluating allogeneic CD70-CAR T cells in patients with metastatic renal cell carcinoma so far have reported objective response rates of 6 to 21% (23–25, 42). We similarly only obtained partial responses for our orthotopic kidney PDX models treated with CD70-CAR T cells. In COBALT-RCC, allogeneic CD70-CAR T cells were short-lived, and undetectable beyond day +28, with correspondingly poor response rates; persistent CD70 tumoral expression observed on day +42 biopsies thus reflects a time point when there is no CAR T cell activity present (25). Updated clinical results from the TRAVERSE trial, in which CD70-CAR T cells persist and infiltrate tumor, demonstrate a clear relationship between baseline tumoral CD70 level and therapeutic response (42), with higher antigen levels resulting in greater tumor reduction (23). In our lung model of ccRCC, a frequent site of metastasis in ccRCC patients, CD70 expression was high and tumor was cleared by a single infusion of CD70-CAR T cells, corroborating clinical case reports showing CAR T cell efficacy at this site (23, 24). Although additional tumor microenvironment factors may influence efficacy, the preclinical and clinical data support that antigen level is a key determinant of CD70-CAR T cell therapeutic response.

We further noted an enrichment of an EMT signature in CD70-positive tumor cells in both ccRCC PDX models and patient kidney tumor samples. An EMT signature was also noted in our HGSOC PDX model. A link between higher CD70 expression and metastases has been observed in osteosarcoma and breast cancer (43–45); CD70 expression has also been associated with hematogenous metastasis and EMT in cancer of the pancreas (14), as well as EMT-associated epidermal growth factor receptor (EGFR) tyrosine kinase inhibitor resistance in non–small cell lung cancer (46, 47). Altogether, these reports and our findings suggest that CD70 expression is induced or increased in tumor cells undergoing EMT cell state transition.

The vast majority of CARs in clinical development act independently of the TCR complex. The HIT receptor is one of the few synthetic receptors acting via the CD3 complex (27). Here, we demonstrate the efficacy of the HIT receptor against heterogeneous solid tumors, where it enables detection and targeting of previously unrecognized, epigenetically driven CD70 pseudo-heterogeneity. Although the HIT receptor mediates rapid tumor clearance, it requires additional costimulatory support, extending prior observations in a leukemia model (26). These findings redefine what constitutes a targetable cancer and offer a potential mechanism independent of microenvironmental suppression to account for the limited efficacy of current CD70-CAR T cell therapies. It remains to be determined whether all CD3-dependent CAR structures are as sensitive to low antigen expression in solid tumors as we report here for the HIT configuration.

Twenty or more solid tumor types express CD70 heterogeneously (19). Our works opens up the possibility that tumors comprising only a fraction of CD70-positive tumor cells may express CD70 in more tumor cells than identified by conventional staining methods, and may therefore be targeted by a sensitive immunotherapy such as CD70-HIT T cells. Our findings position CD70 as a pan-cancer target and provide a model for uncovering additional stealth targets amenable to sensitive immunotherapeutic approaches in the face of apparent tumor antigen heterogeneity.

## Supplementary Material

Supplemental Data

Repdoducibility Checklist

science.org/doi/10.1126/science.adv7378

Materials and Methods; Figs. S1 to S20; Tables S1 and S2; References (48–72); MDAR Reproducibility Checklist

## Figures and Tables

**Table 1.** Clinical features of clear cell renal cell carcinoma (RCC) models (K5 and K7). IMDC, International Metastatic Renal Cell Carcinoma Database Consortium; NGS, next-generation sequencing. Asterisk denotes truncating mutation.

	K5		K7	
**Age at diagnosis (years)**	67		43	
**Sex**	Male		Male	
**Histology**	Clear cell RCC		Clear cell RCC with sarcomatoid differentiation	
**Nephrectomy status**	Yes		No	
**IMDC risk at diagnosis**	Intermediate-Poor		Intermediate-Poor	
**Stage at initial diagnosis**	Metastatic		Metastatic	
**Sites of disease**	Adrenal, bone, brain, lung, pleura		Adrenal, bone, brain, lung, lymph node	
**Treatment history**	Sunitinib, axitinib, bevacizumab, everolimus		Temsirolimus, axitinib, everolimus	
**MSK-IMPACT NGS of tissue site**	Kidney		Bone, lymph node	
	Gene	Protein Change	Gene	Protein Change
	*PTEN*	L345Qfs*2	*TSC1*	Y185*
	*PTEN*	K163Nfs*2	*NF2*	K130Rfs*44
	*VHL*	L158P	*SETD2*	Q2362*
	*BAP1*	X194_splice	*PBRM1*	*1579fs*
	*PBRM1*	R146P	*COP1*	W219L
	*EPHA7*	S631F	*NSD1*	G596S
	*HNF1A*	T544A	*RPTOR*	Q673P
	*BCL2*	S105P		
	*NCOA3*	E788Q		

**Table 2.** Clinical features of clear cell renal cell carcinoma (RCC) patient tumors profiled by 10× Multiome. N/A, not applicable.

Multiome cohort	Age	Sex	Histology	Stage at diagnosis	IMDC risk at diagnosis	Multiome tissue	Prior treatment history
**1**	52	M	Clear cell RCC with sarcomatoid and rhabdoid features	IV	Intermediate-Poor	Kidney	None
**2**	36	F	Clear cell RCC with rhabdoid features	IV	Intermediate-Poor	Kidney	Lenvatinib + Pembrolizumab
**3**	74	M	Clear cell RCC with sarcomatoid features	IV	Intermediate-Poor	Kidney	Axitinib + Pembrolizumab + lung radiotherapy
**4**	77	M	Clear cell RCC	III	N/A	Kidney	Ipilimumab + Nivolumab
**5**	65	M	Clear cell RCC	III	N/A	Kidney	None
**6**	67	M	Clear cell RCC	III	N/A	Kidney	None

## Data Availability

The bulk RNA-seq data of ccRCC PDX tumor cells isolated from the kidney and lung sites have been deposited in the Gene Expression Omnibus (GEO) of the National Center for Biotechnology Information (NCBI) (accession no.: GSE306609). The bulk RNA-seq data of HGSOC PDX tumor cells isolated from the ovary have been deposited in the GEO of the NCBI (accession no.: GSE306267). The bulk ATAC-seq data and the CUT&RUN data of ccRCC PDX tumor cells isolated from the kidney, and HGSOC PDX tumor cells isolated from the ovary, have been deposited in the GEO of the NCBI (accession nos.: GSE311096, GSE311098). The CITE-seq data of ccRCC PDX tumor cells isolated from the kidney have been deposited in the GEO of the NCBI (accession no.: GSE306608). The 10x Multiome data of ccRCC patient tumors have been deposited in the GEO of the NCBI (accession no.: GSE306275). Reagents are available from the corresponding author upon reasonable request. Plasmids will be distributed via Addgene.
