# Supplementary material for: Sensitive CAR T cells redefine targetable CD70 expression in solid tumors
Source: Science. Author manuscript; Available in PMC 2026 Aug 20. (PMC13491402; doi:10.1126/science.adv7378)
Supplement: Supplementary file 2 [file NIHMS2197918-supplement-Supplemental_Data.pdf]

Science
AAAS

Supplementary Materials for

Sensitive CAR T cells redefine targetable CD70 expression in solid tumors

Sophie A. Hanina *et al*.

Corresponding author: Michel Sadelain, mws2188@cumc.columbia.edu

Science **391**, 896 (2026)
DOI: 10.1126/science.adv7378

The PDF file includes:

Materials and Methods
Figs. S1 to S20
Tables S1 and S2
References

Other Supplementary Material for this manuscript includes the following:

MDAR Reproducibility Checklist

Materials and Methods

Ethical approval

All animal experiments were performed under a protocol approved by the Memorial Sloan Kettering Cancer Center Institutional Animal Care and Use Committee (protocol 04-10-024). Human biospecimen collection and use were approved by the Memorial Sloan Kettering Institutional Review Board (IRB 12-237).

Guide RNA (gRNA)

The *TRAC* and *CD70* loci were disrupted in CAR and HIT T cells by CRISPR-Cas9 editing as previously described (*26, 48*). The sequence targeted by *TRAC* gRNA is located upstream of the transmembrane domain of the TCRα. *TRAC* gRNA target sequence: 5′-CAGGGTTCTGGATATCTGT. *TRAC* and *CD70* gRNAs were ordered from Synthego with 2′-O-methyl 3′ phosphorothioate modifications in the first and last 3 nucleotides. Guide RNA was resuspended in TE buffer at 80 μM.

To generate CRISPR-Cas9 *CD70*-edited cell lines and PDX lines, three *CD70* gRNAs were used: 5’- AGCGCUGGAUGCACACCACG; 5’-AGCCCGCAGGACGCACCCAU; 5’-CGCCGCGGCGAUGCCGGAGG.

Cas9 protein and RNP formation

Cas9 protein (40 μM) was obtained from QB3-Berkeley Macrolab core facility. For TCR^{KO} and $CD70^{KO}$ T cell editing, RNP were prepared by mixing *TRAC* gRNA and *CD70* gRNA with Cas9 protein at 1:1:1 molar ratios, and incubated at 37 °C for 15 min, and immediately used for T cell editing experiments.

AAV and gene targeting

The CD19-HIT and CD70-HIT receptor AAV construct was made as described previously (*26*). The CD70-HIT receptor, which is targeted to the *TRAC* locus (*48*) and driven by the endogenous *TCRα* promoter, uses the same heavy and light chain as the CD70-CAR, thus producing an HLA-independent TCR recognizing the same epitope but signaling via the CD3 complex (Fig. 2A). We cloned the pAAV-*TRAC*-CD70-1XX P2A EGFRt by replacing the 1928z sequence of our previously described pAAV-*TRAC*-1928z construct (*48*) with the CD70-1XX sequence. Recombinant AAV6 viruses (with AAV2 ITRs) were produced by Signagen, with titers expressed as GC/ml.

Gamma retroviral vector constructs, production and transduction

Plasmids encoding the SFGγ retroviral vector (*49*) were used to clone CD19-1XX, CD70-1XX, and the bicistronic construct expressing human CD80 and 4-1BBL genes (sequence provided in Table S1). CD80 and 4-1BBL SFGγ retroviral vectors have been previously described (*26*). Vesicular stomatitis virus glycoprotein (VSV-G) pseudotyped retroviral supernatants derived from transduced gpg29 fibroblasts (H29) were used to construct stable retroviral-producing cell lines (*50*).

Lentiviral vector construction and production

EF1α-CD70 lentiviral plasmid was generated by cloning the EF1α promoter, mCerulean fluorescent protein and *CD70* sequences into a SIN lentiviral plasmid (pLM backbone) (*51*).

PGK-CD19 lentiviral plasmid was generated by cloning the PGK promoter and *CD19* sequences into a SIN lentiviral plasmid (pLM backbone). VSV-G pseudotyped lentiviral supernatants derived from transfected 293T cells were used to transduce K5 and K7 to generate stable PDX lines constitutively expressing mCerulean-CD70, or -CD19. Transduction was performed in 6-well plates containing 4 µg/ml polybrene.

Human T cell isolation, gene targeting and transduction

Buffy coats from healthy volunteer donors were obtained from the New York Blood Center. Peripheral blood mononuclear cells (PBMCs) were isolated by density gradient centrifugation using Lymphocyte Separation Medium (Corning). T cells were isolated using a Pan T Cell Isolation Kit (Miltenyi Biotec) and activated with CTS Dynabeads CD3/CD28 activation beads (ThermoFisher) at a 1:1 bead:T cell ratio in TheraPeak X-VIVO-15 Serum-free Hematopoietic Cell Medium (Lonza), supplemented with 5% human serum (GeminiBio), 50 U/ml penicillin and 50 µg/ml streptomycin (Gibco), 5 ng/ml human recombinant interleukin-7 (Miltenyi Biotec) and 5 ng/ml human recombinant interleukin-15 (Miltenyi Biotec) at a density of 1×10^6 cells per ml. Cells were cultured in a 37 °C incubator with 5% CO2.

Forty-eight hours after initiating T cell activation, CD3/CD28 beads were magnetically removed. T cells were resuspended in P3 buffer (Lonza) and were transfected by electrotransfer with Cas9 ribonucleoprotein (RNP) and *TRAC* gRNA and *CD70* gRNA using a 4D-Nucleofector device (Lonza). Following electroporation, 66.7% viability was assumed. For AAV transduction, T cells were resuspended in X-VIVO 15 without human serum, 50 U/ml penicillin, 50 µg/ml streptomycin, interleukin-7, and interleukin-15 in G-Rex plates. Recombinant AAV6 donor vector was added to the T cells 30 min to 1 hour after electroporation, at a multiplicity of infection of 2×10^4 per viable T cell.

For SFG γ-retroviral vector transduction, T cells were plated onto non-tissue culture-treated six-well plates pre-coated with retronectin (Takara) 24 hours after electroporation and transduced with retroviral supernatants. Plates were centrifuged for 1 hour at 300 g.

Cell cultures were replenished with additional T cell medium as needed to maintain a density of 1×10^6 to 1.5×10^6 cells per ml, with fresh interleukin-7 and interleukin-15 replenished every forty-eight hours. Transduction efficiencies were determined 3 days later by flow cytometry, and T cells were used for in vitro experiments or injected into tumor-bearing mice. All reported doses reflect the number of CAR^{POS} or HIT^{POS} T cells. In all in vitro and in vivo experiments comparing CAR versus HIT T cells, the CAR or HIT construct was targeted to the *TRAC* locus. For in vitro and in vivo experiments using only CAR T cells, T cells were γ-retrovirally transduced.

Cell lines, patient-derived xenograft cell lines and culture conditions

PANC-1 pancreatic epithelioid carcinoma (ATCC, Cat # CRL-1469) and SK-OV3 ovarian adenocarcinoma cells (ATCC, Cat # HTB-77) were transduced with firefly luciferase (FFLuc)-green fluorescent protein (GFP) retroviral vector and subsequently sorted for GFP-positive cells. PANC-1 cell line was maintained in DMEM supplemented with 10% fetal bovine serum (Neuromics), 50 U/ml penicillin and 50 µg/ml streptomycin (Gibco). SK-OV3 cell line was maintained in McCoy's 5a Medium Modified (ATCC, Cat # 30-2007) supplemented with 10% FBS (Neuromics), and 50U/ml penicillin and 50 µg/ml streptomycin (Gibco). NALM6 B-ALL

leukemia cells (ATCC, Cat # CRL-3273) expressing FFluc-GFP were cultured as previously described (*52*). K5, K7, PDAC2 and OV4 are PDX lines generated in-house and lentivirally transduced with FFLuc-Tdtomato (Addgene, 48688). K5, K7 and PDAC2 were maintained in RPMI-1640 (Corning) supplemented with 10% fetal bovine serum (Neuromics), 50 U/ml penicillin and 50 μg/ml streptomycin (Gibco). OV4 was maintained in RPMI-1640 (Corning) supplemented with 20% fetal bovine serum (Neuromics), 50 U/ml penicillin and 50 μg/ml streptomycin (Gibco). Human primary esophageal epithelial cells were obtained from Cell Biologics (H-6046, Lot# F141616.JS p5) and maintained in Complete Human Epithelial Cell Medium with Supplement Kit (H6621).

<u>Kidney, ovarian and pancreatic cancer mouse models</u>

For the orthotopic ccRCC kidney model (model outline described in Fig. 1C), 5×10^5 FFLuc-Tdtomato K5 or K7 ccRCC cells were injected into the renal subcapsule (d-8) of male NOD.Cg-Prkdc scid IL2rg tm1Wjl/SzJ (NSG, Jackson Laboratory) mice, aged 6-8 weeks. Eight days later (d0), CAR or HIT T cells were administered via tail vein injection at the indicated doses.

For the ccRCC lung model (model outline described in fig. S2B), 7.5×10^5 FFLuc-Tdtomato K5 or K7 ccRCC cells were administered via tail vein injection (d-5) into male NSG mice aged 6-8 weeks. Five days later, CAR T cells were administered via tail vein injection at the indicated doses.

For the orthotopic ovarian model (model outline described in Fig. 5B), 5×10^5 FFLuc-Tdtomato OV4 or FFLuc-GFP SK-OV3 cells were injected into the ovary (d-14) of female NSG mice aged 6-8 weeks. Fourteen days later (d0), CAR or HIT T cells were administered via tail vein injection at the indicated doses.

For the pancreatic ductal adenocarcinoma PDX model (model outline described in Fig. 5E), 5×10^5 FFLuc-Tdtomato PDAC2 cells were injected into the pancreas (d-8) of male NSG mice aged 6-8 weeks. Eight days later (d0), CAR or HIT T cells were administered via tail vein injection at the indicated doses. For the orthotopic pancreatic cell line model (fig. S19G), 5×10^5 FFLuc-GFP PANC-1 were injected into the pancreas (d-14) of male NSG mice aged 6-8 weeks. Fourteen days later (d0), CAR or HIT T cells were administered via tail vein injection at the indicated doses.

Tumor burden was measured by bioluminescence imaging using the IVIS Imaging System (PerkinElmer) with the Living Image V4.8 software (PerkinElmer) for the acquisition of imaging datasets. Survival was monitored for a minimum of 100 days after T cell injection.

<u>Humanized ccRCC xenograft model</u>

Humanized mice injected orthotopically with ccRCC were used to evaluate the impact of in vivo activated CAR or HIT T cells on normal human hematopoietic cells (model outline described in Fig. 4F). CAR or HIT T cells were generated from cryopreserved T cells originating from the same donor as HSPCs, using the T cell transduction protocol as described above. 2 days after HSPC thawing (d-33), in vitro expanded HSPCs were injected via tail vein into female 6-week old NOD.Cg-$Kit^{W\text{-}41J}$ *Tyr* $^{+}$ $Prkdc^{scid}$ $Il2rg^{tm1Wjl}$/ThomJ (NBSGW, Jackson Laboratory) mice (5×10^5 per mouse). 25 days after HSPC injection (d-8), FFLuc-Tdtomato $K5^{CD19}$ cells (5×10^5 per mouse) were orthotopically injected into the renal subcapsule. 33 days after HSPC injection (d0), *TRAC*-targeted CAR T cells (19-1XX or CD70-1XX) or HIT T cells (CD70-HIT+CD80/4-1BBL

or CD19-HIT+CD80/4-1BBL), were injected via tail vein, 2.5×10^6 per mouse. Antitumor efficacy was monitored via bioluminescence imaging. On day 9 after T cell infusion, bone marrow from untreated, CAR-treated, and HIT-treated mice was collected and analyzed by flow cytometry.

Cytotoxicity assays

The cytotoxicity of CD70-HIT and CD70-1XX CAR T cells was determined by standard firefly luciferase (FFL)-based assay. FFL-expressing K5, K7, PANC-1, PDAC2, SK-OV3 or NALM6 served as target cells. Cells were co-cultured in triplicates at the indicated effector (E) to target (T) (E:T) ratios using black-walled 96-well plates with 1×10^4 solid tumor cell lines or 5×10^4 NALM6 in a final volume of 150 µl per well in T cell medium. After 18 hours, 100 µl luciferase substrate (Bright-Glo, Promega) was added to each well. Emitted light was detected using a luminescence plate reader (Agilent BioTek S1L), and lysis was calculated using the formula 100 x (1-(RLUsample)/(RLUtarget alone), where RLU is relative light units.

Flow-based cytotoxicity assays of non-activated and activated immune cell subsets

For flow cytometry-based cytotoxicity assays, CAR or HIT T cells were labeled with CellTracker Green Dye (Invitrogen, C7025) and co-cultured in triplicate with 5×10^4 autologous PBMCs, 1×10^5 autologous T cells, or 1×10^5 autologous dendritic cells at the indicated E:T ratios using 96-well U-bottom plates in a final volume of 150 µl per well in T cell medium and analyzed by flow cytometry 18 hours later. Autologous T cells were isolated and activated as described above. Cells were maintained for an additional 5-7 days before use in cytotoxicity assays. Paired non-activated T cells were cryopreserved at the time of isolation, and thawed 24 hours prior to assay.

Dendritic cell activation and flow cytometry gating strategy

Dendritic cells were isolated from G-CSF-mobilized healthy adult donor-derived leukopaks and PBMCs using the human Blood Dendritic Cell Isolation Kit II (Miltenyi Biotec) or the EasySep™ Human Pan-DC Pre-Enrichment Kit (STEMCELL Technologies) and cultured in TheraPeak X-VIVO-15 Serum-free Hematopoietic Cell Medium (Lonza), supplemented with 5% human serum (GeminiBio), 50 U/ml penicillin and 50 µg/ml streptomycin (Gibco), 10 ng/ml human recombinant interleukin-3 (Miltenyi Biotec) and 10 ng/ml human recombinant GM-CSF (Miltenyi Biotec) unless otherwise indicated.

Dendritic cells were activated for 22 hours with 1 µg/mL CD40L (Miltenyi Biotec), 0.5 µg/mL R848 (STEMCELL Technologies), 25 µg/mL poly(I:C) (Miltenyi Biotec), 10 ng/ml human recombinant interleukin-3 (Miltenyi Biotec) and 10 ng/ml human recombinant GM-CSF (Miltenyi Biotec). B cells present in the pre-enriched dendritic cell population were also responsive to these activation conditions, and were analyzed by flow cytometry.

The following gating strategies were performed to identify relevant dendritic cell populations:
Plasmacytoid dendritic cells—CellTracker Green^{NEG} CD3^{NEG} CD19^{NEG} CD56^{NEG} CD14^{NEG} CD16^{NEG} HLA-DR^{POS} CD11c^{NEG} CD123^{POS}, CD303^{POS}
Conventional dendritic cells—CellTracker Green^{NEG} CD3^{NEG} CD19^{NEG} CD56^{NEG} CD14^{NEG} CD16^{NEG} HLA-DR^{POS} CD11c^{POS} CD123^{NEG}.

Tissue dissociations

Kidney tumors collected from patients undergoing surgery, and tumor from PDX models were digested into single cell suspensions using the Tumor Dissociation Kit, human (Miltenyi Biotec) according to the manufacturer's instructions. Red cell blood lysis was performed using ACK buffer (Lonza).

Flow cytometry

Flow cytometry antibodies used for cell surface phenotyping and intracellular staining are provided in Table S2. FcR Blocking Reagent (mouse, Miltenyi Biotec; human, Miltenyi Biotec) was used to block Fc receptors. For intracellular staining, cells were fixed and permeabilized using Transcription Factor Buffer set (BD). DAPI was used to exclude dead cells (BD). Countbright Absolute Counting beads (Invitrogen) were used to determine the number of cells according to the manufacturer's protocol. Flow cytometric data were acquired on a spectral flow cytometer (Cytek Aurora, 5-laser configuration) and analyzed using FlowJo v10.8.1. Cell sorting was performed on a BD FACSAria cell sorter.

Immunohistochemistry

Formalin fixed paraffin embedded (FFPE) tissue or cell pellets were sectioned at 5 µM thick. Immunohistochemical staining for CD70 was performed using a commercially available antibody, clone 301731 (MAB2738, R&D Systems) at 1:100 dilution (2.6 µg/mL). Staining was performed on the Leica Bond-3 auto staining system (Leica, Deer Park, IL), using heat-based antigen retrieval, a high pH buffer solution (AR9640; Leica, Bond Epitope Retrieval Solution 2, 30 min), 30 min primary incubation time, and a polymer detection system (DS9800; Leica, Bond Polymer Refine Detection).

Immunofluorescence, microscopy & image analysis

Automated immunofluorescence (IF) with the mitochondria primary antibody was conducted using Leica Bond BX staining system. Paraffin-embedded tissues were sectioned at 5 µM and baked at 58°C for 1 hour. Slides were loaded in Leica Bond and IF staining was performed as follows. Samples were dewaxed and pretreated with EDTA-based epitope retrieval ER2 solution (Leica, AR9640) for 20 min at 100°C. The mouse anti-mitochondria primary antibody (ms, 0.1 µg/ml, Millipore MAB1273) was incubated with the sample for 1 hour at RT. A rabbit anti-mouse (Abcam, ab133469) secondary antibody was then incubated for 8 min before the application of the Leica Bond Polymer anti-rabbit HRP secondary antibody (included in Polymer Refine Detection Kit, Leica, DS9800) for 8 min at RT. Signals were then visualized by incubation with Tyramide-Alexa fluorophore A488 (Invitrogen, B40953) for 10 min at RT. After the run was finished, slides were washed in PBS and the manual staining of CD70 was performed. Briefly, before applying anti-CD70 antibody, the slides were again pretreated with EDTA-based epitope retrieval ER2 solution (Leica, AR9640) for 20 min at 100°C in Leica Bond to remove the anti-mitochondria and the binding secondary antibody. Different concentrations of CD70 antibody (ms, 1:50, 1:100, 1:200, and 1:400, R&D Systems, MAB2738) were incubated overnight at 4°C. Slides were washed in PBS and then incubated with anti-mouse-STAR 635 secondary antibody (1:100, Abberior, ST635-1001) for 1 hour at RT. After washing in PBS, the slides were incubated with 5 µg/ml 4',6-diamidino-2-phenylindole (DAPI) (Sigma Aldrich) for 5 min, and mounted in ProLong Diamond Antifade mountant (Invitrogen, 2836823) with Carl Zeiss High performance coverslips #1.5H (170 µM+/-5 µM) (474030-9000-000). Slides were kept overnight at -20°C before imaging.

Confocal microscopy was performed in the Rockefeller University's Bio-Imaging Resource Center, RRID:SCR_017791. Images were acquired using Abberior IMSPECTOR LIGHTBOX software (v2023.26.18333-g490e35373e) on a Facility Line spectral confocal and STED module (Abberior Instruments) attached to an Olympus IX83 microscope stand. A UPLXAPO 100X/1.45 NA oil (RI:1.518) objective lens (Olympus), 405 nm (CW), 488 nm (pulsed), 640 nm (pulsed) laser lines were used to capture fluorescence from DAPI, Alexa Fluor 488, and Abberior STAR 635P labelled tissue sections into separate avalanche photodiode (APD) detectors. Excitation laser power, line accumulation, pixel dwell time, pixel and z-step sizes, were all optimized on the knockout samples to set the background signal level and kept consistent for all other samples. The confocal pinhole size was kept at 1AU. Scanned images were saved as [.obf] format image files, and deconvolved in Huygens Professional (v24.04). Mean fluorescence intensity was measured on individual cells from 3-8 large area images using the free hand selection tool in Fiji (ImageJ) (*53*). Corrected total cell fluorescence (CTCF) (*54*) was calculated using the formula: CTCF = Integrated Density – (Area of selected cell X Mean fluorescence of background readings). From the ROI manager in Fiji, roi selections corresponding to [DAPI^{POS}mitochrondria^{NEG}] cells in the CD70 channel were selected as areas for measuring background intensity.

Western blotting

Proteins of whole cell lysates in 1× Laemmli buffer (Bio-Rad) were separated by SDS-PAGE (Bio-Rad) and transferred onto polyvinylidene difluoride membranes (Bio-Rad). The following primary antibodies were used: Tri-Methyl-Histone H3 (Lys27) (Cell Signaling Technology, C36B11), and Histone H3 (Cell Signaling Technology, 1B1B2). Goat anti-rabbit IgG, horseradish peroxidase (HRP)-linked antibody (Cell Signaling Technology, 7074S) and Goat anti-mouse IgG, HRP-linked antibody (Invitrogen, A10551) were used. For developing, the Immobilon Western Chemiluminescent HRP Substrate (EMD Millipore) and the ChemiDoc MP Imaging System with Image Lab Software (Bio-Rad) were used.

Cite-Seq and data analysis

Prior to FACS sorting, cell suspensions were Fc Receptor blocked with TruStain FcX (BioLegend) for 10 min at 4°C prior to being stained with Total-Seq-A CD70 antibody (Ki24, BD; custom conjugated by BioLegend) for 30 min on ice. The single-cell RNA-seq was performed on Chromium instrument (10x genomics) following the user guide manual for 3′ v3.1 protocol. Specific extracellular protein epitopes were characterized (using Antibody Derived Tag-ADT) following previously published protocol (*55*).

CITE-seq data analysis was conducted using Seurat (*56*) (version 5.1.0). Cells were filtered to ensure they had between 800 and 8000 total features, between 1000 and 60000 total counts, and less than 10% mitochondrial RNA content. Antibody capture data were normalized using a centered log-ratio (CLR) transformation across cells. RNA data were log-normalized with a scale factor of 10000 across features. The top 2000 variable genes were identified for downstream analysis. Principal component analysis (PCA) was performed, and the first 20 principal components were used to conduct clustering and generate Uniform Manifold Approximation and Projection (UMAP) (*57*) visualizations.

ATAC-Sequencing and data analysis

ATAC was performed on sorted and viably frozen cells as previously described (*58*) using the Tagment DNA TDE1 Enzyme (Illumina, #20034198). Sequencing libraries were purified with

AMPure XP magnetic beads (Beckman Coulter, A63881), and quantified using a Qubit fluorometer (ThermoFisher Scientific) and a TapeStation (Agilent) and sequenced on a NovaSeq 6000. Raw sequencing reads were trimmed and filtered for quality (Q>15) and adapter content using version 0.4.5 of TrimGalore and running version 1.15 of cutadapt and version 0.11.5 of FastQC. Version 2.3.4.1 of bowtie2 (*59*) (http://bowtie-bio.sourceforge.net/bowtie2/index.shtml) employed to align reads to human assembly hg38 and alignments were deduplicated using MarkDuplicates in Picard Tools v2.16.0. ATAC-seq peaks for each replicate were identified using MACS2 (*60*) and filtered for blacklisted regions (https://mitra.stanford.edu/kundaje/akundaje/release/blacklists/hg38-human/hg38.blacklist.bed.gz). Reproducibility of peaks between pairs of replicates from each condition was evaluated using the irreproducible discovery rate (IDR) (*61*) procedure. After excluding peaks that were not reproducible across replicates, a peak atlas was constructed by aggregating reproducible peaks (IDR < 0.05) from ex-vivo samples of the kidney mouse model. Peaks that overlap with each other in the atlas were merged using the reduce function from GenomicRanges v1.46.1. A read count matrix was constructed by counting ATAC-seq reads for peaks using featureCounts from Rsubread (*62*) package v2.8.2. Differential accessibility of the peaks was computed using DESeq2 (*63*) v1.34.0. Gene set enrichment analysis (GSEA) was performed using fGSEA (*64*). For GSEA, peaks were first assigned to nearest genes using ChIPseeker (*65*) v1.30.3. Next, peak with the greatest magnitude of log fold change within 50kb of transcription start site for each gene was used for fGSEA.

CUT&RUN

CUT&RUN was performed on sorted and viably frozen cells using the CUTANA™ ChIC/CUT&RUN Kit (Epicypher, 14-1048) following the manufacturer's instructions. The following antibodies were used: rabbit anti-H3K4me3 (Epicypher, 13-0041); rabbit anti-H3K27me3 (Cell Signaling Technology, #9733); rabbit anti-EZH2 (Epicypher, 13-2026); negative control rabbit IgG (Epicypher, 13-0042). After quantification of the recovered DNA fragments, libraries were prepared using the ThruPLEX®DNA-Seq kit (Takara, R400676) following the manufacturer's instructions, purified with AMPure XP magnetic beads (Beckman Coulter, A63881), and quantified using a Qubit fluorometer (ThermoFisher Scientific) and a TapeStation (Agilent), and sequenced on a NovaSeq 6000.

Transcriptome Sequencing and analysis

After RiboGreen quantification and quality control by Agilent BioAnalyzer, 125-500 ng of total RNA with RIN values of 8.3-10 underwent polyA selection and TruSeq library preparation according to instructions provided by Illumina (TruSeq Stranded mRNA LT Kit, RS-122-2102), with 8 cycles of PCR. Samples were barcoded and run on NovaSeq 6000 or X in a PE100 run, using the NovaSeq 6000 S4 or X 10B Reagent Kit (200 Cycles) (Illumina). An average of 84 million paired reads was generated per sample. Ribosomal reads represented 1.7-24% of the total reads generated and the percent of mRNA bases averaged 79%. Raw RNA-seq reads were trimmed and filtered using fastp v0.23.4. Reads were aligned to the reference human genome hg38 using STAR (*66*) aligner v2.7.10. Transcript annotations from Gencode (*67*) (Release 44 GRCh38.p14) were used for the genomic location of transcripts. Transcript abundances were quantified using the alignment-based mode of Salmon (*68*) v0.13.1. Transcript abundances were then aggregated into gene-level counts using tximport v1.22.0 package in R (4.1.2) with gene models from Gencode v44. Differential gene expression analysis between samples was performed using DESeq2 v1.34.0. False discovery rate (FDR) for results of differential

expression analysis was computed using Benjamini-Hochberg procedure for multiple hypothesis correction. Gene set enrichment analysis (GSEA) was performed using fGSEA v1.20.0 using log fold changes from differential gene expression analysis as gene ranks. Enrichment for pathways in hallmark gene sets, chemical and genetic perturbations, and oncogenic gene sets were evaluated.

10x Multiome Sequencing and data analysis

Single Cell Multiome ATAC + Gene Expression was performed with 10x genomics system using Chromium Next GEM Single Cell Multiome Reagent Kit A (1000282) and ATAC Kit A (1000280) following Chromium Next GEM Single Cell Multiome ATAC + Gene Expression Reagent Kits User Guide and demonstrated protocol - Nuclei Isolation for Single Cell Multiome ATAC + Gene Expression Sequencing. Briefly, cells were lysed for 4 min and resuspended in Diluted Nuclei Buffer (10x Genomics, PN- 2000207). Lysis efficiency and nuclei concentration were evaluated on Countess II automatic cell counter by trypan blue staining. Nuclei were loaded in the transposition reaction, targeting recovery of 10,000 nuclei after encapsulation. After transposition reaction nuclei were encapsulated and barcoded. Next-generation sequencing libraries were constructed following User Guide, which were sequenced on an Illumina NovaSeq X+ system.

FASTQ files from the 10x Multiome were aligned to GRCh38 (CellRanger human reference genome GRCh38-2020-A-2.0.0) using CellRanger Arc 2.0.2 with default parameters. After alignment, ambient RNA reads were removed in all samples using SoupX(*69*) v.1.6.2 with default parameters, and the filtered read count matrices were used for downstream analyses. For quality control, cells in each sample were filtered based on the number of RNA transcripts (>1,000 and <60,000), the number of detected genes (>500 and <7,000), and the fraction of mitochondrial transcripts (<30%). Cells were additionally filtered based on the number of ATAC fragments (>1,000 and <100,000) and transcription start site enrichment score (>4). Genes detected in <2 cells were discarded. Arrow files for ATAC fragments were created using ArchR v1.0.2 (*32*). Doublets were identified and removed using ArchR with default parameters. After clustering the snRNA-seq data and snATAC-seq data (described in 'Dimensionality reduction, cell clustering, and visualization'), contaminant clusters with low quality or high doublet scores were removed. In total, 41,766 cells remained for downstream analyses.

Dimensionality reduction, cell clustering, and visualization

RNA count matrices after ambient RNA correction from six patient samples were first combined into a single matrix. The count matrix was then library-size normalized, log-transformed, and centered and scaled using Seurat (*70*) v4.4.0. Principal component analysis (PCA) was performed on the scaled data (number of principal components = 50). A nearest-neighbor graph was constructed using the first 30 principal components (PCs) with 30 nearest neighbors. Clustering was performed using Louvain algorithm (resolution = 1) on the shared nearest-neighbor graph. For the ATAC analysis, Latent Semantic Indexing (LSI) was performed on 50,000 top variable tiles (500bp genomic bins) identified after five iterations of 'IterativeLSI' by ArchR. Tiles from chrM, and chrY were not included in the analysis. Cells were clustered using Louvain algorithm (resolution 1). 41,766 cells remaining after excluding contaminant clusters were clustered again on the shared nearest-neighbor graph from Seurat (resolution = 1.4). Cell type for each cluster was assigned based on expression of marker genes. ccRCC cells were further labeled as CD70^{NEG} if expression of CD70 is 0, and CD70^{POS} otherwise. Cell clustering

was visualized using Uniform manifold approximation and projection (UMAP), computed from the nearest neighbor graph from Seurat.

Differential gene expression test

Differentially expressed genes (DEGs) between groups of cells were identified with MAST (*71*) using FindMarkers and FindAllMarkers functions from Seurat. For all comparisons, MAST was run on the log-normalized expression values of all genes. Specific comparisons are described in the results. DEGs with fold change > 1.5 and adjusted *P*-value < 0.01 were considered significant. Ribosomal and mitochondrial genes were excluded from visualization. Where stated, top DEGs were selected in each group based on the magnitude of fold change.

Gene set enrichment analysis

Gene set enrichment analysis for the multiome data was performed using fGSEA with log fold changes from MAST differential expression test as gene ranks. Enrichment for pathways in hallmark gene sets, chemical and genetic perturbations, and oncogenic gene sets were evaluated, and pathways with false discovery rate < 0.05 were considered significant.

Human atlas single-nuclei ATAC-seq analysis

The metadata for the human single-nuclei ATAC-seq (snATAC-seq) atlas (*31*) was manually curated. Fragment file analysis was conducted using ArchR (version 1.02), with the reference genome set to hg38. Quality control measures included setting a minimum transcription start site (TSS) enrichment score of 7 and requiring a minimum of 1000 mapped ATAC-seq fragments per cell for inclusion. Gene scores were computed for each arrow file and subsequently summarized by grouping cells based on tissue and cell type, followed by normalization according to the number of cells. Cell types representing less than 0.01% of the population in any given tissue were excluded from downstream analysis. Specifically, gene scores for the CD70-201 transcript were utilized in further analyses.

CD70 promoter methylation assessment by methylation-specific PCR

Tumor-derived genomic DNA (200-350 ng) was bisulfite converted using the EZ DNA Methylation-Lightning Kit (Zymo Research). The methylation status of two CpG islands in the *CD70* promoter was assessed by methylation-specific PCR. The following primers were used for amplification of the *CD70* methylated sequence: forward primer, 5'-TCGGTAGCGTTTCGC-3' and reverse primer, 5'- AACAACCCGAACCCTC-3'; forward primer, 5'-GTGCGGCGTAGGTTT-3' and reverse primer, 5'-TAAATACACACCACGAAACA-3'. The following primers were used for amplification of the *CD70* unmethylated sequence: forward primer, 5'-TCGGTAGCGTTTCGC-3' and reverse primer, 5'-AACAACCCGAACCCTC-3'; forward primer, 5'-GGTTTGGTGATTTGTTTCG-3', and reverse primer, 5'-TAAATACACACCACGAAACA-3'. Optimization was done with known positive controls, Universal Unmethylated DNA (Millipore) and CpG Methylated DNA (ThermoFisher). After PicoGreen quantification, 0.4-3.7 ng bisulfite converted gDNA was combined with locus-specific primers, FAM- and HEX-labeled probes, Hind III, 0.5 M betaine, and digital PCR Supermix for probes (no dUTP). All reactions were performed on a QX200 ddPCR system (Bio-Rad, 1864001). Reactions were partitioned into a median of ~23,000 droplets per well using the QX200 droplet generator. Emulsified PCRs were run on a 96-well thermal cycler using cycling conditions identified during the optimization step (95°C 10'; 40 cycles of 94°C 30' and 56°C 1'; 98°C 10'; 4°C hold). Plates were read and analyzed with the QuantaSoft sotware to assess the

number of droplets positive for methylated sites, unmethylated sites, both, or neither. Methylation Frequency = 100 * Methylated/(Methylated + Unmethylated).

Table S1. Construct sequences.

Name	Sequence
CD70 scFv	CAGGTGCAGCTGGTGGAGTCTGGGGGAGGC GTGGTCCAGCCTGGGAGGTCCCTGAGACTCT CCTGTGCAGCCTCTGGATTTACCTTCAGTAGC TATATTATGCACTGGGTCCGCCAGGCTCCAG GCAAGGGGCTGGAGTGGGTGGCAGTTATATC ATATGATGGAAGAAACAAATACTACGCAGACT CCGTGAAGGGCCGATTCACCATCTCCAGAGA CAATTCCAAGAACACGCTGTATCTGCAAATGA ACAGCCTGAGAGCTGAGGACACGGCTGTGTA TTACTGTGCGAGAGATACGGATGGCTACGATT TTGACTACTGGGGCCAGGGAACCCTGGTCAC CGTCTCCTCAGGTGGAGGTGGATCAGGTGGA GGTGGATCTGGTGGAGGTGGATCTGAAATTG TGTTGACACAGTCTCCAGCCACCCTGTCTTTG TCTCCAGGGGAAAGAGCCACCCTCTCCTGCA GGGCCAGTCAGAGTGTTAGCAGCTACTTAGC CTGGTACCAACAGAAACCTGGCCAGGCTCCC AGGCTCCTCATCTATGATGCATCCAACAGGGC CACTGGCATCCCAGCCAGGTTCAGTGGCAGT GGGTCTGGGACAGACTTCACTCTCACCATCA GCAGCCTAGAGCCTGAAGATTTTGCAGTTTAT TACTGTCAGCAGCGTACCAACTGGCCGCTCA CTTTCGGCGGAGGGACCAAGGTGGAGATCAA A
CD80 P2A 4-1BBL	ATGGGCCACACACGGAGGCAGGGAACATCAC CATCCAAGTGTCCATACCTCAATTTCTTTCAG CTCTTGGTGCTGGCTGGTCTTTCTCACTTCTG TTCAGGTGTTATCCACGTGACCAAGGAAGTGA AAGAAGTGGCAACGCTGTCCTGTGGTCACAA TGTTTCTGTTGAAGAGCTGGCACAAACTCGCA TCTACTGGCAAAAGGAGAAGAAAATGGTGCT GACTATGATGTCTGGGGACATGAATATATGGC CCGAGTACAAGAACCGGACCATCTTTGATATC ACTAATAACCTCTCCATTGTGATCCTGGCTCT GCGCCCATCTGACGAGGGCACATACGAGTGT GTTGTTCTGAAGTATGAAAAAGACGCTTTCAA GCGGGAACACCTGGCTGAAGTGACGTTATCA GTCAAAGCTGACTTCCCTACACCTAGTATATC TGACTTTGAAATTCCAACTTCTAATATTAGAAG GATAATTTGCTCAACCTCTGGAGGTTTTCCAG AGCCTCACCTCTCCTGGTTGGAAAATGGAGAA GAATTAAATGCCATCAACACAACAGTTTCCCA AGATCCTGAAACTGAGCTCTATGCTGTTAGCA GCAAACTGGATTTCAATATGACAACCAACCAC AGCTTCATGTGTCTCATCAAGTATGGACATTT AAGAGTGAATCAGACCTTCAACTGGAATACAA CCAAGCAAGAGCATTTTCCTGATAACCTGCTC CCATCCTGGGCCATTACCTTAATCTCAGTAAA TGGAATTTTTGTGATATGCTGCCTGACCTACT

	GCTTTGCCCCAAGATGCAGAGAGAGAAGGAG<br>GAATGAGAGATTGAGAAGGGAAAGTGTACGC<br>CCTGTAGGATCTGGAGCAACAAACTTCTCACT<br>ACTCAAACAAGCAGGTGACGTGGAGGAGAAT<br>CCCGGCCCCATGGAATACGCCTCTGACGCTT<br>CACTGGACCCCGAAGCCCCGTGGCCTCCCGC<br>GCCCCGCGCTCGCGCCTGCCGCGTACTGCCT<br>TGGGCCCTGGTCGCGGGGCTGCTGCTGCTG<br>CTGCTGCTCGCTGCCGCCTGCGCCGTCTTCC<br>TCGCCTGCCCCTGGGCCGTGTCCGGGGCTC<br>GCGCCTCGCCCGGCTCCGCGGCCAGCCCGA<br>GACTCCGCGAGGGTCCCGAGCTTTCGCCCGA<br>CGATCCCGCCGGCCTCTTGGACCTGCGGCAG<br>GGCATGTTTGCGCAGCTGGTGGCCCAAAATG<br>TTCTGCTGATCGATGGGCCCCTGAGCTGGTA<br>CAGTGACCCAGGCCTGGCAGGCGTGTCCCTG<br>ACGGGGGGCCTGAGCTACAAAGAGGACACGA<br>AGGAGCTGGTGGTGGCCAAGGCTGGAGTCTA<br>CTATGTCTTCTTTCAACTAGAGCTGCGGCGCG<br>TGGTGGCCGGCGAGGGCTCAGGCTCCGTTTC<br>ACTTGCGCTGCACCTGCAGCCACTGCGCTCT<br>GCTGCTGGGGCCGCCGCCCTGGCTTTGACC<br>GTGGACCTGCCACCCGCCTCCTCCGAGGCTC<br>GGAACTCGGCCTTCGGTTTCCAGGGCCGCTT<br>GCTGCACCTGAGTGCCGGCCAGCGCCTGGG<br>CGTCCATCTTCACACTGAGGCCAGGGCACGC<br>CATGCCTGGCAGCTTACCCAGGGCGCCACAG<br>TCTTGGGACTCTTCCGGGTGACCCCCGAAAT<br>CCCAGCCGGACTCCCTTCACCGAGGTCGGAA<br>TAA

Table S2. List of antibodies and clones.

Antibody Name	Vendor	Clone	Cat #
human CD3 BV510	BD Biosciences	HIT3a	564713
human CD3 RB705	BD Biosciences	UCHT1	570237
human CD3 RY775	BD Biosciences	UCHT1	571377
human CD4 BUV395	BD Biosciences	L200	564107
human CD8 BUV805	BD Biosciences	SK1	612889
human CD45 PerCP	BioLegend	HI30	304026
human CD45 APC/Fire 810	BioLegend	HI30	304076
human CD45 Pacific Orange	ThermoFisher	HI30	MHCD4530
mouse CD45 AF488	BioLegend	30-F11	103121
human CD19 Pe/Fire 700	BioLegend	SJ25C1	363049
human CD19 BUV563	BD Biosciences	SJ25C1	612916
human CD56 BV711	BioLegend	HCD56	318336
human CD56 RB670	BD Biosciences	B159	571925
human CD16 Super Bright 436	ThermoFisher	CB16	62-0166-42
human CD16 Super Bright 600	ThermoFisher	CB16	62-0168-42
human CD16 BUV805	ThermoFisher	CB16	368-0168-42
human CD14 AF647	BioLegend	63D3	367128
human CD14 Spark Blue 550	BioLegend	63D3	367148
human CD14 RB613	BD Biosciences	63D3	758932
human CD15 BV650	BioLegend	W6D3	323034
human HLA-DR BUV395	BD	L243	568648

human CD123 BV785	BioLegend	6H6	306032
human CD11c RB613	BD	B-ly6	571109
human CD11c R718	BD	B-ly6	566932
human CD1c RY703	BD	F10/21A3	770574
human CD1c RB780	BD	F10/21A3	569337
human CD141 BV650	BD	1A4	569392
human CD303 BV421	BioLegend	5F4	354212
human CD70 PE	BD Biosciences	Ki-24	555835
human CD70 BUV737	BD Biosciences	Ki-24	612856
human CD70 BV786	BD Biosciences	Ki-24	565338
human PD-1 BV711	BioLegend	EH12.2H7	329928
human TIM-3 BV785	BioLegend	F38-2E2	345032
human LAG-3 BV650	BioLegend	11C3C65	369316
human CD137L (4-1BBL) APC/Fire 750	BioLegend	5F4	311514
human EGFR AF647	BioLegend	AY13	352918
human CD271 (LNGFR) PE	BD Biosciences	C40-1457	557196
human CD324 (E-Cadherin) AF647	BioLegend	67A4	324112

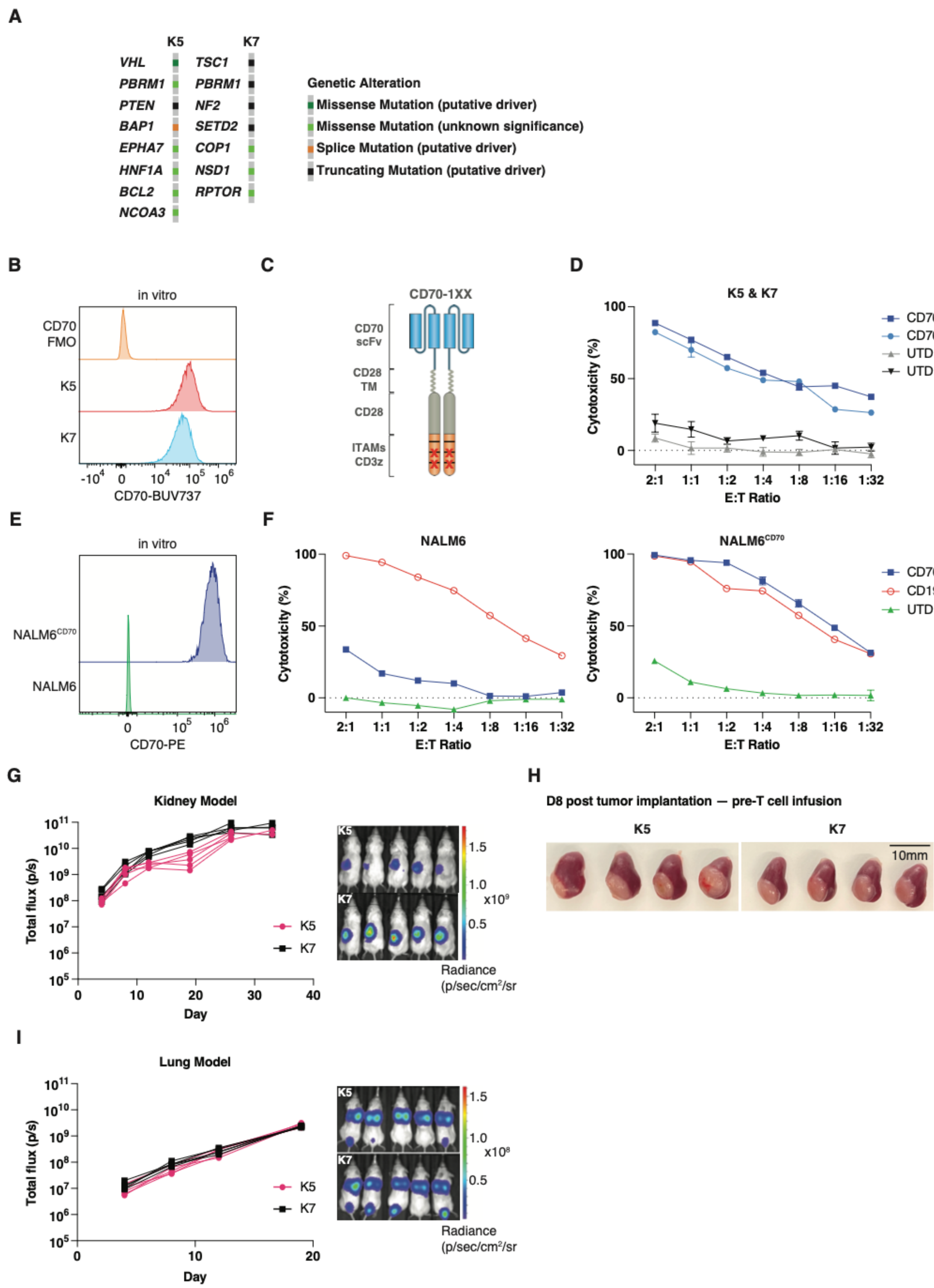

fig. S1. CD70-CAR T cell in vitro killing of K5 and K7 clear cell renal cell carcinoma (ccRCC) PDX (related to Fig. 1).
(**A**) OncoPrint showing actionable genetic mutations in the patient tumors from which K5 and K7 were established, profiled by next generation DNA sequencing. (**B**) Flow cytometric analysis showing in vitro expression of CD70 on K5 and K7. (**C**) Diagram depicting CD70-1XX CAR with a CD28 hinge and transmembrane domain, a CD28 costimulatory domain, and an

attenuated ζ chain signaling domain, which is mutated (indicated by red cross) in two out of three distal immunoreceptor tyrosine-based activation motifs (ITAMs), termed 1XX (*72*). (**D**) Cytotoxic activity of CD70-1XX CAR or untransduced (UTD) T cells (donor 1) against firefly luciferase expressing K5 and K7 target cells, which were incubated at the indicated E:T ratios, as determined by 18-hour bioluminescence assay. $n = 3$, mean ± SEM. Representative of three different donors. (**E**) Flow cytometric analysis showing in vitro expression of CD70 on NALM6 (which is negative for CD70) and NALM6 constitutively overexpressing CD70 ($NALM6^{CD70}$). (**F**) Comparison of cytotoxic activity of CD70-1XX CAR T cells against firefly luciferase-expressing NALM6 and $NALM6^{CD70}$ as determined by18-hour bioluminescence assay. $n = 3$, mean ± SEM. Donor 2. (**G**) Growth kinetics of K5 and K7 orthotopically implanted into the kidney, measured by bioluminescence imaging (BLI). Total flux is shown. (**H**) Size of K5 and K7 kidney tumors 8 days after implantation into the renal subcapsule prior to T cell infusion. (**I**) Growth kinetics of K5 and K7 injected intravenously to establish a lung model, measured by BLI (total flux is shown).

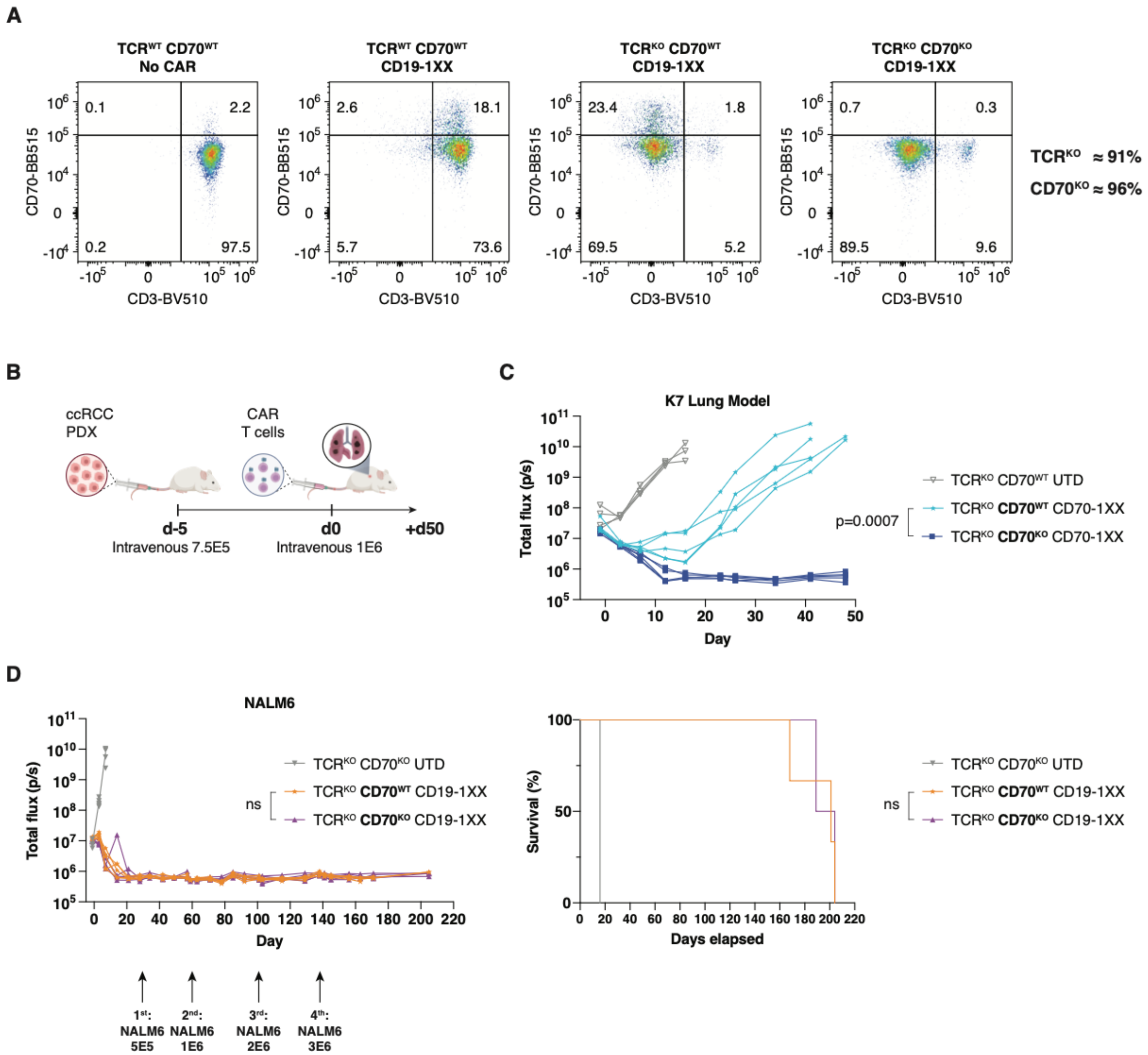

fig. S2. CRISPR-Cas9 editing of *TRAC* and *CD70* loci in CAR T cells.
(**A**) Representative flow cytometric plots of CD3 and CD70 expression 4 days after CRISPR-Cas9 editing of either the *TRAC* and/or *CD70* loci in CD19-1XX transduced T cells. CD70 expression is induced in CAR-transduced T cells. TCR^{KO} efficiency ≈ 91% by flow cytometry. $CD70^{KO}$ efficiency ≈ 96% by flow cytometry, and 93% indel frequency on ICE (Inference of CRISPR Edits, Synthego) analysis. For all experiments, *TRAC* and *CD70* loci were ablated in infused T cells unless otherwise stated. (**B**) Diagram depicting in vivo CAR T treatment of ccRCC lung model. K5 or K7 ccRCC tumor is administered intravenously (7.5×10^5 per mouse) into NSG mice with tumor growth predominantly in the lungs. CAR T cell timing, dose and route of administration is shown. (**C**) K7 ccRCC lung model treated with 1×10^6 *TRAC*- and *CD70*-edited (TCR^{KO} $CD70^{KO}$) CD70-1XX CAR T cells, *TRAC*-edited only (TCR^{KO} $CD70^{WT}$) CD70-1XX CAR T cells, or untransduced (UTD) T cells (donor 3). Tumor burden (total flux) was monitored using bioluminescence imaging (BLI). $n = 5$ mice per group, two-way ANOVA. (**D**) Evaluation of ablating CD70 on CD19-1XX CAR T cells in vivo. Tumor burden (total flux) and Kaplan-Meier survival analysis of NALM6-bearing mice treated with 5×10^5 $CD70^{KO}$ or $CD70^{WT}$ CD19-1XX CAR T cells, or UTD T cells (donor 4). Mice were rechallenged four times (at d35, d56, d102, and d138) with increasing NALM6 tumor doses. Tumor burden (total flux)

was monitored by BLI. $n = 5$ mice per group; ns, not significant; two-way ANOVA. In the survival analysis, P value determined by log-rank Mantel-Cox test (survival); ns, not significant.

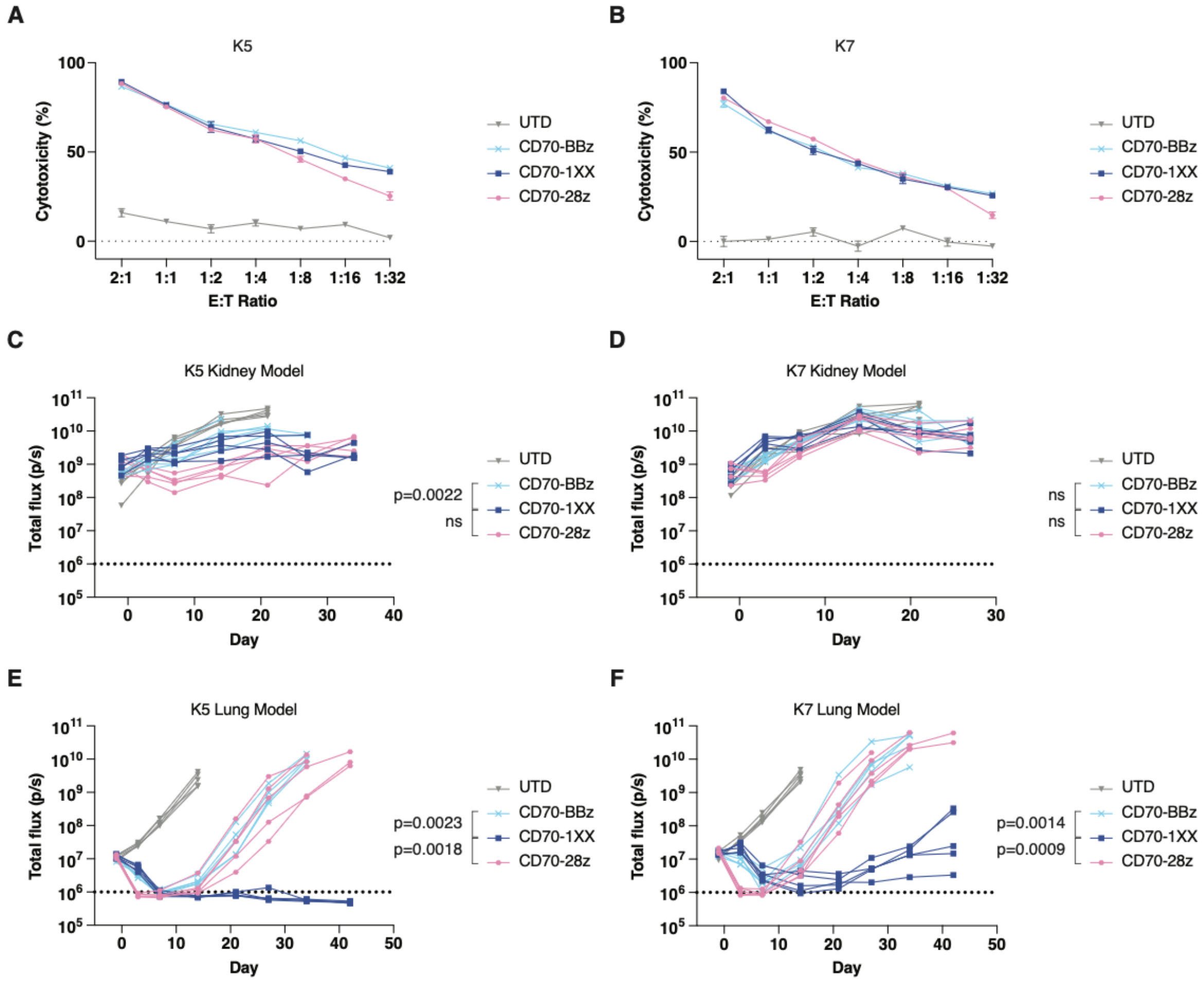

fig. S3. Comparison of 1XX with CD28- and 4-1BB-based CARs in kidney and lung model (related to Fig. 1).

(**A** and **B**) Cytotoxicity assay using an 18-hour bioluminescence assay, using firefly luciferase expressing K5 (A) or K7 (B) as target cells, which were incubated at the indicated E:T ratios with CD70-BBz, CD70-1XX, CD70-28z CAR T cells or untransduced (UTD) T cells (donor 6). $n = 3$, mean ± SEM. (**C** and **D**) Mice bearing tumor at the kidney site were treated with 2.5×10^6 CD70-BBz, CD70-1XX, CD70-28z CAR T cells or UTD T cells (donor 6) in the K5 kidney model (C) and K7 kidney model (D). Tumor burden (total flux) was monitored using bioluminescence imaging (BLI). $n = 5$ mice per group; ns, not significant; two-way ANOVA. (**E** and **F**) Mice bearing ccRCC tumor in the lungs were treated with 1×10^6 CD70-BBz, CD70-1XX, CD70-28z CAR T cells or UTD T cells (donor 6) in the K5 lung model (E) and K7 lung model (F). Tumor burden (total flux) is shown. $n = 5$ mice per group; two-way ANOVA.

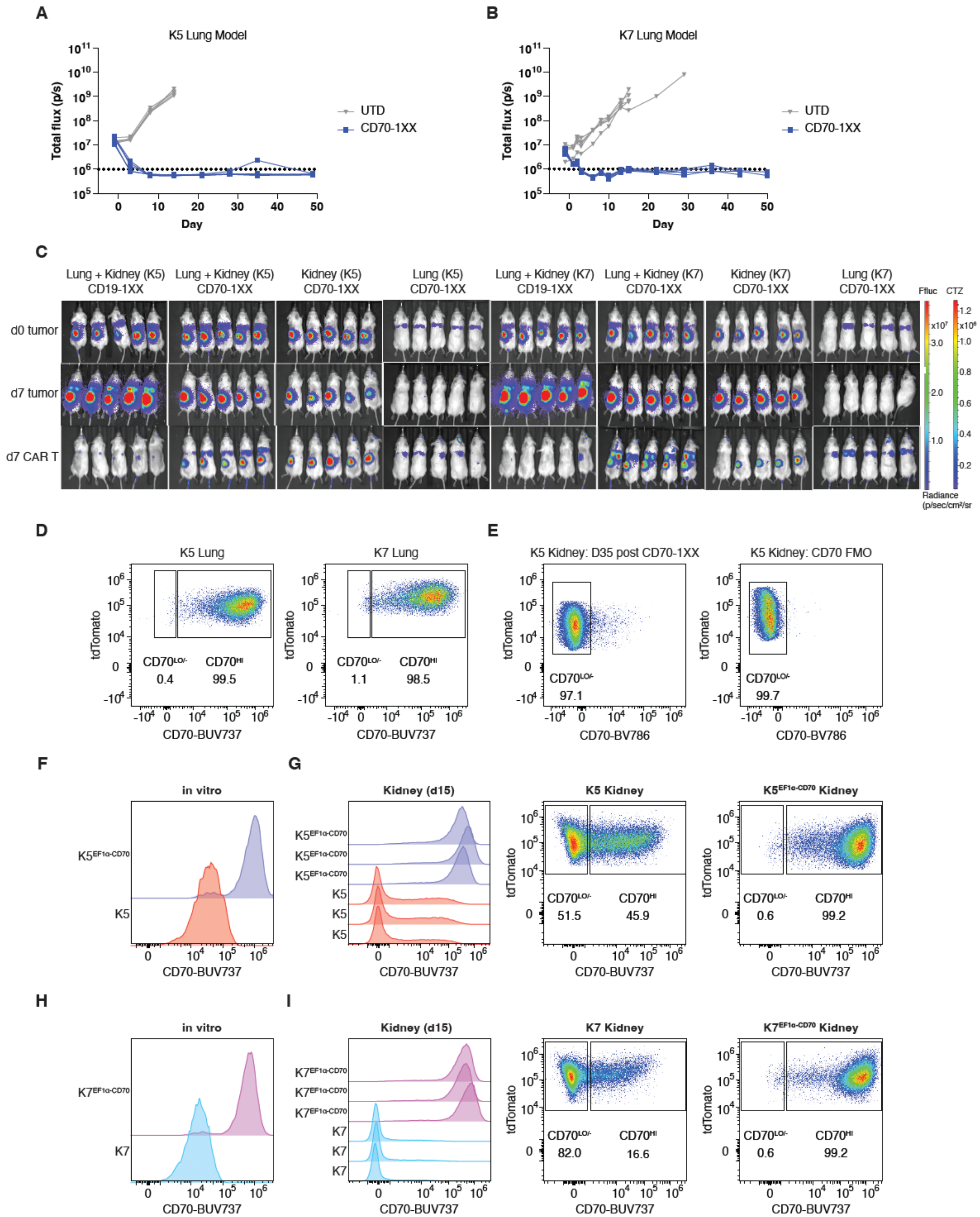

fig. S4. CD70 level and not CAR T cell trafficking accounts for CD70-CAR T cell failure at the kidney site (related to Fig. 1).

(**A** and **B**) K5 lung model (A) and K7 lung model (B) treated with 1 × 10^6 CD70-1XX CAR or untransduced (UTD) T cells [donor 7 in (A); donor 8 in (B)]. Tumor burden (total flux) is shown. n = 5 mice per group. Data is each representative of two independent in vivo experiments (with

two different donors). (**C**) CAR T cell trafficking study in vivo to detect CAR T cells and tumor by BLI following administration of coelenterazine or luciferin, respectively. NSG mice bearing tumor at both the lung and kidney site (“Lung + Kidney”), kidney site only (“Kidney”), or lung site only (“Lung”) treated with 2.5×10^6 CD70-1XX or CD19-1XX CAR T cells expressing a membrane bound Gaussia luciferase (donor 9). Day 0 depicts tumor burden prior to CAR T cell infusion. Tumor and CAR T cells 7 days after CAR T cell infusion are depicted highlighting CD70-1XX CAR T cell accumulation at both the tumor-bearing kidney and lung site, but only tumor eradication in lung site. Representative of two independent in vivo experiments (with two different donors). (**D**) Flow cytometric analysis of CD70 expression on tumor from the lung site of untreated mice. Data is representative of three mice per PDX. (**E**) Flow cytometric analysis of CD70 expression on refractory kidney tumor 35 days after treatment with 5×10^6 million CD70-1XX CAR T cells (donor 10). Representative of two independent in vivo experiments (with two different donors). (**F** to **I**) K5 and K7 PDX lines were stably transduced with a lentiviral vector encoding the EF1α promoter to constitutively express CD70 at the kidney site. Flow cytometric analysis of CD70 expression in vitro (F and H) and on ex-vivo kidney tumor (G and I) 15 days after tumor implantation. Representative flow cytometric plots shown on the right of the histograms. $n = 3$ mice per group.

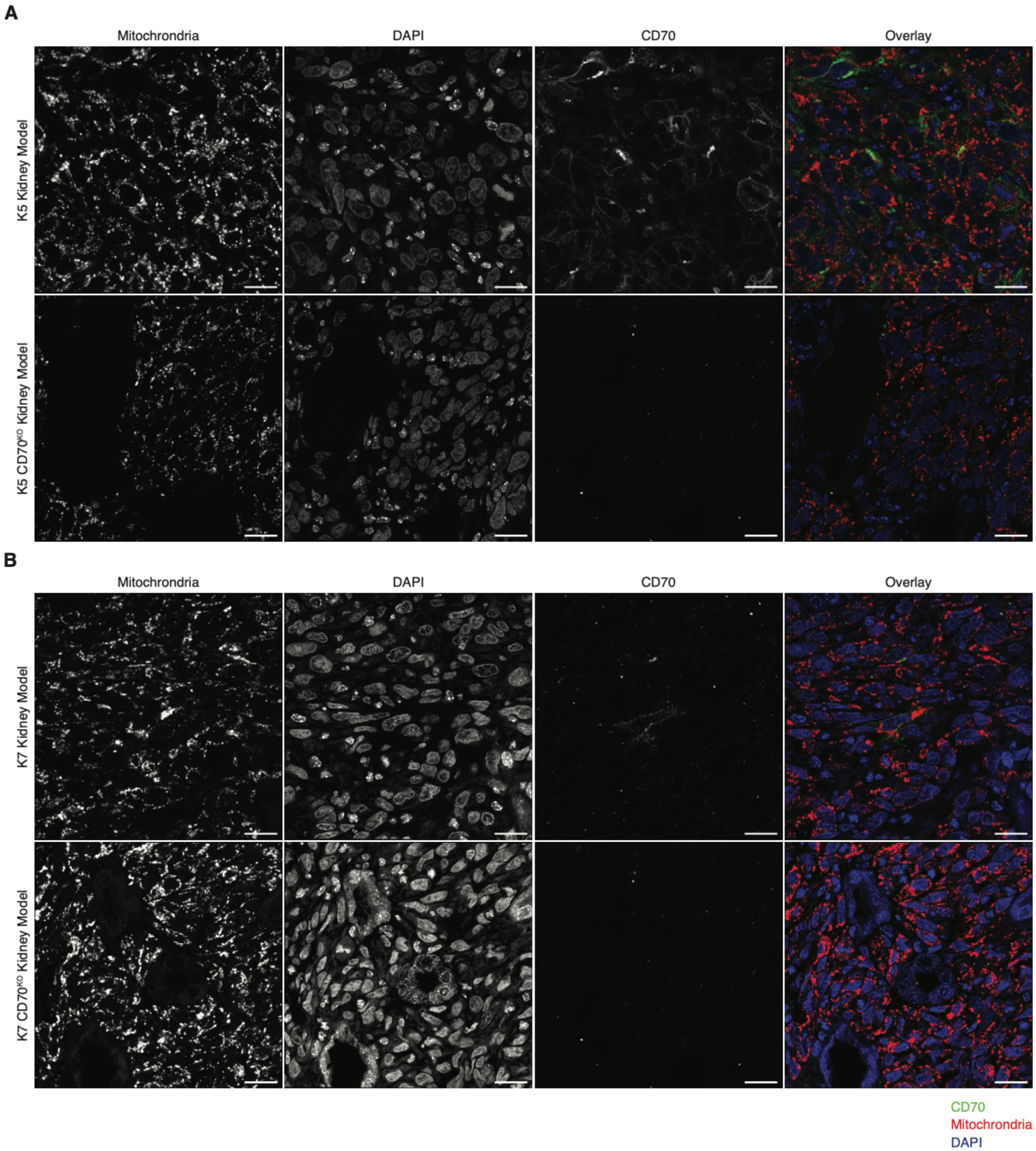

fig. S5. Representative overview of the field of view used for quantification of CD70 mean fluorescence intensity (related to Fig. 1).

Representative field of view used for the quantification of CD70 mean fluorescence intensity of individual tumor cells in K5 (**A**) and K7 (**B)** kidney models. K5 $CD70^{KO}$ and K7 $CD70^{KO}$ kidney models were used as respective controls. Overlay depicts human mitochondria (red), which labels human PDX in mouse tissue, CD70 (green) and DAPI (blue). Scale bar, 20µM.

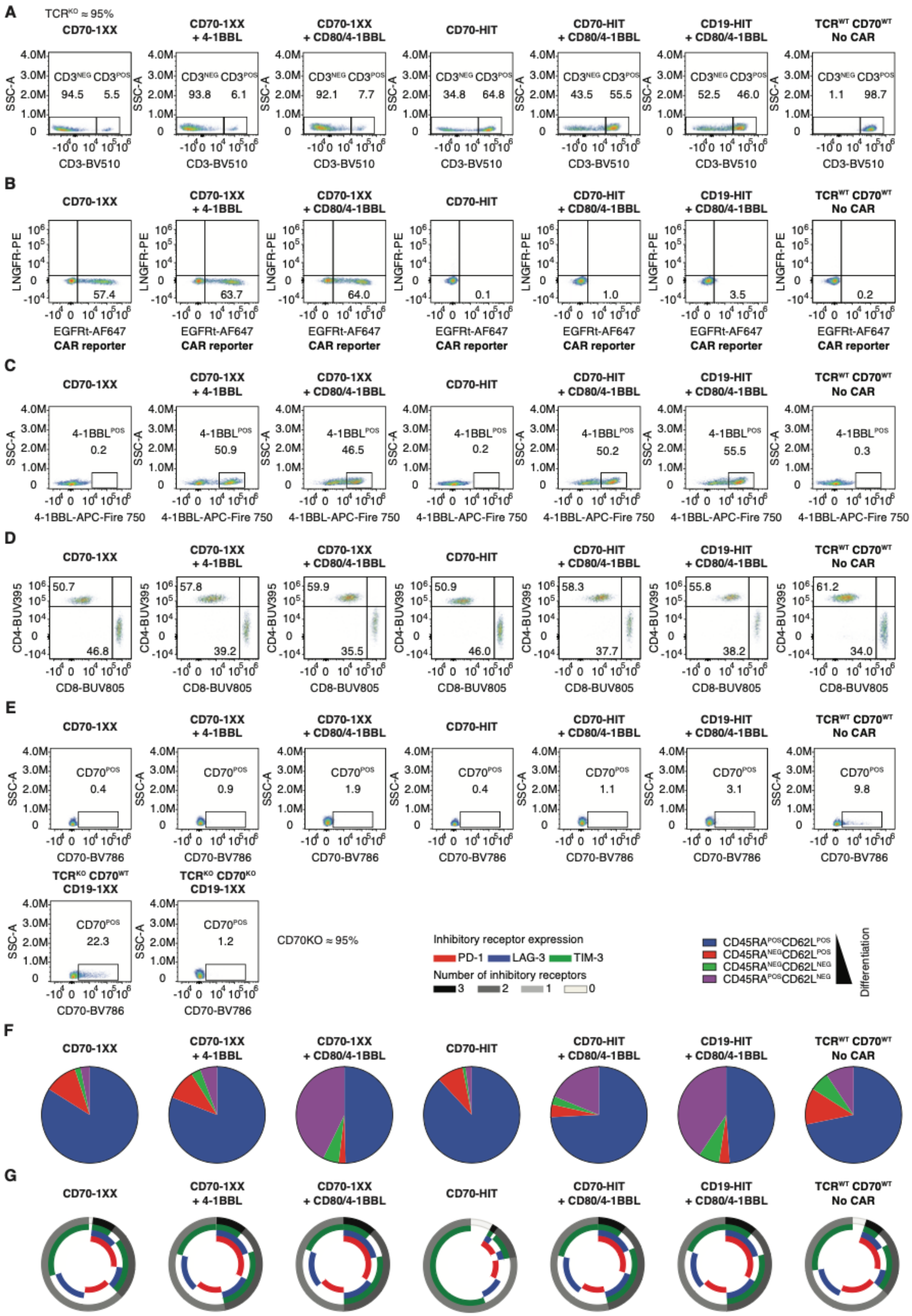
A
TCR^KO ≈ 95%
CD70-1XX
CD70-1XX + 4-1BBL
CD70-1XX + CD80/4-1BBL
CD70-HIT
CD70-HIT + CD80/4-1BBL
CD19-HIT + CD80/4-1BBL
TCR^WT CD70^WT No CAR
SSC-A
CD3-BV510
CD3^NEG CD3^POS
94.5 5.5
93.8 6.1
92.1 7.7
34.8 64.8
43.5 55.5
52.5 46.0
1.1 98.7
B
LNGFR-PE
EGFRt-AF647
CAR reporter
57.4
63.7
64.0
0.1
1.0
3.5
0.2
C
4-1BBL^POS
4-1BBL-APC-Fire 750
0.2
50.9
46.5
0.2
50.2
55.5
0.3
D
CD4-BUV395
CD8-BUV805
50.7 46.8
57.8 39.2
59.9 35.5
50.9 46.0
58.3 37.7
55.8 38.2
61.2 34.0
E
CD70^POS
CD70-BV786
0.4
0.9
1.9
0.4
1.1
3.1
9.8
TCR^KO CD70^WT CD19-1XX
22.3
TCR^KO CD70^KO CD19-1XX
1.2
CD70KO ≈ 95%
Inhibitory receptor expression
PD-1 LAG-3 TIM-3
Number of inhibitory receptors
3 2 1 0
CD45RA^POS CD62L^POS
CD45RA^NEG CD62L^POS
CD45RA^NEG CD62L^NEG
CD45RA^POS CD62L^NEG
Differentiation
F
G

fig. S6. Characterization of CAR and HIT expression in pre-infusion T cells.
For all experiments, the endogenous *TRAC* and *CD70* loci were ablated in infused T cells. Representative flow cytometric analysis 4 days after CRISPR-Cas9 *TRAC* and *CD70* editing, and *TRAC*-targeted HIT or CAR AAV6 transduction in donor 13 T cells (**A** to **G**). (**A**) The disruption of the endogenous *TRAC* locus ablated cell-surface CD3 expression. Approximately 95% of CAR T cells were $CD3^{NEG}$. CD3 expression is only observed in *TRAC*-HIT T cells, where it is restored to the cell surface due to the engineered expression of VL-Cα and VH-Cβ in the HIT receptor. CD3 re-expression was used to determine HIT transduction efficiency. CD3 remains undetected when knocking-in the CD70-1XX CAR. (**B**) *TRAC*-targeted CAR was coexpressed with a truncated EGFR (EGFRt) reporter, which was used to determine CAR transduction efficiency. (**C**) 24 hours after *TRAC*-targeted AAV6 transduction, HIT T cells were transduced with SFG γ-retroviral vectors to coexpress CD80 and 4-1BBL costimulatory ligands. Coexpression of CD80 and 4-1BBL, or 4-1BBL alone with CD70-1XX CAR is shown. (**D**) Percentage of $CD4^{POS}$ and $CD8^{POS}$ T cells in manufactured T cells. (**E**) $CD70^{KO}$ efficiency ≈ 95% by flow cytometry. (**F**) Phenotype of CAR or HIT T cells as demonstrated by percentage of CD62L/CD45RA expression. (**G**) Percentage of CAR or HIT T cells expressing inhibitory receptors (PD-1, LAG-3 and TIM-3).

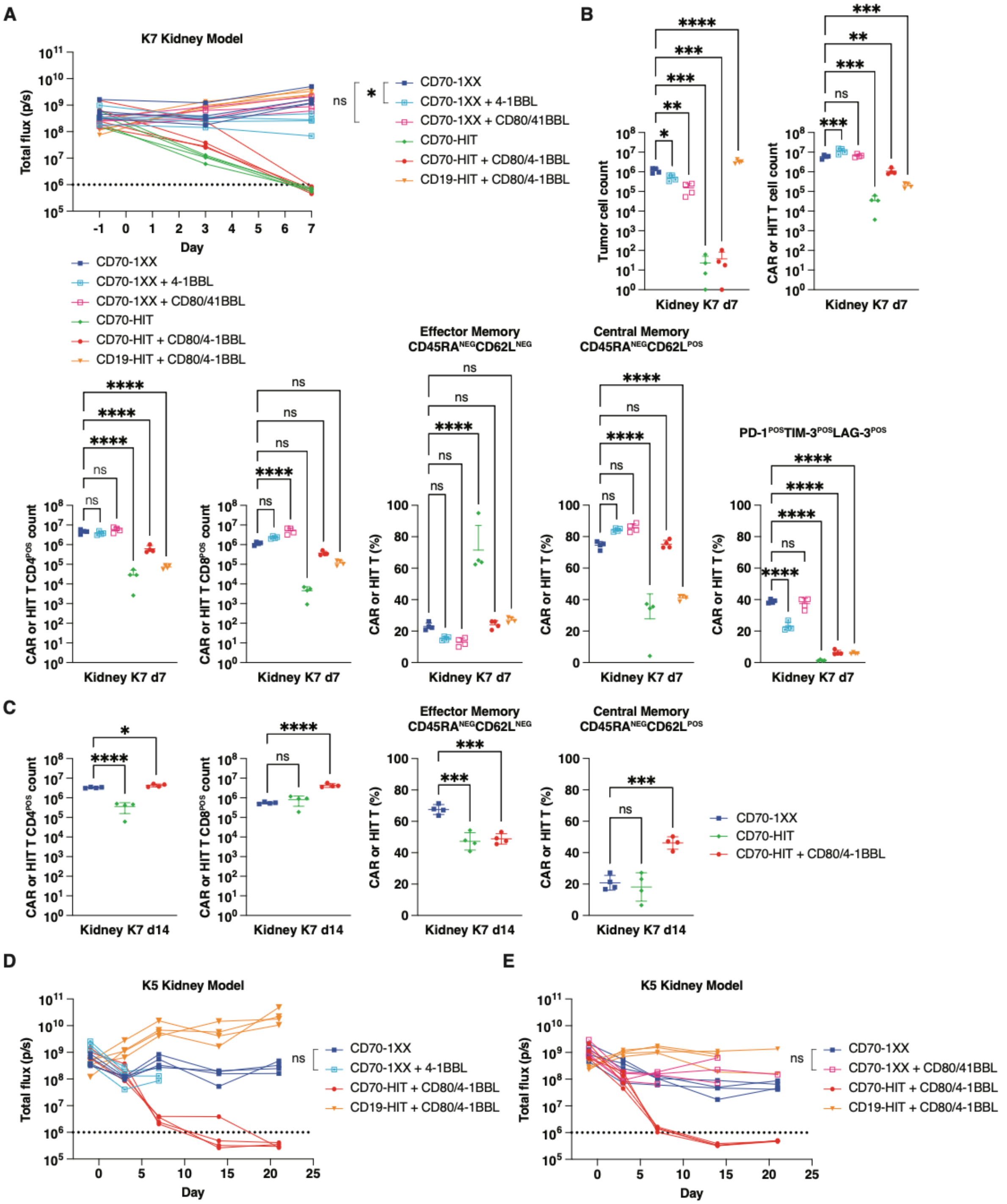

fig. S7. Coexpression of CD80 and 4-1BBL costimulatory ligands does not enhance CD70-CAR efficacy against K5 or K7 kidney tumors in vivo (related to Fig. 2).
(**A**) Mice bearing K7 tumor at the kidney site were treated with 2.5 × 10^6 CD70-1XX CAR, CD70-1XX CAR coexpressing 4-1BBL, CD70-1XX CAR coexpressing CD80 and 4-1BBL, CD70-HIT, CD70-HIT coexpressing CD80 and 4-1BBL, and CD19-HIT T cells coexpressing CD80 and 4-1BBL (donor 13). Tumor burden (total flux) is shown. n = 5 mice per group; ns, not significant; **P* <0.05; two-way ANOVA. Representative of three independent in vivo experiments (with three different donors). (**B**) Mice from (A) were euthanized at day 7 after T

cell infusion. Kidney tumors were analyzed by flow cytometry post treatment for: the number of tumor cells; the number of CAR and HIT T cells; the number of $CD4^{POS}$ and $CD8^{POS}$ CAR or HIT T cells; the percentage of effector memory ($CD45RA^{NEG}CD62L^{NEG}$) and central memory ($CD45RA^{NEG}CD62L^{POS}$) CAR or HIT T cells; and the percentage of CAR or HIT T cells expressing inhibitory receptors (PD-1, LAG-3 and TIM-3). *n* = 4 mice per group; mean ± SD; ns, not significant; **P* <0.05; ***P* <0.01; ****P* <0.001; *****P* <0.0001; one-way ANOVA. (**C**) Kidney tumors isolated at day 14 after T cell infusion are shown here and in Fig. 2, D to F. *n* = 4 mice per group; mean ± SD; ns, not significant; **P* <0.05; ****P* <0.001; *****P* <0.0001; one-way ANOVA. Characterization of pre-infusion donor 13 CAR or HIT T cells are shown in fig. S6. (**D**) Mice bearing K5 tumor at the kidney site were treated with 2.5×10^6 CD70-1XX CAR, CD70-1XX CAR coexpressing 4-1BBL, CD70-HIT coexpressing CD80 and 4-1BBL, and CD19-HIT T cells coexpressing CD80 and 4-1BBL (donor 14). Tumor burden (total flux) is shown. *n* = 4 mice per group; ns, not significant; two-way ANOVA. (**E**) Mice bearing K5 tumor at the kidney site were treated with 2.5×10^6 CD70-1XX CAR, CD70-1XX CAR coexpressing CD80 and 4-1BBL, CD70-HIT coexpressing CD80 and 4-1BBL, and CD19-HIT T cells coexpressing CD80 and 4-1BBL (donor 15). Tumor burden (total flux) is shown. *n* = 4 mice per group; ns, not significant; two-way ANOVA.

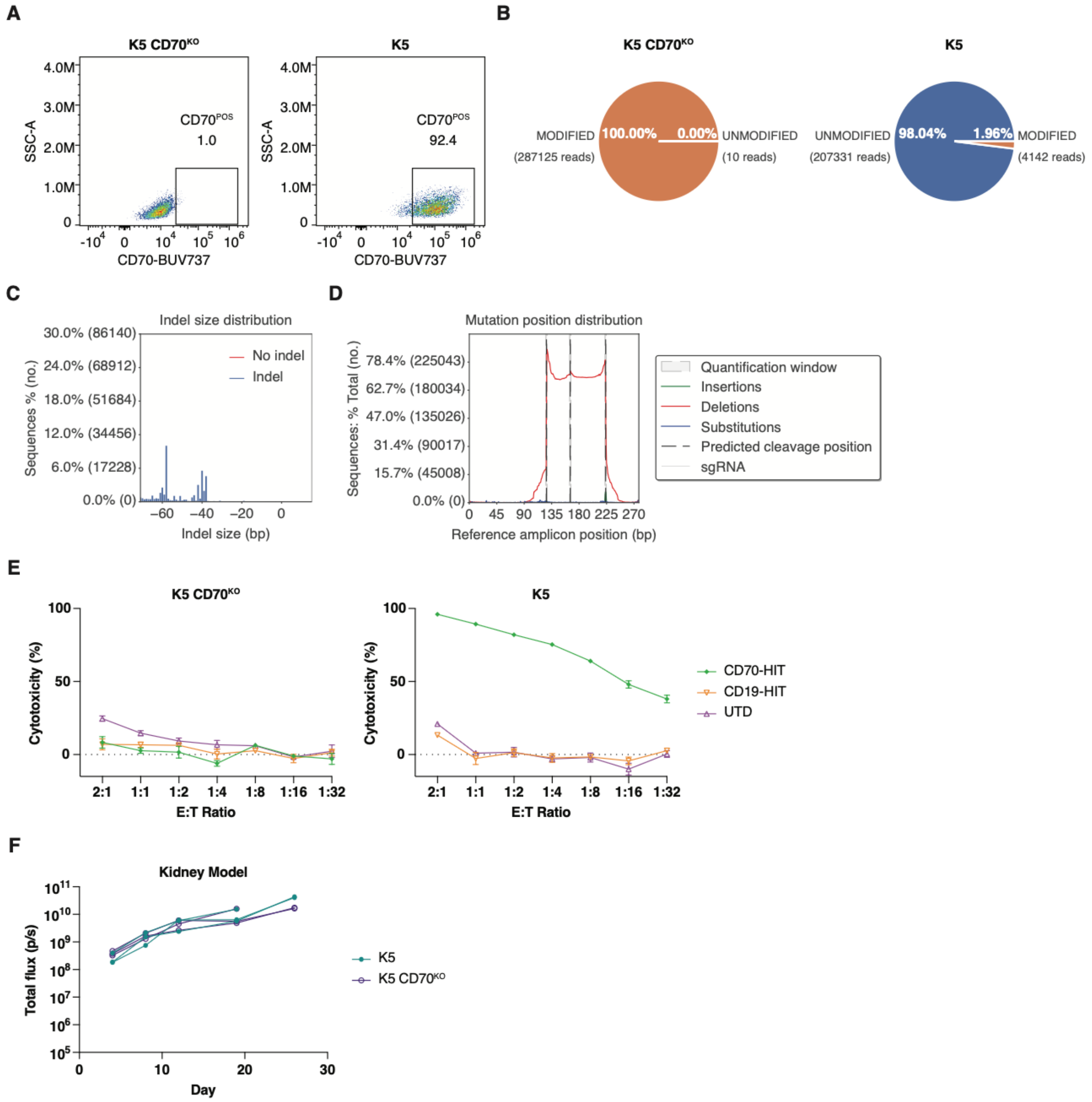

fig. S8. CRISPR-Cas9 CD70-knockout K5 tumor (related to Fig. 2).
(**A**) Flow cytometric analysis showing in vitro expression of CD70 on K5 and CRISPR-Cas9 CD70-knockout (KO) K5 tumor, K5 $CD70^{KO}$. (**B** to **D**) CRISPResso2 analysis of genome editing by deep sequencing. (**B**) Alignment and editing frequency of reads as determined by the percentage and number of sequence reads showing unmodified and modified alleles. (**C**) Frequency distribution of alleles with indels (blue) and without indels (red). (**D**) Frequency of insertions, deletions, and substitutions across the entire amplicon. (**E**) Cytotoxicity assay using an 18-hour bioluminescence assay, using firefly luciferase expressing *CD70*-edited and unedited K5 PDX as target cells, which were incubated at the indicated E:T ratios with CD70-HIT, CD19-HIT or untransduced (UTD) T cells (donor 16). $n = 3$, mean ± SEM. (**F**) Growth kinetics of *CD70*-edited and unedited tumor in K5 kidney model measured by bioluminescence imaging (total flux is shown).

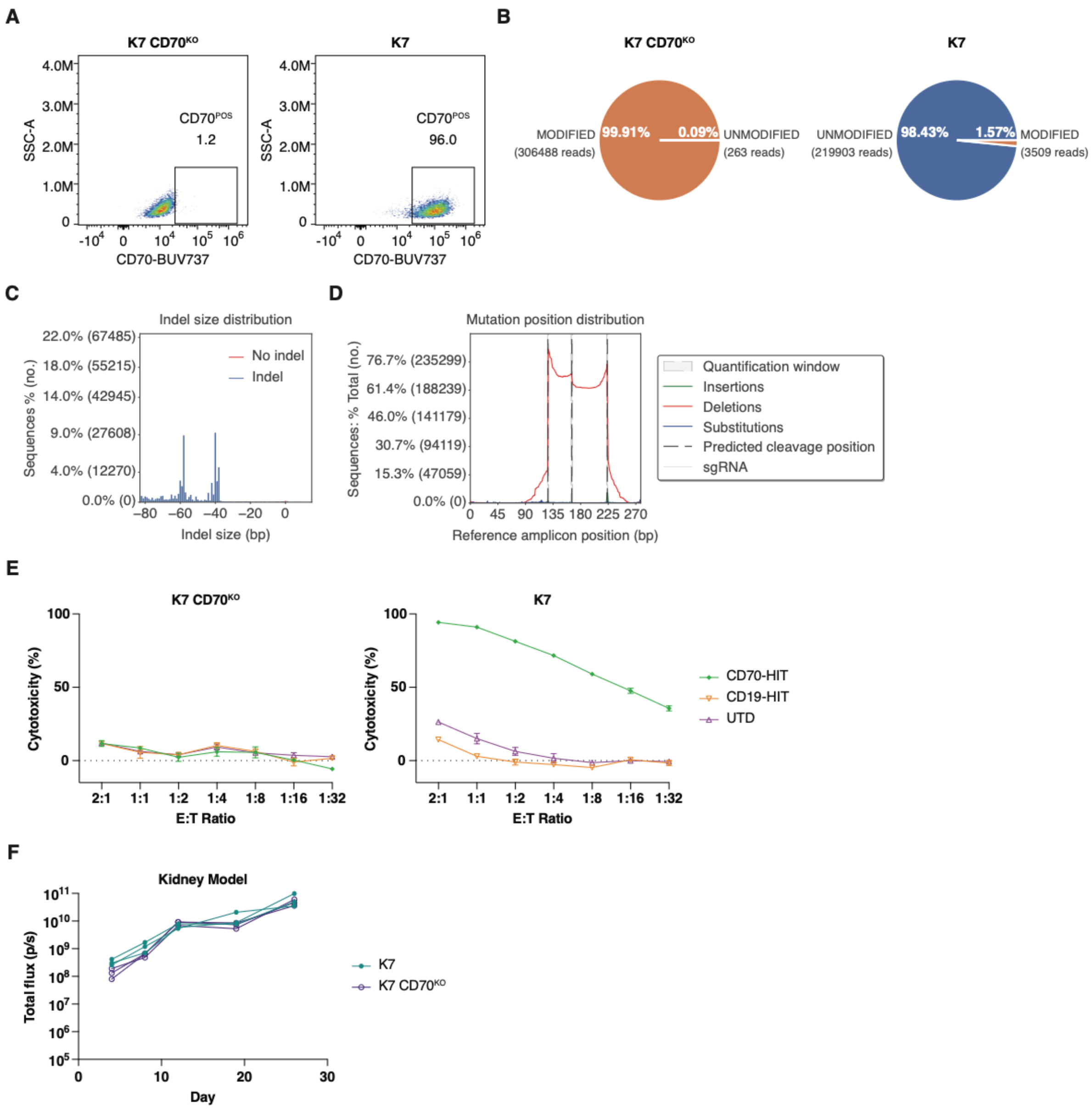

fig. S9. CRISPR-Cas9 CD70-knockout K7 tumor (related to Fig. 2).
(**A**) Flow cytometric analysis showing in vitro expression of CD70 on K7 and CRISPR-Cas9 CD70-knockout (KO) K7 tumor, K7 $CD70^{KO}$. (**B** to **D**) CRISPResso2 analysis of genome editing by deep sequencing. (**B**) Alignment and editing frequency of reads as determined by the percentage and number of sequence reads showing unmodified and modified alleles. (**C**) Frequency distribution of alleles with indels (blue) and without indels (red). (**D**) Frequency of insertions, deletions, and substitutions across the entire amplicon. (**E**) Cytotoxicity assay using an 18-hour bioluminescence assay, using firefly luciferase expressing *CD70*-edited and unedited K7 PDX as target cells, which were incubated at the indicated E:T ratios with CD70-HIT, CD19-HIT or untransduced (UTD) T cells (donor 16). $n = 3$, mean ± SEM. (**F**) Growth kinetics of *CD70*-edited and unedited tumor in K7 kidney model measured by bioluminescence imaging (total flux is shown).

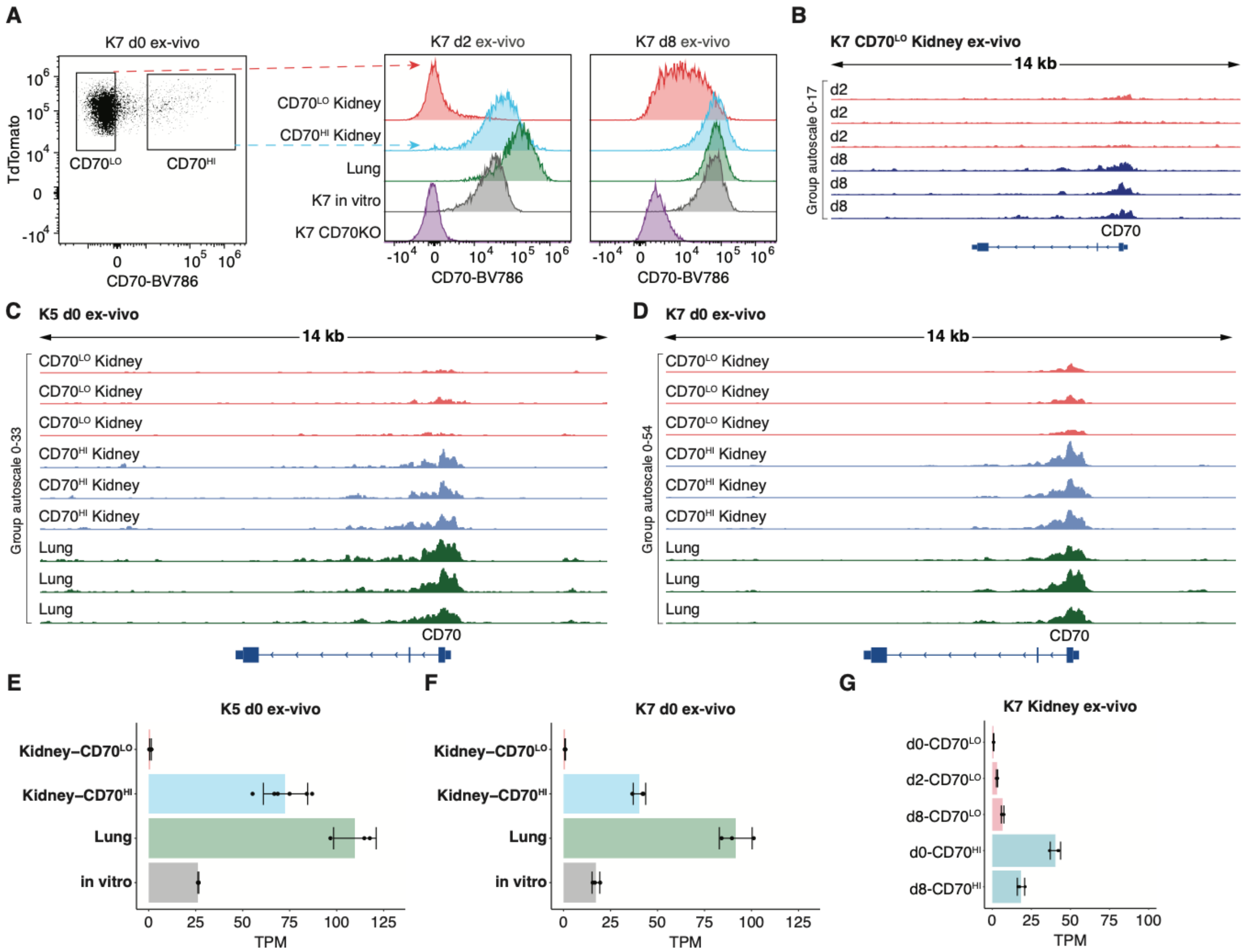

fig. S10. CD70 is epigenetically regulated in K7 PDX (related to Fig. 3).
(**A**) CD70^{LO} and CD70^{HI} tumor cells from mice bearing K7 PDX tumor at both kidney and lung sites were isolated by fluorescence-activated cell sorting. Representative gating strategy to sort CD70^{LO} and CD70^{HI} tumor cells from the kidney site, denoted as day 0 of isolation, "d0 ex-vivo". Isolated CD70^{LO} and CD70^{HI} tumor cells from the kidney site, and CD70^{HI} tumor cells from the lung site were then cultured for 8 days. Flow cytometric analysis of CD70 expression at day 2 in culture ("d2 ex-vivo") and day 8 in culture ("d8 ex-vivo"). (**B**) Genome browser view of chromatin accessibility at the *CD70* gene in CD70^{LO} K7 tumor cells from the kidney site at d2 and d8 ex-vivo as determined by assay for transposase-accessible chromatin with sequencing (ATAC-seq). Chromatin accessibility corresponds with restoration of CD70 expression. (**C** and **D**) Genome browser view of chromatin accessibility at the *CD70* gene in d0 ex-vivo CD70^{LO} and CD70^{HI} tumor cells from the kidney site, and CD70^{HI} tumor cells from the lung site as determined by ATAC-seq in K5 (C) and K7 (CD70^{HI} versus CD70^{LO} kidney tumor cells: adjusted P value = 1.57×10^{-13}) (D). n = 3 mice per PDX. (**E** and **F**) CD70 transcript expression in CD70^{LO} and CD70^{HI} tumor cells from the kidney, and tumor cells from the lung site in K5 (E) and K7 (F). n = 3 mice per PDX, mean ± SD. (**G**) CD70 transcript expression in CD70^{LO} and CD70^{HI} K7 tumor cells from the kidney site at d0, d2 and d8 ex-vivo. n = 3 mice per PDX, mean ± SD.

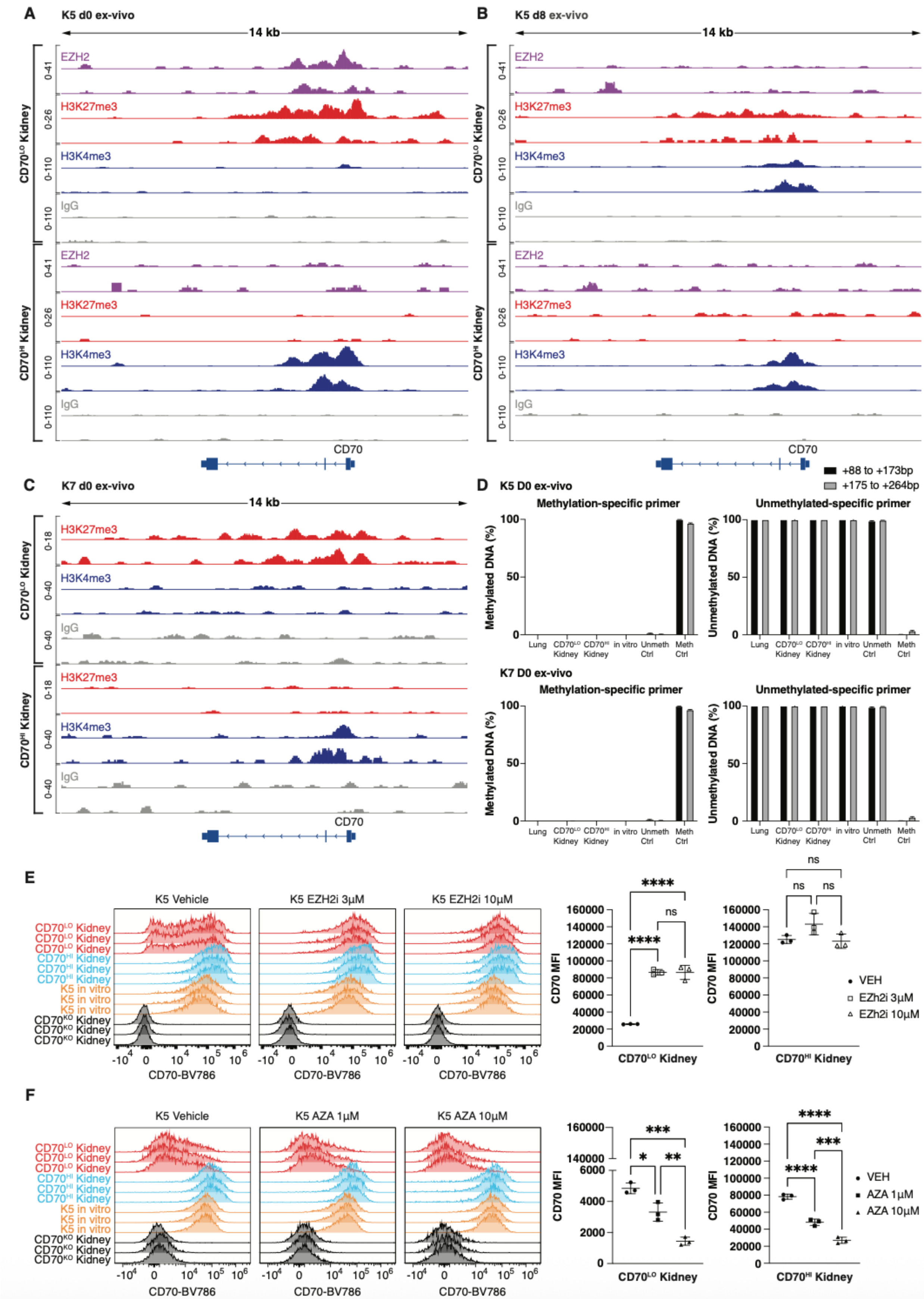
A
K5 d0 ex-vivo
14 kb
CD70LO Kidney
CD70HI Kidney
EZH2
H3K27me3
H3K4me3
IgG
CD70
B
K5 d8 ex-vivo
C
K7 d0 ex-vivo
D
K5 D0 ex-vivo
K7 D0 ex-vivo
Methylation-specific primer
Unmethylated-specific primer
Methylated DNA (%)
Unmethylated DNA (%)
+88 to +173bp
+175 to +264bp
Lung
CD70LO Kidney
CD70HI Kidney
in vitro
Unmeth Ctrl
Meth Ctrl
E
K5 Vehicle
K5 EZH2i 3µM
K5 EZH2i 10µM
CD70LO Kidney
CD70HI Kidney
K5 in vitro
CD70KO Kidney
CD70-BV786
CD70 MFI
VEH
EZh2i 3µM
EZh2i 10µM
F
K5 AZA 1µM
K5 AZA 10µM
AZA 1µM
AZA 10µM

fig. S11. CD70 is regulated by H3K27me3 and not DNA methylation in K5 and K7 (related to Fig. 3).

(**A**) Genome browser views of the *CD70* genomic region at d0 ex-vivo showing enrichment for EZH2 and repressive H3K27me3 histone modifications at the *CD70* locus in CD70^{LO} K5 tumor cells from the kidney, and activating H3K4me3 histone modifications at the *CD70* locus in CD70^{HI} K5 tumor cells from the kidney, assessed by Cleavage Under Targets & Release Using Nuclease (CUT&RUN). (**B**) As CD70 expression increased at day 8 ex-vivo, there was loss of H3K27me3 and a gain of H3K4me3 at the *CD70* locus in CD70^{LO} K5 tumor cells from the kidney site. (**C**) Genome browser views of the *CD70* genomic region at d0 ex-vivo showing enrichment for repressive H3K27me3 histone modifications at the *CD70* locus in CD70^{LO} K7 tumor cells from the kidney, and activating H3K4me3 histone modifications at the *CD70* locus in CD70^{HI} K7 tumor cells from the kidney, assessed by CUT&RUN. (**D**) Percentage of DNA methylated in the *CD70* promoter and 1st exon in genomic DNA isolated from d0 ex-vivo CD70^{LO} and CD70^{HI} tumor cells from the kidney site, and tumor cells from the lung site. Genomic DNA was subjected to bisulfite conversion and then digital droplet PCR using methylation-specific primers. Methylated control (Meth Ctrl) is genomic DNA from Jurkat cells enzymatically methylated with CpG methyltransferase. Unmethylated control (Unmeth Ctrl) is unmethylated DNA. n = 3 mice per PDX, mean ± SD. (**E**) D0 ex-vivo CD70^{LO} and CD70^{HI} K5 tumor cells from then kidney site were treated with either DMSO (vehicle) or tazemetostat (EZH2 inhibitor, EZH2i 3μM or EZH2i 10μM) for 8 days. CD70 mean fluorescence intensity (MFI) was analyzed by flow cytometry. n = 3 mice per condition; mean ± SD; ns, not significant; ****P <0.0001; one-way ANOVA. (**F**) D0 ex-vivo CD70^{LO} and CD70^{HI} K5 tumor cells from the kidney site were treated with either DMSO (vehicle) or azacytidine (AZA, 1μM or AZA 10μM) for 72 hours. CD70 mean fluorescence intensity (MFI) was analyzed by flow cytometry. n = 3 mice per condition, mean ± SD, *P <0.05; **P <0.01; ***P <0.001; ****P <0.0001; one-way ANOVA.

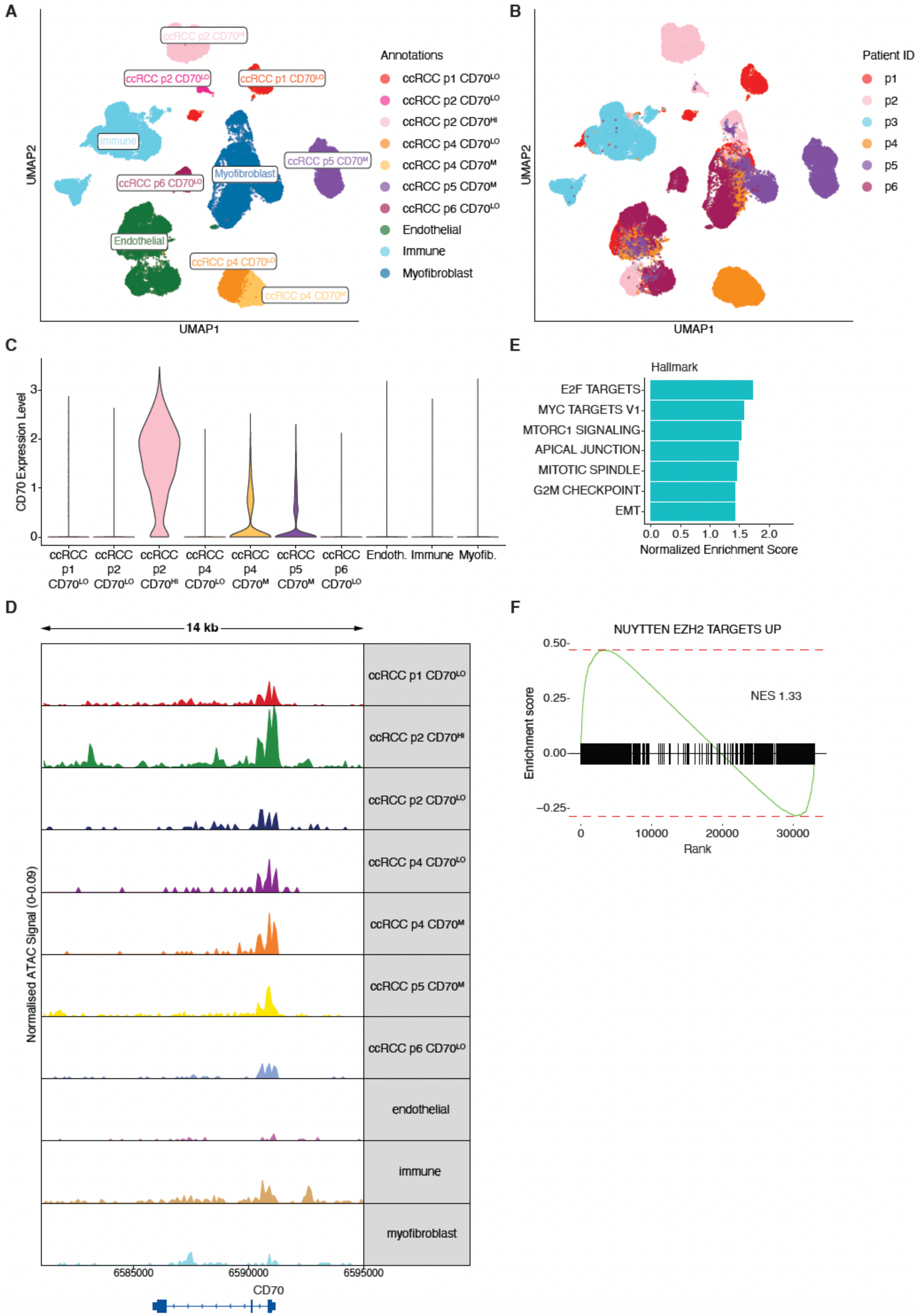
A
UMAP2
UMAP1
Annotations
ccRCC p1 CD70LO
ccRCC p2 CD70LO
ccRCC p2 CD70HI
ccRCC p4 CD70LO
ccRCC p4 CD70M
ccRCC p5 CD70M
ccRCC p6 CD70LO
Endothelial
Immune
Myofibroblast
B
Patient ID
p1
p2
p3
p4
p5
p6
C
CD70 Expression Level
ccRCC p1 CD70LO
ccRCC p2 CD70LO
ccRCC p2 CD70HI
ccRCC p4 CD70LO
ccRCC p4 CD70M
ccRCC p5 CD70M
ccRCC p6 CD70LO
Endoth.
Immune
Myofib.
E
Hallmark
E2F TARGETS
MYC TARGETS V1
MTORC1 SIGNALING
APICAL JUNCTION
MITOTIC SPINDLE
G2M CHECKPOINT
EMT
Normalized Enrichment Score
D
14 kb
Normalised ATAC Signal (0-0.09)
endothelial
immune
myofibroblast
6585000
6590000
6595000
CD70
F
NUYTTEN EZH2 TARGETS UP
Enrichment score
NES 1.33
Rank

fig. S12. ccRCC patient primary tumors profiled by 10× Multiome platform (related to Fig. 4).

(**A**) Uniform manifold approximation and projection (UMAP) showing all cell types across the six ccRCC patient samples. Tumor cells are further annotated by CD70 expression level. (**B**) UMAP colored by patient IDs. (**C**) Violin plot of CD70 expression level for endothelial, myofibroblasts, immune cells and individual patient's tumor, further annotated by CD70-negative/low ($CD70^{LO}$), CD70-medium ($CD70^{M}$) and CD70-high ($CD70^{HI}$) expression. (**D**) Chromatin accessibility of the *CD70* gene for all cell types in the data as annotated in (A). (**E**) Normalized enrichment scores of significantly upregulated gene sets in $CD70^{HI}$ versus $CD70^{M/LO}$ tumor as determined by GSEA using MSigDB Hallmark gene sets (*P*adj < 0.05). (**F**) Normalized enrichment score (NES) of genes upregulated after knockdown of EZH2 by RNA interference enriched in $CD70^{HI}$ tumor compared to $CD70^{M/LO}$ as determined by GSEA using MSigDB C2 curated gene set (*P*adj = 0.007).

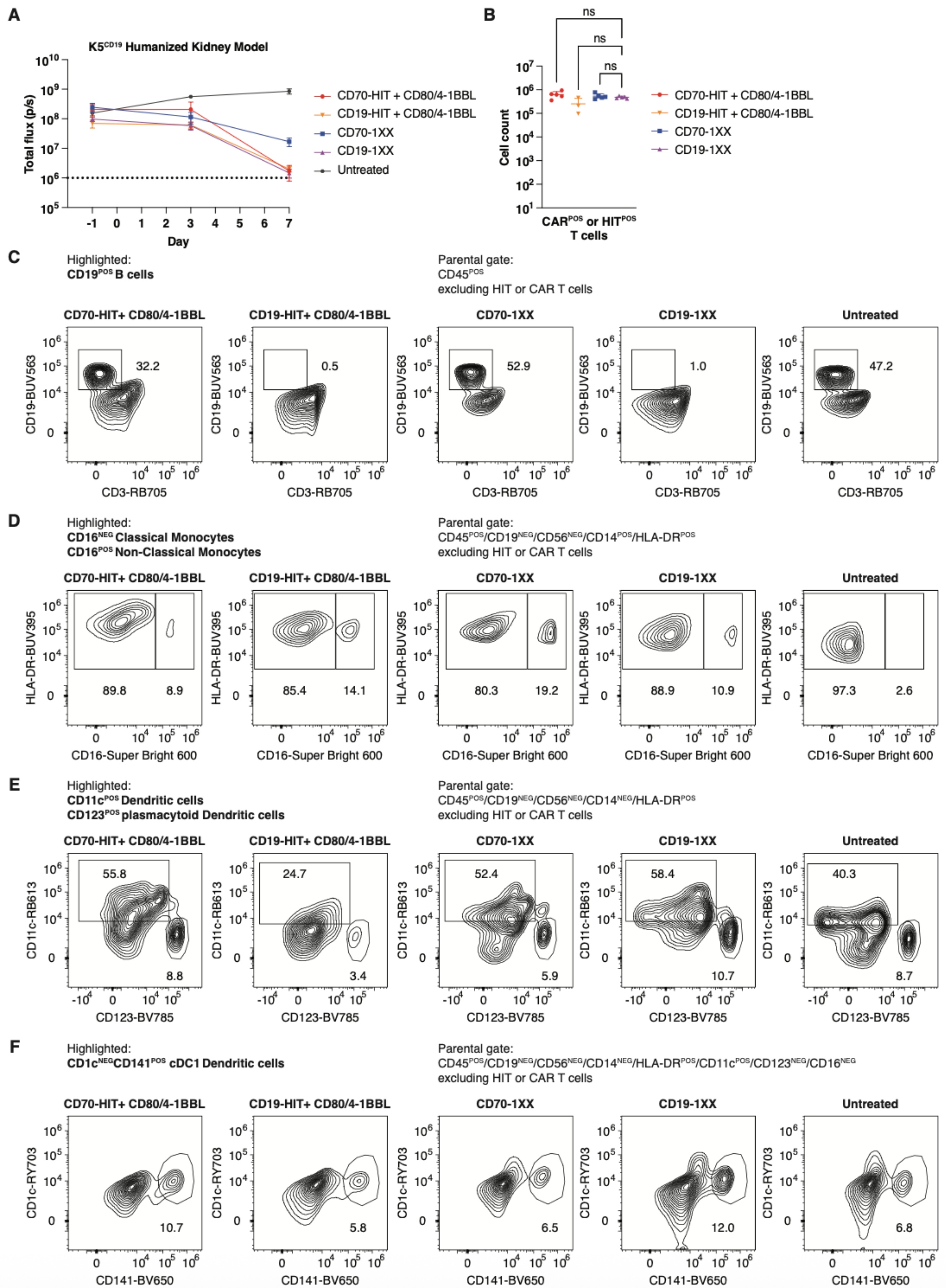
A
K5CD19 Humanized Kidney Model
Total flux (p/s)
Day
CD70-HIT + CD80/4-1BBL
CD19-HIT + CD80/4-1BBL
CD70-1XX
CD19-1XX
Untreated
B
ns
ns
ns
Cell count
CARPOS or HITPOS T cells
C
Highlighted:
CD19POS B cells
Parental gate:
CD45POS
excluding HIT or CAR T cells
CD70-HIT+ CD80/4-1BBL
CD19-HIT+ CD80/4-1BBL
CD70-1XX
CD19-1XX
Untreated
CD19-BUV563
CD3-RB705
32.2
0.5
52.9
1.0
47.2
D
Highlighted:
CD16NEG Classical Monocytes
CD16POS Non-Classical Monocytes
Parental gate:
CD45POS/CD19NEG/CD56NEG/CD14POS/HLA-DRPOS
excluding HIT or CAR T cells
HLA-DR-BUV395
CD16-Super Bright 600
89.8
8.9
85.4
14.1
80.3
19.2
88.9
10.9
97.3
2.6
E
Highlighted:
CD11cPOS Dendritic cells
CD123POS plasmacytoid Dendritic cells
Parental gate:
CD45POS/CD19NEG/CD56NEG/CD14NEG/HLA-DRPOS
excluding HIT or CAR T cells
CD11c-RB613
CD123-BV785
55.8
8.8
24.7
3.4
52.4
5.9
58.4
10.7
40.3
8.7
F
Highlighted:
CD1cNEGCD141POS cDC1 Dendritic cells
Parental gate:
CD45POS/CD19NEG/CD56NEG/CD14NEG/HLA-DRPOS/CD11cPOS/CD123NEG/CD16NEG
excluding HIT or CAR T cells
CD1c-RY703
CD141-BV650
10.7
5.8
6.5
12.0
6.8

fig. S13. Humanized ccRCC PDX model to assess CD70-HIT T cell hematotoxicity (related to Fig. 4).

(**A**) ccRCC tumor burden (total flux) of NBSGW mice bearing K5 tumor constitutively expressing CD19 ($K5^{CD19}$) at the kidney site. Anti-tumor response was assessed upon receiving 2.5×10^6 of either CD70-1XX CAR, CD19-1XX CAR, CD70-HIT+CD80/4-1BBL, CD19-HIT+CD80/4-1BBL T cells (donor 18) or no treatment. *n* = 5 mice per group, mean ± SEM. (**B** to **F**) Mice were euthanized at day 9 after T cell infusion. (**B**) Kidney tumors were analyzed by flow cytometry for the number of CAR and HIT T cells. *n* = 3 to 5 mice per group; mean ± SD; ns, not significant; one-way ANOVA. (**C** to **F**) Impact on normal human hematopoiesis is shown in Fig. 4G. Representative flow cytometric analysis of normal human hematopoietic cells from bone marrow on day 9 after T cell infusion. (**C**) $CD19^{POS}$ B cells within total human $CD45^{POS}$ cells (excluding adoptively transferred $CD3^{POS}$ HIT T cells and $EGFRt^{POS}$ CAR T cells). (**D**) $CD14^{POS}/CD16^{NEG}$ classical monocytes and $CD14^{POS}/CD16^{POS}$ non-classical monocytes within $CD3^{NEG}/CD19^{NEG}$ human $CD45^{POS}$ cells. (**E**) $CD11c^{POS}/CD123^{NEG}$ conventional dendritic cells and $CD11c^{NEG}/CD123^{POS}$ plasmacytoid dendritic cells within $CD3^{NEG}/CD19^{NEG}/CD56^{NEG}/CD14^{NEG}$ human $CD45^{POS}$ cells. (**F**) $CD1c^{NEG}/CD141^{POS}$ cDC1 dendritic cells within $CD3^{NEG}/CD19^{NEG}/CD56^{NEG}/CD14^{NEG}/CD11c^{POS}/CD123^{NEG}/CD16^{NEG}$ human $CD45^{POS}$ cells.

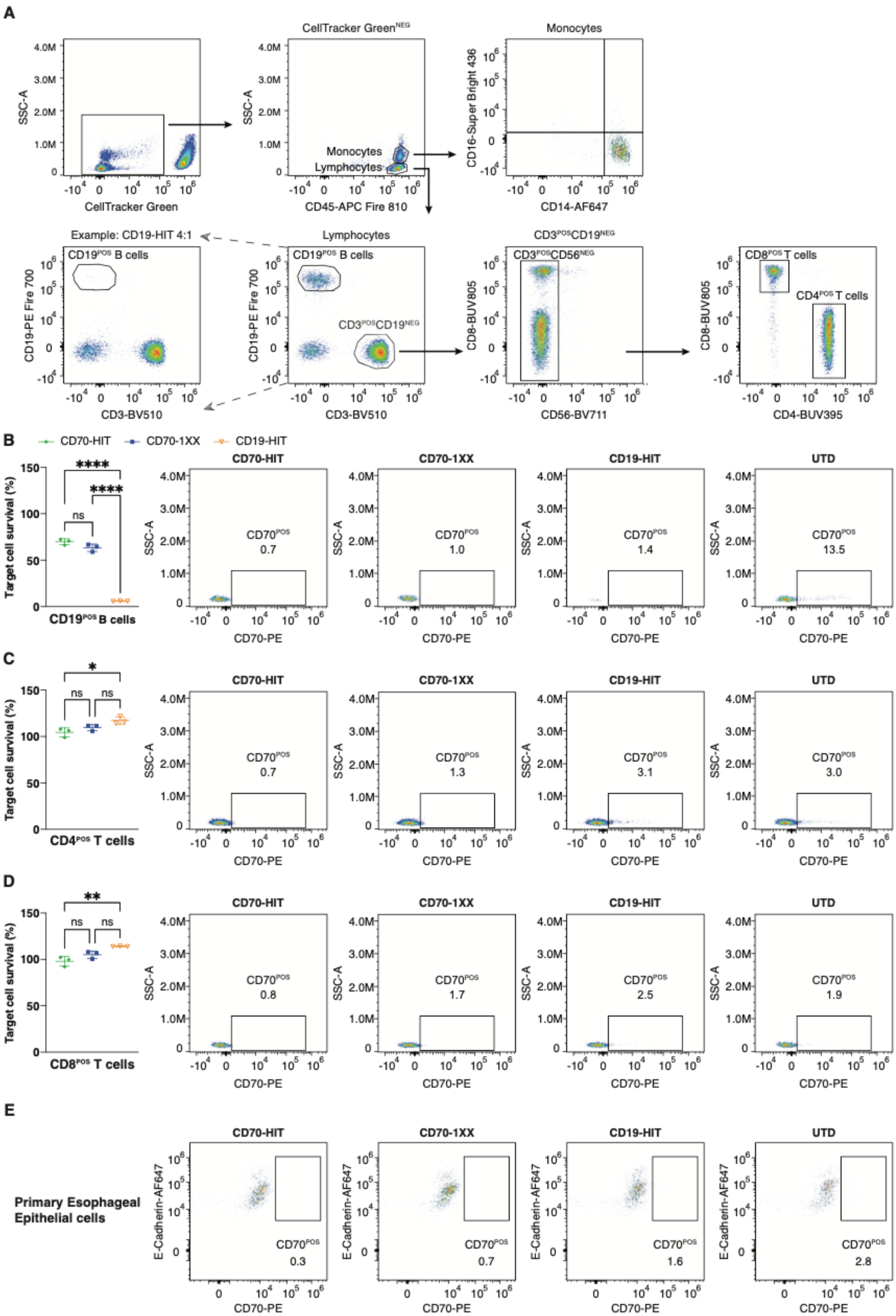
A
CellTracker Green
SSC-A
CellTracker Green^NEG
CD45-APC Fire 810
Monocytes
Lymphocytes
CD16-Super Bright 436
CD14-AF647
Example: CD19-HIT 4:1
CD19^POS B cells
CD19-PE Fire 700
CD3-BV510
CD3^POS CD19^NEG
CD3^POS CD56^NEG
CD8-BUV805
CD56-BV711
CD8^POS T cells
CD4^POS T cells
CD4-BUV395
B
CD70-HIT
CD70-1XX
CD19-HIT
UTD
Target cell survival (%)
CD19^POS B cells
CD70-PE
CD70^POS
C
CD4^POS T cells
D
CD8^POS T cells
E
Primary Esophageal Epithelial cells
E-Cadherin-AF647

fig. S14. CD70-HIT in vitro cytotoxicity against adult human immune cells (related to Fig. 4).

(A to **D)** The in vitro cytotoxicity of autologous CD70-HIT, CD70-1XX CAR, CD19-HIT or untransduced (UTD) T cells against PBMCs at an effector to target ratio of 4:1 (donor 19). CD19-HIT T cells serve as an internal positive control for expected on-target activity against CD19-positive B cells, and a negative control for on-target activity against T cells, which do not express CD19. HIT, CAR or UTD T cells were pre-labelled with CellTracker Green before incubation with PBMCs. Cytotoxicity and CD70 expression were evaluated by flow cytometry 18 hours after co-culture. (**A**) Flow cytometric gating strategy of peripheral blood mononuclear cells (PBMCs). (**B** to **D)** On the left, target cell survival. Survival is relative to cells co-cultured with untransduced T cells. On the right, flow cytometric plots of CD70 expression on indicated cell type 18 hours after co-culture with CD70-HIT, CD70-1XX CAR, CD19-HIT or UTD T cells. Representative of three different donors. $CD19^{POS}$ B cells (B) $CD4^{POS}$ T cells (C) and $CD8^{POS}$ T cells (D). $n = 3$, mean ± SD, *P <0.05; **P <0.01; ****P <0.0001; one-way ANOVA. (**E**) Flow cytometric analysis of CD70 expression on human primary esophageal epithelial cells 18 hours after co-culture with CD70-HIT, CD70-1XX CAR, CD19-HIT and UTD T cells at an effector to target ratio of 4:1.

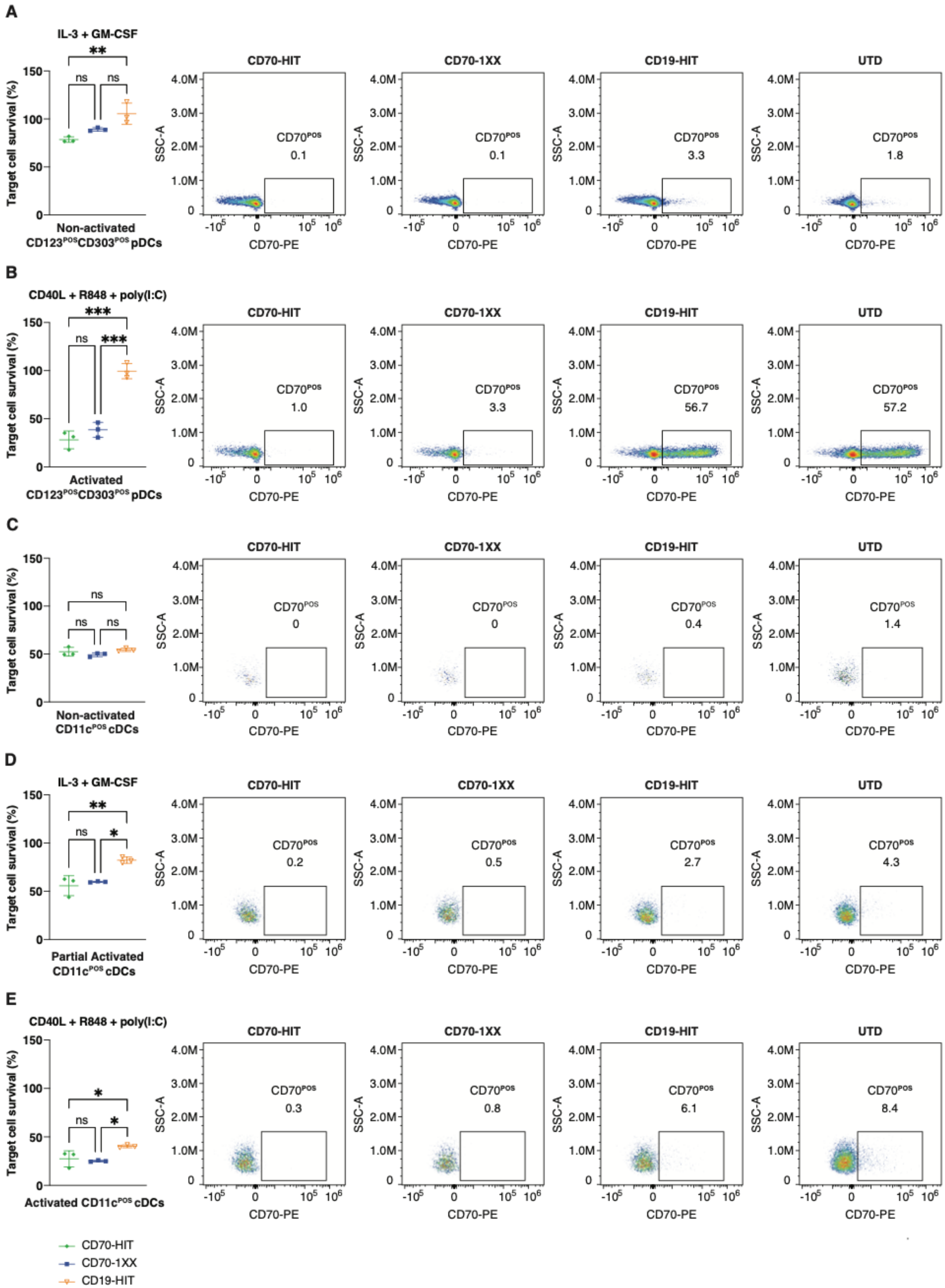

fig. S15. CD70-HIT in vitro cytotoxicity against non-activated and activated human dendritic cell subsets (related to Fig. 4).

The in vitro cytotoxicity of autologous CD70-HIT, CD70-CAR, CD19-HIT or untransduced (UTD) T cells against non-activated and activated dendritic cell subsets isolated from adult healthy donors at an effector to target ratio of 2:1. Donor 20 in (A) and (B); donor 21 in (C to E). Cytotoxicity and CD70 expression were evaluated by flow cytometry 18 hours after co-culture. On the left, target cell survival. Survival is relative to cells co-cultured with untransduced T cells. $n = 3$; mean ± SD; ns, not significant; $^{*}P < 0.05$; $^{**}P < 0.01$; $^{***}P < 0.001$; one-way ANOVA. On the right, flow cytometric plots of CD70 expression on indicated cell type 18 hours after co-culture with CD70-HIT, CD70-1XX CAR, CD19-HIT or UTD T cells. (**A**) Non-activated $CD123^{POS}CD303^{POS}$ plasmacytoid dendritic cells cultured with IL-3 + GM-CSF for 24 hours prior to the CTL. (**B**) Activated $CD123^{POS}CD303^{POS}$ plasmacytoid dendritic cells activated with CD40L + R848 + poly(I:C) for 24 hours prior to the CTL. (**C**) Non-activated $CD11c^{POS}$ conventional dendritic cells cultured with IL-3 for 24 hours prior to the CTL. (**D**) Partial activated $CD11c^{POS}$ conventional dendritic cells cultured with IL-3 + GM-CSF for 24 hours prior to the CTL. (**E**) Activated $CD11c^{POS}$ conventional dendritic cells activated with CD40L + R848 + poly(I:C) for 24 hours prior to the CTL.

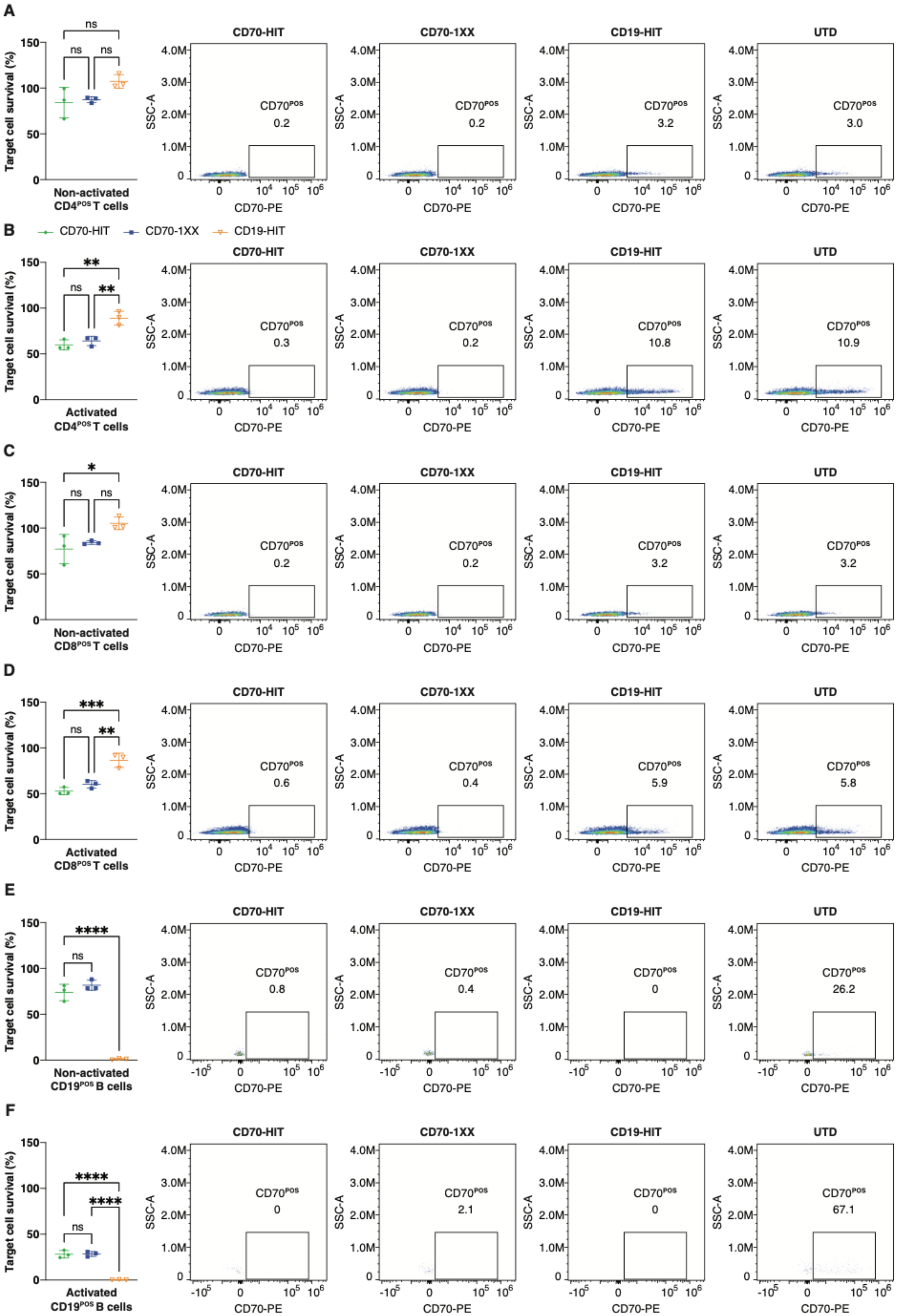
A
Target cell survival (%)
Non-activated CD4^POS T cells
ns
CD70-HIT
CD70-1XX
CD19-HIT
UTD
SSC-A
CD70-PE
CD70^POS 0.2
CD70^POS 0.2
CD70^POS 3.2
CD70^POS 3.0
B
CD70-HIT
CD70-1XX
CD19-HIT
Activated CD4^POS T cells
**
CD70^POS 0.3
CD70^POS 0.2
CD70^POS 10.8
CD70^POS 10.9
C
Non-activated CD8^POS T cells
*
CD70^POS 0.2
CD70^POS 0.2
CD70^POS 3.2
CD70^POS 3.2
D
Activated CD8^POS T cells
***
CD70^POS 0.6
CD70^POS 0.4
CD70^POS 5.9
CD70^POS 5.8
E
Non-activated CD19^POS B cells
****
CD70^POS 0.8
CD70^POS 0.4
CD70^POS 0
CD70^POS 26.2
F
Activated CD19^POS B cells
****
CD70^POS 0
CD70^POS 2.1
CD70^POS 0
CD70^POS 67.1

fig. S16. CD70-HIT in vitro cytotoxicity against non-activated and activated human T cells and B cells (related to Fig. 4).

The in vitro cytotoxicity of autologous CD70-HIT, CD70-CAR, CD19-HIT or untransduced (UTD) T cells against non-activated and activated human T cells and B cells isolated from adult healthy donors at an effector to target ratio of 2:1. Donor 20 in (A to D); donor 21 in (E) and (F). Cytotoxicity and CD70 expression were evaluated by flow cytometry 18 hours after co-culture. On the left, target cell survival. Survival is relative to cells co-cultured with untransduced T cells. $n = 3$; mean ± SD; ns, not significant; *P <0.05; **P <0.01; ***P <0.001; ****P <0.0001; one-way ANOVA. On the right, flow cytometric plots of CD70 expression on indicated cell type 18 hours after co-culture with CD70-HIT, CD70-1XX CAR, CD19-HIT or UTD T cells. (**A**) Non-activated $CD4^{POS}$ T cells (**B**) CD3/CD28 activated $CD4^{POS}$ T cells (**C**) Non-activated $CD8^{POS}$ T cells (**D**) CD3/CD28 activated $CD8^{POS}$ T cells (**E**) Non-activated $CD19^{POS}$ B cells (**F**) Activated $CD19^{POS}$ B cells.

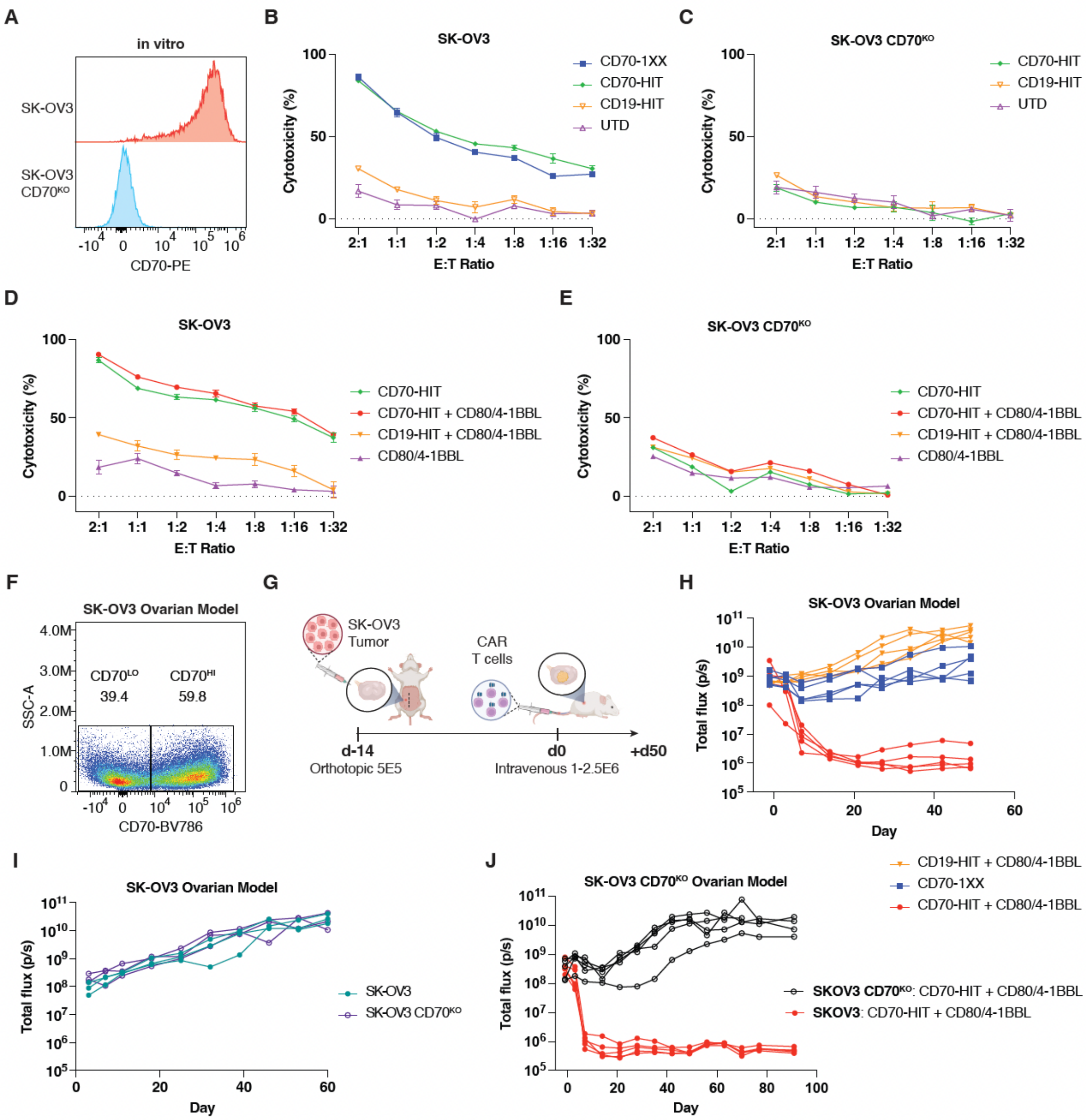

fig. S17. CD70-HIT T cells eradicate SK-OV3 ovarian tumor (related to Fig. 5).
(**A**) Flow cytometric analysis showing in vitro expression of CD70 on SK-OV3 and CRISPR-Cas9 CD70-knockout SK-OV3 (SK-OV3 $CD70^{KO}$). (**B**) Cytotoxicity assay using an 18-hour bioluminescence assay, using firefly luciferase expressing SK-OV3 as target cells, which were incubated at the indicated E:T ratios with CD70-1XX CAR, CD70-HIT, CD19-HIT or untransduced (UTD) T cells. $n = 3$, mean ± SEM. Donor 5; representative of three different donors. (**C**) Cytotoxicity assay using an 18-hour bioluminescence assay, using firefly luciferase expressing SK-OV3 $CD70^{KO}$ as target cells. $n = 3$, mean ± SEM. Donor 5; representative of three different donors. (**D** and **E**) Cytotoxicity assay using an 18-hour bioluminescence assay, using firefly luciferase expressing SK-OV3 (D) or SK-OV3 $CD70^{KO}$ (E) as target cells, which were incubated at the indicated E:T ratios with CD70-HIT, CD70-HIT+CD80/4-1BBL, CD19-HIT+CD80/4-1BBL or CD80/4-1BBL T cells (donor 23). $n = 3$, mean ± SEM. Representative of three different donors. (**F**) Representative flow cytometric analysis of CD70 expression on ex-

vivo SK-OV3 tumor, which had been orthotopically implanted into the ovary of NSG mice. (**G**) Diagram depicting in vivo CAR T treatment of SK-OV3 ovarian model. SK-OV3 ovarian cancer cell line is administered orthotopically (5×10^5 per mouse) into the ovary of NSG mice with tumor growth predominantly in the ovary. CAR T cell timing, dose and route of administration depicted. (**H**) Tumor burden (total flux) in mice bearing SK-OV3 at ovarian site and treated with 2.5×10^6 CD70-1XX CAR, CD70-HIT+CD80/4-1BBL or CD19-HIT+CD80/4-1BBL T cells (donor 24). n = 5 mice per group. Representative of two independent in vivo experiments (with two different donors). (**I**) Growth kinetics of SK-OV3 and SK-OV3 $CD70^{KO}$ tumor orthotopically implanted into ovary, measured by bioluminescence imaging (total flux is shown). (**J**) Tumor burden (total flux) in mice bearing SK-OV3 or SK-OV3 $CD70^{KO}$ tumor at ovarian site and treated with 2.5×10^6 CD70-HIT+CD80-41BBL (donor 25). n = 5 mice per group.

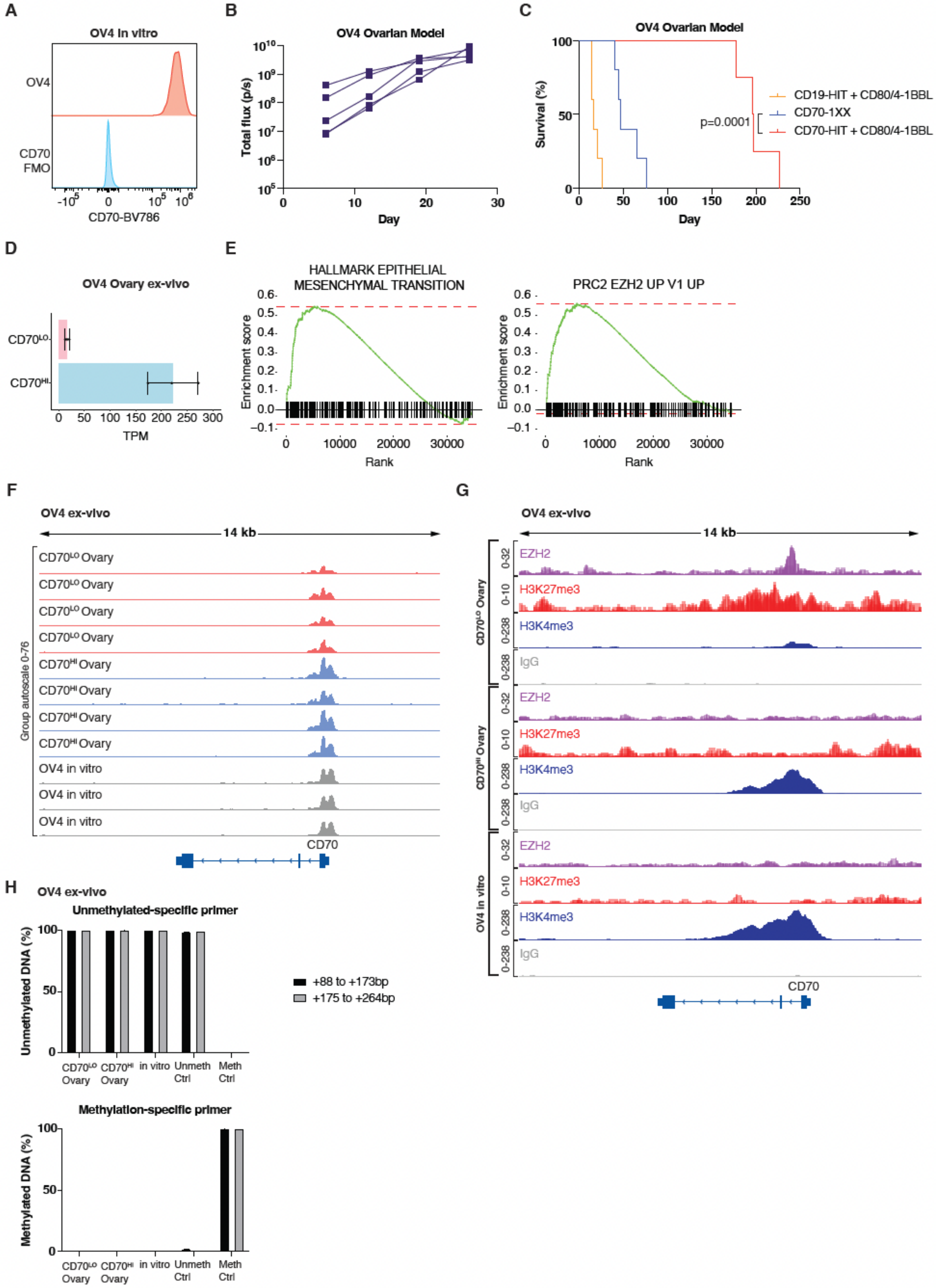

fig. S18. CD70 is epigenetically silenced by EZH2-mediated H3K27me3 to low levels in

OV4 high-grade serous ovarian carcinoma PDX tumor.

(**A**) Flow cytometric analysis showing in vitro expression of CD70 on OV4 high-grade serous ovarian carcinoma (HGSOC) PDX. (**B**) Growth kinetics of OV4 PDX tumor orthotopically implanted into the ovary, measured by bioluminescence imaging (BLI). Total flux is shown. (**C**) Kaplan-Meier survival analysis of mice bearing OV4 PDX tumor at ovarian site treated with 1.75×10^6 CD70-1XX CAR, CD70-HIT+CD80/4-1BBL or CD19-HIT+CD80/4-1BBL T cells (donor 21). $n = 5$ mice per group, P value determined by log-rank Mantel-Cox test (survival). (**D**) CD70 transcript expression in CD70^{LO} and CD70^{HI} OV4 tumor cells at the ovarian site. $n = 3$ mice per PDX, mean ± SD. (**E**) GSEA analysis of assay for transposase-accessible chromatin with sequencing (ATAC-seq) in ex-vivo CD70^{HI} versus CD70^{LO} OV4 tumor cells from the ovarian site revealed enrichment for Hallmark epithelial mesenchymal transition (EMT) pathway and C6 oncogenic PRC2 EZH2 UP V1 UP pathway (genes upregulated upon EZH2 knockdown) ($Padj$ <0.05). (**F**) Genome browser view of chromatin accessibility at the *CD70* gene in ex-vivo CD70^{LO} and CD70^{HI} OV4 tumor cells from the ovarian site as determined by ATAC-seq. Differential chromatin accessibility of *CD70* corresponds with differential CD70 expression. *n* 4 mice per condition. (**G**) Genome browser views of the *CD70* genomic region showing enrichment for EZH2 and repressive H3K27me3 histone modifications at the *CD70* locus in CD70^{LO} OV4 tumor cells from the ovary, and activating H3K4me3 histone modifications at the *CD70* locus in CD70^{HI} OV4 tumor cells from the ovary, assessed by Cleavage Under Targets & Release Using Nuclease (CUT&RUN). Overlay of 3 to 4 mice per condition. (**H**) Percentage of DNA methylated in the *CD70* promoter and 1st exon in genomic DNA isolated from ex-vivo CD70^{LO} and CD70^{HI} OV4 tumor cells from the ovarian site. Genomic DNA was subjected to bisulfite conversion and then digital droplet PCR using methylation-specific primers. Methylated control (Meth Ctrl) is genomic DNA from Jurkat cells enzymatically methylated with CpG methyltransferase. Unmethylated control (Unmeth Ctrl) is unmethylated DNA. $n = 3$ mice per PDX, mean ± SD.

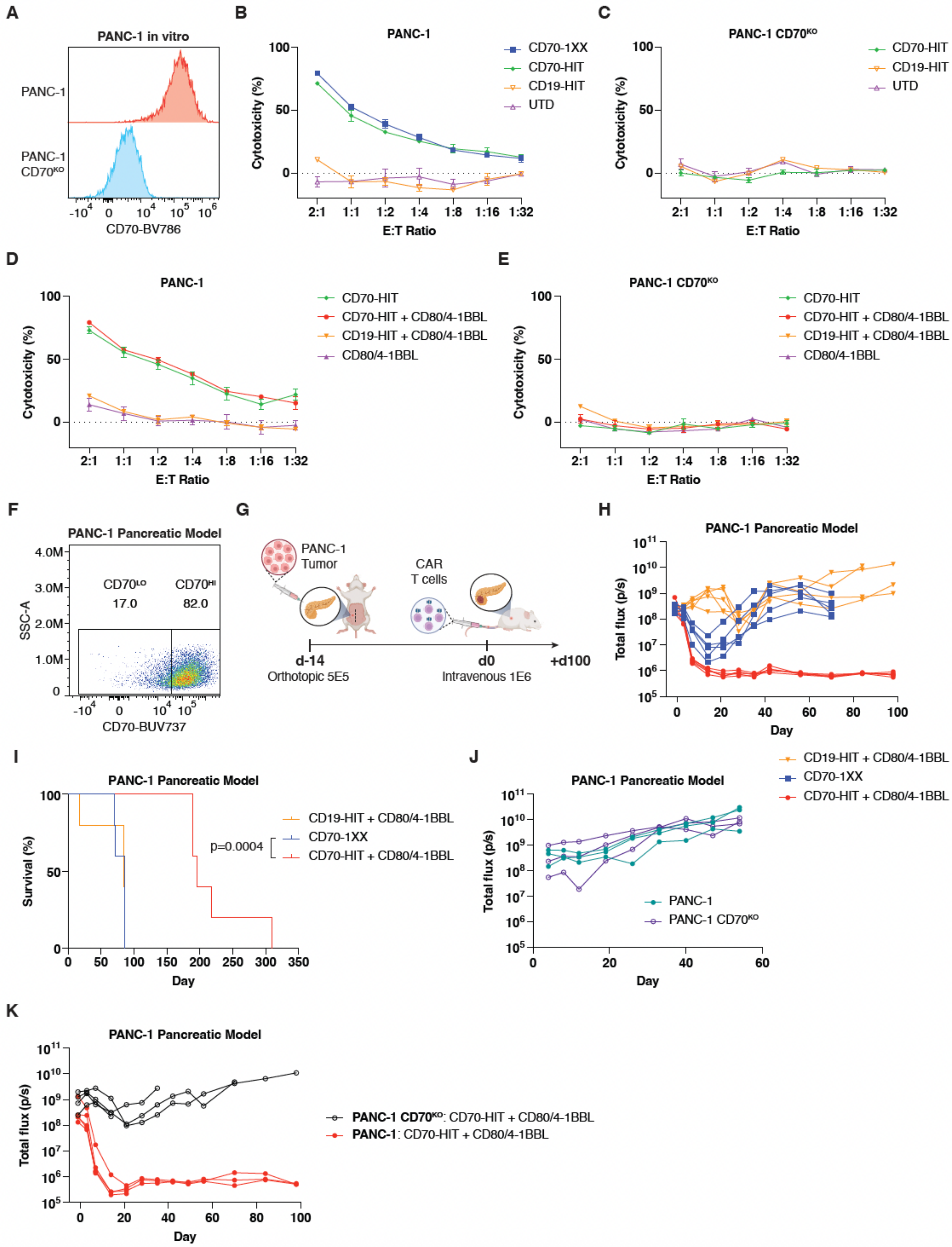

fig. S19. CD70-HIT T cells eradicate PANC-1 pancreatic tumor (related to Fig. 5). (**A**) Flow cytometric analysis showing in vitro expression of CD70 on PANC-1 and CRISPR-Cas9 CD70-knockout PANC-1, PANC-1CD70^{KO}. (**B**) Cytotoxicity assay using an 18-hour bioluminescence assay, using firefly luciferase expressing PANC-1 as target cells, which were

incubated at the indicated E:T ratios with CD70-1XX CAR, CD70-HIT, CD19-HIT or untransduced (UTD) T cells. *n* = 3, mean ± SEM. Donor 5; representative of three different donors. (**C**) Cytotoxicity assay using an 18-hour bioluminescence assay, using firefly luciferase expressing PANC-1 $CD70^{KO}$ as target cells. *n* = 3, mean ± SEM. Donor 5; representative of three different donors. (**D** and **E**) Cytotoxicity assay using an 18-hour bioluminescence assay, using firefly luciferase expressing PANC-1 (D) or PANC-1 $CD70^{KO}$ (E) as target cells, which were incubated at the indicated E:T ratios with CD70-HIT, CD70-HIT+CD80/4-1BBL, CD19-HIT+CD80/4-1BBL or CD80/4-1BBL T cells (donor 23). *n* = 3, mean ± SEM. Representative of three different donors. (**F**) Representative flow cytometric analysis of CD70 expression on ex-vivo PANC-1 tumor, which had been orthotopically implanted into the pancreas of NSG mice. (**G**) Diagram depicting in vivo CAR T treatment of PANC-1 pancreatic model. PANC-1 pancreatic cancer cell line is injected orthotopically (5×10^5 per mouse) into the pancreas of NSG mice with tumor growth in the pancreas. CAR T cell timing, dose and route of administration depicted. (**H**) Tumor burden (total flux) in mice bearing PANC-1 at pancreatic site and treated with 1×10^6 CD70-1XX CAR, CD70-HIT+CD80/4-1BBL or CD19-HIT+CD80/4-1BBL T cells (donor 26). *n* = 5 mice per group. Representative of three independent donors (with three different donors). (**I**) Kaplan-Meier survival analysis of PANC-1 pancreatic site-bearing mice treated with 1×10^6 CD70-1XX CAR, CD70-HIT+CD80/4-1BBL or CD19-HIT+CD80/4-1BBL T cells from an independent experiment (donor 27). *n* = 5 mice per group, *P* value determined by log-rank Mantel-Cox test (survival). (**J**) Growth kinetics of PANC-1 and PANC-1 $CD70^{KO}$ tumor orthotopically implanted into the pancreas, measured by bioluminescence imaging (BLI). Total flux is shown. (**K**) Tumor burden (total flux) in mice bearing PANC-1 or PANC-1 $CD70^{KO}$ tumor at pancreatic site and treated with 2.5×10^6 CD70-HIT+CD80-41BBL (donor 28). *n* = 5 mice per group.

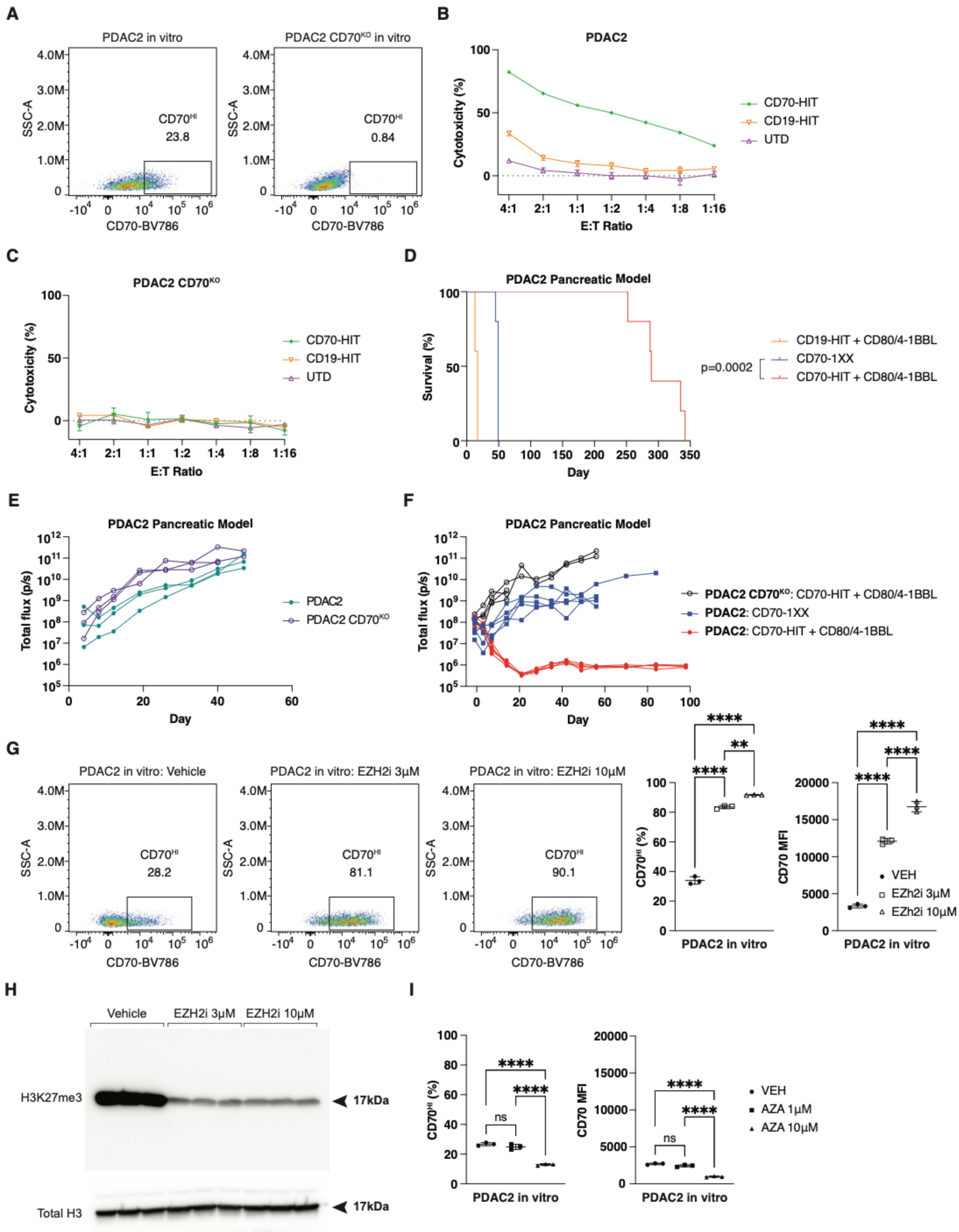

fig. S20. CD70-HIT T cells eradicate PDAC2 pancreatic ductal adenocarcinoma (PDAC) PDX (related to Fig. 5).

(**A**) Flow cytometric analysis showing in vitro CD70 expression on PDAC2 or CRISPR-Cas9 CD70-knockout PDAC2, PDAC2 $CD70^{KO}$. CD70 expression in PDAC2 is heterogeneous in vitro. (**B** and **C**) Cytotoxicity assay using an 18-hour bioluminescence assay, using firefly luciferase expressing PDAC2 (B) or PDAC2 $CD70^{KO}$ (C) as target cells, which were incubated at the indicated E:T ratios with CD70-HIT, CD19-HIT or untransduced (UTD) T cells. $n = 3$, mean ± SEM. Donor 5; representative of three different donors. (**D**) Kaplan-Meier survival analysis of PDAC2 pancreatic site-bearing mice treated with 2.5×10^6 HIT or CAR T cells (donor 17). $n = 5$ mice per group, *P* value determined by log-rank Mantel-Cox test (survival). (**E**) Growth kinetics of PDAC2 and PDAC2 $CD70^{KO}$ tumor orthotopically implanted into the pancreas, measured by bioluminescence imaging (BLI). Total flux is shown. (**F**) Tumor burden (total flux) in mice bearing PDAC2 or PDAC2 $CD70^{KO}$ at pancreatic site and treated with 2.5×10^6 HIT or CAR T cells (donor 28). $n = 5$ mice per group. Representative of two independent in vivo experiments (with two different donors). (**G**) Flow cytometric analysis of percentage of CD70 expression, and CD70 mean fluorescence intensity (MFI) on PDAC2 PDX in vitro cells treated with either DMSO (vehicle) or tazemetostat (EZH2 inhibitor, EZH2i 3µM or EZH2i 10µM) for 8 days in culture. $n = 3$, mean ± SD, $^{**}P < 0.01$, $^{****}P < 0.0001$, one-way ANOVA. (**H**) H3K27me3 and total Histone H3 were measured by Western blot for in vitro PDAC2 cells treated with vehicle, EZH2i 3µM or EZH2i 10µM in (G). (**I**) Flow cytometric analysis of percentage of CD70 expression, and CD70 mean fluorescence intensity (MFI) expression on PDAC2 PDX in vitro cells treated with either DMSO (vehicle) or azacytidine (AZA, AZA 1µM or AZA 10µM) for 3 days in culture. $n = 3$; mean ± SD; ns, not significant; $^{****}P < 0.0001$, one-way ANOVA.

References and Notes

1. M. Sadelain, I. Rivière, S. Riddell, Therapeutic T cell engineering. *Nature* **545**, 423–431 (2017). doi:10.1038/nature22395 Medline

2. C. H. June, M. Sadelain, Chimeric Antigen Receptor Therapy. *N. Engl. J. Med.* **379**, 64–73 (2018). doi:10.1056/NEJMra1706169 Medline

3. J. Wagner, E. Wickman, C. DeRenzo, S. Gottschalk, CAR T Cell Therapy for Solid Tumors: Bright Future or Dark Reality? *Mol. Ther.* **28**, 2320–2339 (2020). doi:10.1016/j.ymthe.2020.09.015 Medline

4. R. A. Morgan, J. C. Yang, M. Kitano, M. E. Dudley, C. M. Laurencot, S. A. Rosenberg, Case report of a serious adverse event following the administration of T cells transduced with a chimeric antigen receptor recognizing ERBB2. *Mol. Ther.* **18**, 843–851 (2010). doi:10.1038/mt.2010.24 Medline

5. C. H. Lamers, S. Sleijfer, S. van Steenbergen, P. van Elzakker, B. van Krimpen, C. Groot, A. Vulto, M. den Bakker, E. Oosterwijk, R. Debets, J. W. Gratama, Treatment of metastatic renal cell carcinoma with CAIX CAR-engineered T cells: Clinical evaluation and management of on-target toxicity. *Mol. Ther.* **21**, 904–912 (2013). doi:10.1038/mt.2013.17 Medline

6. F. C. Thistlethwaite, D. E. Gilham, R. D. Guest, D. G. Rothwell, M. Pillai, D. J. Burt, A. J. Byatte, N. Kirillova, J. W. Valle, S. K. Sharma, K. A. Chester, N. B. Westwood, S. E. R. Halford, S. Nabarro, S. Wan, E. Austin, R. E. Hawkins, The clinical efficacy of first-generation carcinoembryonic antigen (CEACAM5)-specific CAR T cells is limited by poor persistence and transient pre-conditioning-dependent respiratory toxicity. *Cancer Immunol. Immunother.* **66**, 1425–1436 (2017). doi:10.1007/s00262-017-2034-7 Medline

7. M. R. Bowman, M. A. Crimmins, J. Yetz-Aldape, R. Kriz, K. Kelleher, S. Herrmann, The cloning of CD70 and its identification as the ligand for CD27. *J. Immunol.* **152**, 1756–1761 (1994). doi:10.4049/jimmunol.152.4.1756 Medline

8. R. Q. Hintzen, S. M. Lens, M. P. Beckmann, R. G. Goodwin, D. Lynch, R. A. van Lier, Characterization of the human CD27 ligand, a novel member of the TNF gene family. *J. Immunol.* **152**, 1762–1773 (1994). doi:10.4049/jimmunol.152.4.1762 Medline

9. J. Diegmann, K. Junker, B. Gerstmayer, A. Bosio, W. Hindermann, J. Rosenhahn, F. von Eggeling, Identification of CD70 as a diagnostic biomarker for clear cell renal cell carcinoma by gene expression profiling, real-time RT-PCR and immunohistochemistry. *Eur. J. Cancer* **41**, 1794–1801 (2005). doi:10.1016/j.ejca.2005.05.005 Medline

10. K. Junker, W. Hindermann, F. von Eggeling, J. Diegmann, K. Haessler, J. Schubert, CD70: A new tumor specific biomarker for renal cell carcinoma. *J. Urol.* **173**, 2150–2153 (2005). doi:10.1097/01.ju.0000158121.49085.ba Medline

11. L. Jin, H. Ge, Y. Long, C. Yang, Y. E. Chang, L. Mu, E. J. Sayour, G. De Leon, Q. J. Wang, J. C. Yang, P. S. Kubilis, H. Bao, S. Xia, D. Lu, Y. Kong, L. Hu, Y. Shang, C. Jiang, J. Nie, S. Li, Y. Gu, J. Sun, D. A. Mitchell, Z. Lin, J. Huang, CD70, a novel target of CAR T-cell therapy for gliomas. *Neuro Oncol.* **20**, 55–65 (2018). doi:10.1093/neuonc/nox116 Medline

12. M. Seyfrid, W. T. Maich, V. M. Shaikh, N. Tatari, D. Upreti, D. Piyasena, M. Subapanditha, N. Savage, D. McKenna, N. Mikolajewicz, H. Han, C. Chokshi, L. Kuhlmann, A. Khoo, S. K. Salim, B. Archibong-Bassey, W. Gwynne, K. Brown, N. Murtaza, D. Bakhshinyan, P. Vora, C. Venugopal, J. Moffat, T. Kislinger, S. Singh, CD70 as an actionable immunotherapeutic target in recurrent glioblastoma and its microenvironment. *J. Immunother. Cancer* **10**, e003289 (2022). doi:10.1136/jitc-2021-003289 Medline

13. M. C. Ryan, H. Kostner, K. A. Gordon, S. Duniho, M. K. Sutherland, C. Yu, K. M. Kim, A. Nesterova, M. Anderson, J. A. McEarchern, C.-L. Law, L. M. Smith, Targeting pancreatic and ovarian carcinomas using the auristatin-based anti-CD70 antibody-drug conjugate SGN-75. *Br. J. Cancer* **103**, 676–684 (2010). doi:10.1038/sj.bjc.6605816 Medline

14. K. Nakamura, M. Sho, T. Akahori, S. Nishiwada, T. Kunishige, K. Nakagawa, M. Nagai, T. Takagi, T. Terai, N. Ikeda, Clinical relevance of CD70 expression in resected pancreatic cancer: Prognostic value and therapeutic potential. *Pancreatology* **21**, 573–580 (2021). doi:10.1016/j.pan.2021.01.013 Medline

15. M. Shiomi, S. Matsuzaki, S. Serada, K. Matsuo, C. Mizuta-Odani, M. Jitsumori, R. Nakae, S. Matsuzaki, S. Nakagawa, K. Hiramatsu, A. Miyoshi, E. Kobayashi, T. Kimura, Y. Ueda, K. Yoshino, T. Naka, T. Kimura, CD70 antibody-drug conjugate: A potential novel therapeutic agent for ovarian cancer. *Cancer Sci.* **112**, 3655–3668 (2021). doi:10.1111/cas.15027 Medline

16. S. M. A. Lens, R. de Jong, B. Hooibrink, G. Koopman, S. T. Pals, M. H. J. van Oers, R. A. W. van Lier, Phenotype and function of human B cells expressing CD70 (CD27 ligand). *Eur. J. Immunol.* **26**, 2964–2971 (1996). doi:10.1002/eji.1830261223 Medline

17. M. T. M. Vossen, M. Matmati, K. M. L. Hertoghs, P. A. Baars, M.-R. Gent, G. Leclercq, J. Hamann, T. W. Kuijpers, R. A. W. van Lier, CD27 defines phenotypically and functionally different human NK cell subsets. *J. Immunol.* **180**, 3739–3745 (2008). doi:10.4049/jimmunol.180.6.3739 Medline

18. M. Hashimoto-Okada, T. Kitawaki, N. Kadowaki, S. Iwata, C. Morimoto, T. Hori, T. Uchiyama, The CD70-CD27 interaction during the stimulation with dendritic cells promotes naive CD4(+) T cells to develop into T cells producing a broad array of immunostimulatory cytokines in humans. *Int. Immunol.* **21**, 891–904 (2009). doi:10.1093/intimm/dxp056 Medline

19. T. Flieswasser, V. Camara-Clayette, A. Danu, J. Bosq, V. Ribrag, P. Zabrocki, L. Van Rompaey, H. de Haard, K. Zwaenepoel, E. Smits, P. Pauwels, J. Jacobs, Screening a Broad Range of Solid and Haematological Tumour Types for CD70 Expression Using a Uniform IHC Methodology as Potential Patient Stratification Method. *Cancers* **11**, 1611 (2019). doi:10.3390/cancers11101611 Medline

20. Y. P. Park, L. Jin, K. B. Bennett, D. Wang, K. M. Fredenburg, J. E. Tseng, L.-J. Chang, J. Huang, E. K. L. Chan, CD70 as a target for chimeric antigen receptor T cells in head and neck squamous cell carcinoma. *Oral Oncol.* **78**, 145–150 (2018). doi:10.1016/j.oraloncology.2018.01.024 Medline

21. H. Ye, R. R. Huang, B. M. Shuch, Z. Chen, J. W. Said, A. J. Pantuck, S. Panowski, CD70 is a promising CAR-T cell target in patients with advanced renal cell carcinoma. *J. Clin. Oncol.* **40**, 384–384 (2022). doi:10.1200/JCO.2022.40.6_suppl.384

22. R. R. Huang, Z. Chen, N. Kroeger, A. Pantuck, J. Said, H. M. Kluger, B. Shuch, H. Ye, CD70 is Consistently Expressed in Primary and Metastatic Clear Cell Renal Cell Carcinoma. *Clin. Genitourin. Cancer* **22**, 347–353 (2023). doi:10.1016/j.clgc.2023.12.003 Medline

23. S. Srour, R. Kotecha, B. Curti, J. Chahoud, A. Drakaki, L. Tang, L. Goyal, S. Prashad, V. Szenes, K. Norwood, S. Pal, Abstract CT011: A phase 1 multicenter study (TRAVERSE) evaluating the safety and efficacy of ALLO-316 following conditioning regimen in pts with advanced or metastatic clear cell renal cell carcinoma (ccRCC). *Cancer Res.* **83**, CT011 (2023). doi:10.1158/1538-7445.AM2023-CT011

24. S. Pal, B. Tran, J. Haanen, M. Hurwitz, A. Sacher, N. Agarwal, N. Tannir, E. Budde, S. Harrison, S. Klobuch, S. Patel, M. L. Dequeant, V. Karsten, K. Cohen, E. Gurary, H. Dar, A. Ma, A. Sharma, S. Srour, 558 CTX130 allogeneic CRISPR-Cas9–engineered chimeric antigen receptor (CAR) T cells in patients with advanced clear cell renal cell carcinoma: Results from the phase 1 COBALT-RCC study. *J. Immunother. Cancer* **10** (suppl. 2), A584–A584 (2022). doi:10.1136/jitc-2022-SITC2022.0558

25. S. K. Pal, B. Tran, J. B. A. G. Haanen, M. E. Hurwitz, A. Sacher, N. M. Tannir, L. E. Budde, S. J. Harrison, S. Klobuch, S. S. Patel, L. Meza, M.-L. Dequeant, A. Ma, Q. A. He, L. M. Williams, A. Keegan, E. B. Gurary, H. Dar, S. Karnik, C. Guo, H. Heath, R. R. Yuen, P. K. Morrow, N. Agarwal, S. A. Srour, CD70-Targeted Allogeneic CAR T-Cell Therapy for Advanced Clear Cell Renal Cell Carcinoma. *Cancer Discov.* **14**, 1176–1189 (2024). doi:10.1158/2159-8290.CD-24-0102 Medline

26. J. Mansilla-Soto, J. Eyquem, S. Haubner, M. Hamieh, J. Feucht, N. Paillon, A. E. Zucchetti, Z. Li, M. Sjöstrand, P. L. Lindenbergh, M. Saetersmoen, A. Dobrin, M. Maurin, A. Iyer, A. Garcia Angus, M. M. Miele, Z. Zhao, T. Giavridis, S. J. C. van der Stegen, F. Tamzalit, I. Rivière, M. Huse, R. C. Hendrickson, C. Hivroz, M. Sadelain, HLA-independent T cell receptors for targeting tumors with low antigen density. *Nat. Med.* **28**, 345–352 (2022). doi:10.1038/s41591-021-01621-1 Medline

27. M. Hamieh, J. Mansilla-Soto, I. Rivière, M. Sadelain, Programming CAR T Cell Tumor Recognition: Tuned Antigen Sensing and Logic Gating. *Cancer Discov.* **13**, 829–843 (2023). doi:10.1158/2159-8290.CD-23-0101 Medline

28. Y. Liu, G. Liu, J. Wang, Z.-Y. Zheng, L. Jia, W. Rui, D. Huang, Z.-X. Zhou, L. Zhou, X. Wu, S. Lin, X. Zhao, X. Lin, Chimeric STAR receptors using TCR machinery mediate robust responses against solid tumors. *Sci. Transl. Med.* **13**, eabb5191 (2021). doi:10.1126/scitranslmed.abb5191 Medline

29. M. T. Stephan, V. Ponomarev, R. J. Brentjens, A. H. Chang, K. V. Dobrenkov, G. Heller, M. Sadelain, T cell-encoded CD80 and 4-1BBL induce auto- and transcostimulation, resulting in potent tumor rejection. *Nat. Med.* **13**, 1440–1449 (2007). doi:10.1038/nm1676 Medline

30. Z. Zhao, M. Condomines, S. J. C. van der Stegen, F. Perna, C. C. Kloss, G. Gunset, J. Plotkin, M. Sadelain, Structural Design of Engineered Costimulation Determines Tumor

Rejection Kinetics and Persistence of CAR T Cells. *Cancer Cell* **28**, 415–428 (2015). doi:10.1016/j.ccell.2015.09.004 Medline

31. K. Zhang, J. D. Hocker, M. Miller, X. Hou, J. Chiou, O. B. Poirion, Y. Qiu, Y. E. Li, K. J. Gaulton, A. Wang, S. Preissl, B. Ren, A single-cell atlas of chromatin accessibility in the human genome. *Cell* **184**, 5985–6001.e19 (2021). doi:10.1016/j.cell.2021.10.024 Medline

32. J. M. Granja, M. R. Corces, S. E. Pierce, S. T. Bagdatli, H. Choudhry, H. Y. Chang, W. J. Greenleaf, ArchR is a scalable software package for integrative single-cell chromatin accessibility analysis. *Nat. Genet.* **53**, 403–411 (2021). doi:10.1038/s41588-021-00790-6 Medline

33. J. H. Park, M. L. Palomba, K. Perica, S. M. Devlin, G. Shah, P. B. Dahi, R. J. Lin, G. Salles, M. Scordo, K. Nath, Y. K. Valtis, A. Lynch, E. Cathcart, H. Zhang, H. Schoder, D. Leithner, K. Liotta, A. Yu, K. Stocker, J. Li, A. Dey, L. Sellner, R. Singh, V. Sundaresan, X. Tong, F. Zhao, J. Mansilla-Soto, C. He, J. Meyerson, K. Hosszu, D. McAvoy, X. Wang, I. Rivière, M. Sadelain, Results From First-in-Human Phase I Study of a Novel CD19-1XX Chimeric Antigen Receptor With Calibrated Signaling in Large B-Cell Lymphoma. *J. Clin. Oncol.* **43**, 2418–2428 (2025). doi:10.1200/JCO-24-02424 Medline

34. Y. B. Schwartz, T. G. Kahn, D. A. Nix, X.-Y. Li, R. Bourgon, M. Biggin, V. Pirrotta, Genome-wide analysis of Polycomb targets in Drosophila melanogaster. *Nat. Genet.* **38**, 700–705 (2006). doi:10.1038/ng1817 Medline

35. A. H. Juan, S. Wang, K. D. Ko, H. Zare, P.-F. Tsai, X. Feng, K. O. Vivanco, A. M. Ascoli, G. Gutierrez-Cruz, J. Krebs, S. Sidoli, A. L. Knight, R. A. Pedersen, B. A. Garcia, R. Casellas, J. Zou, V. Sartorelli, Roles of H3K27me2 and H3K27me3 Examined during Fate Specification of Embryonic Stem Cells. *Cell Rep.* **17**, 1369–1382 (2016). doi:10.1016/j.celrep.2016.09.087 Medline

36. P. Dobrinić, A. T. Szczurek, R. J. Klose, PRC1 drives Polycomb-mediated gene repression by controlling transcription initiation and burst frequency. *Nat. Struct. Mol. Biol.* **28**, 811–824 (2021). doi:10.1038/s41594-021-00661-y Medline

37. C. Riether, T. Pabst, S. Höpner, U. Bacher, M. Hinterbrandner, Y. Banz, R. Müller, M. G. Manz, W. H. Gharib, D. Francisco, R. Bruggmann, L. van Rompaey, M. Moshir, T. Delahaye, D. Gandini, E. Erzeel, A. Hultberg, S. Fung, H. de Haard, N. Leupin, A. F. Ochsenbein, Targeting CD70 with cusatuzumab eliminates acute myeloid leukemia stem cells in patients treated with hypomethylating agents. *Nat. Med.* **26**, 1459–1467 (2020). doi:10.1038/s41591-020-0910-8 Medline

38. C. Kasap, A. Izgutdina, B. Patiño-Escobar, A. S. Kang, N. Chilakapati, N. Akagi, A. Manoj, H. Johnson, T. Rashid, J. Werner, A. Barpanda, H. Geng, Y. T. Lin, S. Rampersaud, D. Gil-Alós, A. Sobh, D. Dupéré-Richer, A. Aleman, G. Wicaksono, K. M. K. Kelii, R. Dalal, E. Ramos, A. Vijayanarayanan, K. Lakhani, F. Salangsang, P. Phojanakong, J. A. Camara Serrano, O. Zakraoui, I. Tariq, A. Chari, A. Chung, A. D. Kumar, T. G. Martin 3rd, J. Wolf, S. W. Wong, V. Steri, M. Shanmugam, L. H. Boise, T. Kortemme, S. Parekh, E. Stieglitz, J. D. Licht, W. J. Karlon, B. G. Barwick, A. P. Wiita, Targeting high-risk multiple myeloma genotypes with optimized anti-CD70 CAR T cells. *Blood* **146**, 819–833 (2025). doi:10.1182/blood.2024025536 Medline

39. S. A. Srour, J. Chahoud, A. Drakaki, B. D. Curti, S. Pal, L. Tang, S. Prashad, J. Atwell, C. Williams, S. Ghatta, C. J. Severyn, J. L. Gall, N. M. Tannir, R. R. Kotecha, 322 ALLO-316 in patients with advanced or metastatic clear cell renal cell carcinoma (ccRCC): Updated safety and efficacy from the phase 1 TRAVERSE multicenter study. *J. Immunother. Cancer* **12** (suppl. 2), A373–A373 (2024). doi:10.1136/jitc-2024-SITC2024.0322

40. S. H. Panowski, S. Srinivasan, N. Tan, S. K. Tacheva-Grigorova, B. Smith, Y. S. L. Mak, H. Ning, J. Villanueva, D. Wijewarnasuriya, S. Lang, Z. Melton, A. Ghosh, M. Dusseaux, R. Galetto, J. R. Heyen, T. Sai, T. Van Blarcom, J. Chaparro-Riggers, B. J. Sasu, Preclinical Development and Evaluation of Allogeneic CAR T Cells Targeting CD70 for the Treatment of Renal Cell Carcinoma. *Cancer Res.* **82**, 2610–2624 (2022). doi:10.1158/0008-5472.CAN-21-2931 Medline

41. Q. Xiong, H. Wang, Q. Shen, Y. Wang, X. Yuan, G. Lin, P. Jiang, The development of chimeric antigen receptor T-cells against CD70 for renal cell carcinoma treatment. *J. Transl. Med.* **22**, 368 (2024). doi:10.1186/s12967-024-05101-1 Medline

42. S. A. Srour, J. Chahoud, A. Drakaki, B. D. Curti, G. T. Gibney, S. K. Pal, L. Tang, S. Charmsaz, J. Atwell, P. B. Robbins, C. Williams, S. Ghatta, C. Severyn, J. Le Gall, N. M. Tannir, R. R. Kotecha, ALLO-316 in advanced clear cell renal cell carcinoma (ccRCC): Updated results from the phase 1 TRAVERSE study. *J. Clin. Oncol.* **43**, 4508–4508 (2025). doi:10.1200/JCO.2025.43.16_suppl.4508

43. J. H. Pahl, S. J. Santos, M. L. Kuijjer, G. H. Boerman, L. G. Sand, K. Szuhai, A. Cleton-Jansen, R. M. Egeler, J. V. Boveé, M. W. Schilham, A. C. Lankester, Expression of the immune regulation antigen CD70 in osteosarcoma. *Cancer Cell Int.* **15**, 31 (2015). doi:10.1186/s12935-015-0181-5 Medline

44. E. Rav, S. Maegawa, V. Gopalakrishnan, N. Gordon, Overview of CD70 as a Potential Therapeutic Target for Osteosarcoma. *J. Immunol.* **211**, 1067–1072 (2023). doi:10.4049/jimmunol.2200591 Medline

45. L. Liu, B. Yin, Z. Yi, X. Liu, Z. Hu, W. Gao, H. Yu, Q. Li, Breast cancer stem cells characterized by CD70 expression preferentially metastasize to the lungs. *Breast Cancer* **25**, 706–716 (2018). doi:10.1007/s12282-018-0880-6 Medline

46. S. Ortiz-Cuaran, A. Swalduz, J.-P. Foy, S. Marteau, A.-P. Morel, F. Fauvet, G. De Souza, L. Michon, M. Boussageon, N. Gadot, M. Godefroy, S. Léon, A. Tortereau, N.-E.-H. Mourksi, C. Leonce, M. A. Albaret, A. Dongre, B. Vanbervliet, M. Robert, L. Tonon, R. M. Pommier, V. Hofman, V. Attignon, S. Boyault, C. Audoynaud, J. Auclair, F. Bouquet, Q. Wang, C. Ménétrier-Caux, M. Pérol, C. Caux, P. Hofman, S. Lantuejoul, A. Puisieux, P. Saintigny, Epithelial-to-mesenchymal transition promotes immune escape by inducing CD70 in non-small cell lung cancer. *Eur. J. Cancer* **169**, 106–122 (2022). doi:10.1016/j.ejca.2022.03.038 Medline

47. M. B. Nilsson, Y. Yang, S. Heeke, S. A. Patel, A. Poteete, H. Udagawa, Y. Y. Elamin, C. A. Moran, Y. Kashima, T. Arumugam, X. Yu, X. Ren, L. Diao, L. Shen, Q. Wang, M. Zhang, J. P. Robichaux, C. Shi, A. N. Pfeil, H. Tran, D. L. Gibbons, J. Bock, J. Wang, J. D. Minna, S. S. Kobayashi, X. Le, J. V. Heymach, CD70 is a therapeutic target

upregulated in EMT-associated EGFR tyrosine kinase inhibitor resistance. *Cancer Cell* **41**, 340–355.e6 (2023). doi:10.1016/j.ccell.2023.01.007 Medline

48. J. Eyquem, J. Mansilla-Soto, T. Giavridis, S. J. C. van der Stegen, M. Hamieh, K. M. Cunanan, A. Odak, M. Gönen, M. Sadelain, Targeting a CAR to the TRAC locus with CRISPR/Cas9 enhances tumour rejection. *Nature* **543**, 113–117 (2017). doi:10.1038/nature21405 Medline

49. I. Rivière, K. Brose, R. C. Mulligan, Effects of retroviral vector design on expression of human adenosine deaminase in murine bone marrow transplant recipients engrafted with genetically modified cells. *Proc. Natl. Acad. Sci. U.S.A.* **92**, 6733–6737 (1995). doi:10.1073/pnas.92.15.6733 Medline

50. M. C. Gong, J.-B. Latouche, A. Krause, W. D. W. Heston, N. H. Bander, M. Sadelain, Cancer patient T cells genetically targeted to prostate-specific membrane antigen specifically lyse prostate cancer cells and release cytokines in response to prostate-specific membrane antigen. *Neoplasia* **1**, 123–127 (1999). doi:10.1038/sj.neo.7900018 Medline

51. E. P. Papapetrou, G. Lee, N. Malani, M. Setty, I. Riviere, L. M. S. Tirunagari, K. Kadota, S. L. Roth, P. Giardina, A. Viale, C. Leslie, F. D. Bushman, L. Studer, M. Sadelain, Genomic safe harbors permit high β-globin transgene expression in thalassemia induced pluripotent stem cells. *Nat. Biotechnol.* **29**, 73–78 (2011). doi:10.1038/nbt.1717 Medline

52. R. J. Brentjens, E. Santos, Y. Nikhamin, R. Yeh, M. Matsushita, K. La Perle, A. Quintás-Cardama, S. M. Larson, M. Sadelain, Genetically targeted T cells eradicate systemic acute lymphoblastic leukemia xenografts. *Clin. Cancer Res.* **13**, 5426–5435 (2007). doi:10.1158/1078-0432.CCR-07-0674 Medline

53. J. Schindelin, I. Arganda-Carreras, E. Frise, V. Kaynig, M. Longair, T. Pietzsch, S. Preibisch, C. Rueden, S. Saalfeld, B. Schmid, J.-Y. Tinevez, D. J. White, V. Hartenstein, K. Eliceiri, P. Tomancak, A. Cardona, Fiji: An open-source platform for biological-image analysis. *Nat. Methods* **9**, 676–682 (2012). doi:10.1038/nmeth.2019 Medline

54. N. Ansari, S. Müller, E. H. K. Stelzer, F. Pampaloni, "Quantitative 3D cell-based assay performed with cellular spheroids and fluorescence microscopy" in *Methods in Cell Biology*, P. M. Conn, Ed. (Academic Press, 2013), vol. 113 of *Laboratory Methods in Cell Biology*, chap. 13, pp. 295–309.

55. M. Stoeckius, S. Zheng, B. Houck-Loomis, S. Hao, B. Z. Yeung, W. M. Mauck 3rd, P. Smibert, R. Satija, Cell Hashing with barcoded antibodies enables multiplexing and doublet detection for single cell genomics. *Genome Biol.* **19**, 224 (2018). doi:10.1186/s13059-018-1603-1 Medline

56. Y. Hao, T. Stuart, M. H. Kowalski, S. Choudhary, P. Hoffman, A. Hartman, A. Srivastava, G. Molla, S. Madad, C. Fernandez-Granda, R. Satija, Dictionary learning for integrative, multimodal and scalable single-cell analysis. *Nat. Biotechnol.* **42**, 293–304 (2024). doi:10.1038/s41587-023-01767-y Medline

57. T. Sainburg, L. McInnes, T. Q. Gentner, Parametric UMAP embeddings for representation and semi-supervised learning. arXiv:2009.12981 [cs.LG] (2021).

58. M. R. Corces, A. E. Trevino, E. G. Hamilton, P. G. Greenside, N. A. Sinnott-Armstrong, S. Vesuna, A. T. Satpathy, A. J. Rubin, K. S. Montine, B. Wu, A. Kathiria, S. W. Cho, M. R. Mumbach, A. C. Carter, M. Kasowski, L. A. Orloff, V. I. Risca, A. Kundaje, P. A. Khavari, T. J. Montine, W. J. Greenleaf, H. Y. Chang, An improved ATAC-seq protocol reduces background and enables interrogation of frozen tissues. *Nat. Methods* **14**, 959–962 (2017). doi:10.1038/nmeth.4396 Medline

59. B. Langmead, S. L. Salzberg, Fast gapped-read alignment with Bowtie 2. *Nat. Methods* **9**, 357–359 (2012). doi:10.1038/nmeth.1923 Medline

60. Y. Zhang, T. Liu, C. A. Meyer, J. Eeckhoute, D. S. Johnson, B. E. Bernstein, C. Nusbaum, R. M. Myers, M. Brown, W. Li, X. S. Liu, Model-based analysis of ChIP-Seq (MACS). *Genome Biol.* **9**, R137 (2008). doi:10.1186/gb-2008-9-9-r137 Medline

61. Q. Li, J. B. Brown, H. Huang, P. J. Bickel, Measuring reproducibility of high-throughput experiments. *Ann. Appl. Stat.* **5**, 1752–1779 (2011). doi:10.1214/11-AOAS466

62. Y. Liao, G. K. Smyth, W. Shi, The R package Rsubread is easier, faster, cheaper and better for alignment and quantification of RNA sequencing reads. *Nucleic Acids Res.* **47**, e47 (2019). doi:10.1093/nar/gkz114 Medline

63. M. I. Love, W. Huber, S. Anders, Moderated estimation of fold change and dispersion for RNA-seq data with DESeq2. *Genome Biol.* **15**, 550 (2014). doi:10.1186/s13059-014-0550-8 Medline

64. G. Korotkevich, V. Sukhov, N. Budin, B. Shpak, M. N. Artyomov, A. Sergushichev, Fast gene set enrichment analysis. bioRxiv [Preprint] (2021). https://doi.org/10.1101/060012.

65. G. Yu, L.-G. Wang, Q.-Y. He, ChIPseeker: An R/Bioconductor package for ChIP peak annotation, comparison and visualization. *Bioinformatics* **31**, 2382–2383 (2015). doi:10.1093/bioinformatics/btv145 Medline

66. A. Dobin, C. A. Davis, F. Schlesinger, J. Drenkow, C. Zaleski, S. Jha, P. Batut, M. Chaisson, T. R. Gingeras, STAR: Ultrafast universal RNA-seq aligner. *Bioinformatics* **29**, 15–21 (2013). doi:10.1093/bioinformatics/bts635 Medline

67. A. Frankish, M. Diekhans, I. Jungreis, J. Lagarde, J. E. Loveland, J. M. Mudge, C. Sisu, J. C. Wright, J. Armstrong, I. Barnes, A. Berry, A. Bignell, C. Boix, S. Carbonell Sala, F. Cunningham, T. Di Domenico, S. Donaldson, I. T. Fiddes, C. García Girón, J. M. Gonzalez, T. Grego, M. Hardy, T. Hourlier, K. L. Howe, T. Hunt, O. G. Izuogu, R. Johnson, F. J. Martin, L. Martínez, S. Mohanan, P. Muir, F. C. P. Navarro, A. Parker, B. Pei, F. Pozo, F. C. Riera, M. Ruffier, B. M. Schmitt, E. Stapleton, M.-M. Suner, I. Sycheva, B. Uszczynska-Ratajczak, M. Y. Wolf, J. Xu, Y. T. Yang, A. Yates, D. Zerbino, Y. Zhang, J. S. Choudhary, M. Gerstein, R. Guigó, T. J. P. Hubbard, M. Kellis, B. Paten, M. L. Tress, P. Flicek, GENCODE 2021. *Nucleic Acids Res.* **49** (D1), D916–D923 (2021). doi:10.1093/nar/gkaa1087 Medline

68. R. Patro, G. Duggal, M. I. Love, R. A. Irizarry, C. Kingsford, Salmon provides fast and bias-aware quantification of transcript expression. *Nat. Methods* **14**, 417–419 (2017). doi:10.1038/nmeth.4197 Medline

69. M. D. Young, S. Behjati, SoupX removes ambient RNA contamination from droplet-based single-cell RNA sequencing data. *Gigascience* **9**, giaa151 (2020). doi:10.1093/gigascience/giaa151 Medline

70. Y. Hao, S. Hao, E. Andersen-Nissen, W. M. Mauck 3rd, S. Zheng, A. Butler, M. J. Lee, A. J. Wilk, C. Darby, M. Zager, P. Hoffman, M. Stoeckius, E. Papalexi, E. P. Mimitou, J. Jain, A. Srivastava, T. Stuart, L. M. Fleming, B. Yeung, A. J. Rogers, J. M. McElrath, C. A. Blish, R. Gottardo, P. Smibert, R. Satija, Integrated analysis of multimodal single-cell data. *Cell* **184**, 3573–3587.e29 (2021). doi:10.1016/j.cell.2021.04.048 Medline

71. G. Finak, A. McDavid, M. Yajima, J. Deng, V. Gersuk, A. K. Shalek, C. K. Slichter, H. W. Miller, M. J. McElrath, M. Prlic, P. S. Linsley, R. Gottardo, MAST: A flexible statistical framework for assessing transcriptional changes and characterizing heterogeneity in single-cell RNA sequencing data. *Genome Biol.* **16**, 278 (2015). doi:10.1186/s13059-015-0844-5 Medline

72. J. Feucht, J. Sun, J. Eyquem, Y.-J. Ho, Z. Zhao, J. Leibold, A. Dobrin, A. Cabriolu, M. Hamieh, M. Sadelain, Calibration of CAR activation potential directs alternative T cell fates and therapeutic potency. *Nat. Med.* **25**, 82–88 (2019). doi:10.1038/s41591-018-0290-5 Medline
